# Neuroinflammation and glycosylation-related cerebrospinal fluid proteins for predicting functional decline in amyotrophic lateral sclerosis: a proteomic study

**DOI:** 10.3389/fneur.2024.1418320

**Published:** 2024-11-18

**Authors:** Kimie Nakamura, Koji Fujita, Motohisa Suzuki, Akiyoshi Kunugi, Yoshihiko Hirozane, Tomonori Kunikata, Bitoku Takahashi, Genta Narazaki, Hirofumi Kondo, Shotaro Haji, Keisuke Hirai, Yuishin Izumi

**Affiliations:** ^1^Neuroscience Drug Discovery Unit, Research, Takeda Pharmaceutical Co., Ltd., Fujisawa, Japan; ^2^Department of Neurology, Tokushima University Graduate School of Biomedical Sciences, Tokushima, Japan; ^3^Rare Disease & LCM Laboratories, Group II, R&D Division, Daiichi Sankyo Co., Ltd., Tokyo, Japan

**Keywords:** amyotrophic lateral sclerosis, biomarker, cerebrospinal fluid, glycosylation, neuroinflammation, progression rate

## Abstract

**Background:**

The rate of disease progression varies widely among patients with amyotrophic lateral sclerosis (ALS). Prognostic assessment using biomarkers is highly anticipated to improve clinical trial design. We aimed to explore the cerebrospinal fluid (CSF) for prognostic biomarkers to predict future functional decline in patients with ALS.

**Methods:**

We collected CSF samples from 64 patients with ALS and 25 disease controls. The prospective progression rate was calculated using the Revised Amyotrophic Lateral Sclerosis Functional Rating Scale (ALSFRS-R) at CSF collection and in 6 months. The ALS patients were classified into slow, intermediate, and fast progression groups. We performed comprehensive proteomic analyses of the CSF samples. Factors with significant changes between slow and fast progression groups were investigated via receiver operating characteristic curve analyses. Moreover, the correlation of the CSF factors with progression rate was evaluated by multiple regression analyses.

**Results:**

In total, 26 proteins changed significantly (*p* < 0.05 and *q* < 0.10), with levels varying within a large dynamic range (fold change of >1.5 or < 0.5). A receiver operating characteristic curve analyses showed that the following proteins showed high discrimination power between slow and fast progression groups: glycoprotein non-metastatic melanoma protein B (GPNMB; area under the curve [AUC], 0.88), glial fibrillary acidic protein (AUC, 0.81), glypican-1 (GPC1; AUC, 0.79), alpha-1,6-mannosyl-glycoprotein 2-beta-N-acetylglucosaminyltransferase (AUC, 0.74), and chitinase-3-like protein 2 (CHI3L2; AUC, 0.73). Of these, GPNMB, GPC1, and CHI3L2 were significantly correlated to prognostic progression rate.

**Conclusion:**

This study demonstrated that CSF levels of neuroinflammation and glycosylation-related proteins were significantly correlated with prospective progression rates in patients with ALS. These proteins could be useful prognostic biomarkers for ALS.

## 1 Introduction

Amyotrophic lateral sclerosis (ALS) is characterized by motor neuron dysfunction and loss that leads to progressive muscular weakness and subsequent death due to respiratory failure, with a median survival of 3 years after onset (1). However, the rate of disease progression varies between patients; the disease durations are <1 year in some patients and 5–10 years in approximately 20% of patients (1). Hence, factors influencing the rate of progression can be targets of ALS drug development (1, 2). Although a few drugs have been approved by the US Food and Drug Administration (3–6), more effective medicines are needed to slow or, more desirably, halt the disease progression of ALS.

Challenges to the development of ALS drugs are partly posed by the widely varying disease progression rate (1, 2). Patients have been stratified based on the disease progression rate during the run-in periods of several months in many clinical trials (4, 5, 7, 8). However, the process inevitably delays the intervention, which advances the disease and likely reduces the potential efficacy of the investigational drug (7). Therefore, predicting future disease course at an early stage is critical.

The progression of ALS has been associated with multiple markers, including electrophysiological, neuroimaging, and biofluid measures (9–13). Of biofluids, cerebrospinal fluid (CSF) is directly in contact with central nervous tissues and can significantly reflect disease status (14). Patients with ALS have high CSF levels of neurofilament light chain (15–18) and chitinases, for example, chitotriosidase-1 (CHIT1), chitinase-3-like protein 1 (CHI3L1), and chitinase-3-like protein 2 (CHI3L2), which are cellular markers of macrophages and microglia (19–22). Each marker has been correlated with the disease progression rate and survival (15–17, 19–22). In most observational studies, the progression rate is calculated retrospectively using the Revised Amyotrophic Lateral Sclerosis Functional Rating Scale (ALSFRS-R) at one time point. It is reported, however, that the ALSFRS-R change from onset to baseline is not useful for stratifying subsequent progression (23). Therefore, it remains unclear whether the markers associated with the past progression rates can be used to predict the future progression rates. To address these issues, we explored CSF markers that are correlated with prospective functional decline in ALS using comprehensive proteomic analyses.

## 2 Materials and methods

### 2.1 Study population

The diagnosis of ALS was based on the updated Awaji criteria and divided into four categories: definite, probable, probable-laboratory-supported, and possible (24). The current study included 64 patients with ALS and 25 with other neurological diseases (control group; 13 with Alzheimer’s disease, 6 with progressive supranuclear palsy, 2 with spastic paraplegia, and 4 with frontotemporal lobar degeneration). The sample size was determined by referring to studies of unbiased CSF proteomic analyses to explore biomarker candidates including novel ones in patients with ALS (25). Disease onset was defined as the first occurrence of weakness reported by the patient (13). Based on the ALSFRS-R, the decline rate (ΔALSFRS-R) was calculated using the following formula: (ALSFRS-R score at the initial clinical visit − the ALSFRS-R score nearly 6 months after the initial clinical visit)/disease duration between the two time points in months. The 6-month assessment period was set based on the period often adopted for the evaluation of intervention in recent ALS clinical trials. The ALS samples were divided into three progression rate groups: slow progression rate (ALS-slow; ΔALSFRS-R score, <0.5), intermediate progression rate (ALS-intermediate; ΔALSFRS-R score, 0.5–1.0), and fast progression rate (ALS-fast; ΔALSFRS-R score, >1.0) (15). Written informed consent was obtained from each patient. Clinical data and CSF samples were collected at Tokushima University Hospital. This study was approved by the ethics committees of Tokushima University Hospital; Takeda Pharmaceutical Co., Ltd.; and Daiichi Sankyo Co., Ltd. This study was conducted in accordance with the Declaration of Helsinki.

### 2.2 CSF collection

We used the methodology applied in a previous study (26). In brief, CSF samples were collected via lumbar puncture within 3 months after the initial clinical visit and immediately stored at −80°C until analysis.

### 2.3 Proteomic analysis

Proteomic analysis was conducted at Caprion Biosciences, Inc. All samples were subjected to immunoaffinity depletion to remove highly abundant proteins using MARS-14 resin (Agilent Technologies, Santa Clara, CA). Immunodepleted samples were digested with trypsin (Promega, Madison, WI). After desalting, the sample was reconstituted with buffer containing 4% (v/v) acetonitrile and 0.2% formic acid. Analysis was performed using the nanoACQUITY UPLC System (Waters, Millford, MA) coupled online to a Q Exactive mass spectrometer (Thermo Fisher Scientific, Waltham, MA). Samples were loaded onto a BEH C18 300 Å column (150 μm × 100 mm, 1.7 μm; Waters) and separated using a gradient with (A) water +0.2% formic acid and (B) 95/5 (v/v) acetonitrile/dimethyl sulfoxide +0.2% formic acid. Eluted peptides were ionized using electrospray ionization in the positive ion mode. Precursor ion spectra were acquired in the range of 400–1,800 m/z with 70,000 full-width-at-half-maximum (FWHM) resolution at 200 m/z. Product ion scans were conducted in the mass range of 200–2,000 m/z with 17,500 FWHM resolution. The processing of mass spectrometry data was performed using the Rosetta Elucidator informatics platform (version 3.3.0.1; Rosetta Biosoftware). From the precursor ion spectra, the peak intensities for each detected isotope group were extracted across all samples. The product ion spectra were subjected to database search using the Mascot search engine (Matrix Science, version 2.5.1) and UniProt Human protein database (January 2017). The following search parameters were used: enzyme, trypsin; search type, tryptic; allowed missed cleavages, 2; peptide tolerance, 20 ppm; MS/MS tolerance, 0.05 Da; and variable modifications = deamidation (N), oxidation (M). The false discovery rate (FDR) was 5%, which was assessed using a decoy database of reverse sequences and was set to filter peptide identifications.

### 2.4 Metabolomic and lipidomic analyses

Metabolomic and lipidomic analyses were performed by Metabolon Inc., as previously described (27). In the metabolomic analysis, the samples were extracted with methanol and analyzed using the following methods: acidic reverse-phase chromatography optimized for hydrophilic or hydrophobic compounds in positive ionization mode, basic reverse-phase chromatography in negative ionization mode, and hydrophilic interaction chromatography in negative ionization mode. All methods used the ACQUITY UPLC (Waters) and Q Exactive mass spectrometers. Metabolites were identified by comparing purified standards and quantified via peak area integration. In the lipidomic analysis, lipids were extracted using the butanol–methanol method and analyzed using Sciex Selexion-5500 QTRAP (AB Sciex, Framingham, MA) with multiple reaction monitoring modes. Lipid species were quantified by taking the ratio of the signal intensity of each target to that of the internal standard.

### 2.5 Statistical analysis

To identify factors that were correlated with the decline rate (ΔALSFRS-R), the different disease progression subgroups were compared. Proteomic, metabolomic, and lipidomic data were analyzed using Welch’s *t*-test. A *p*-value of <0.05 was considered statistically significant. Multiple comparisons of datasets were adjusted by the FDR method, and each FDR was estimated using q-values, where a low value (<0.10) indicated high confidence. A multivariate linear mixed model analysis for proteomic data was conducted using Caprion’s Platform. Analysis of the area under the receiver operating characteristic (ROC) curve (AUC) was performed to assess the discrimination power. Categorical variables were compared using the chi-square test and continuous variables with the Tukey or Steel–Dwass test after the Bartlett test. Data analyses were performed using GraphPad Prism 9 (GraphPad Software, San Diego, CA) and SAS 9.3 (SAS Institute, Cary, NC). Multivariate regression analyses and linear mixed model predictions were calculated using EZR (Saitama Medical Center, Jichi Medical University, Saitama, Japan), which is a graphical user interface for R (The R Foundation for Statistical Computing, Vienna, Austria) (28).

## 3 Results

### 3.1 Clinical features

We collected CSF samples from 64 patients with ALS at the initial clinical visit. The samples within 6 years after collection were used for analysis. The patients were divided into three groups based on the functional decline rate (ΔALSFRS-R): ALS-slow (*n* = 24), ALS-intermediate (*n* = 14), and ALS-fast (*n* = 26). The clinical features of the patients are summarized in Table 1. No significant differences were noted in the clinical features of patients with ALS, except for age at disease onset (*p* < 0.01, ALS-slow vs. ALS-fast) and ALSFRS-R scores at the initial clinical visit (*p* < 0.05, ALS-slow vs. ALS-fast).

**Table 1:** Characteristics of the participants.

	ALS			Control
	Slow	Intermediate	Fast	
	(*n* = 26)	(*n* = 14)	(*n* = 24)	(*n* = 25)
	Mean ± SD or percentage			
ΔALSFRS-R	0.1 ± 0.3	0.7 ± 0.2	2.8 ± 1.9	
Body mass index	21.9 ± 3.1	21.8 ± 2.4	21.9 ± 2.4	
Male (%)	65	50	50	56
Age at onset (years)	61.8 ± 10.7	68.1 ± 9.3	70.7 ± 8.0**	62.6 ± 8.5^#^
Duration from onset to sample collection (months)	17.4 ± 12.8	13.4 ± 9.9	10.8 ± 7.4	
Duration from the initial clinical visit to CSF collection (months)	0.2 ± 0.6	0.4 ± 0.9	0.1 ± 0.3	
ALSFRS-R score at the initial clinical visit	42.4 ± 5.2	42.1 ± 4.5	39.0 ± 4.4*	
Duration of two-point ALSFRS-R scoring (months)	6.5 ± 3.7	7.4 ± 3.9	4.6 ± 3.1	
Site at onset				
Brachial amyotrophic diplegia	12%	29%	4%	
Bulbar	15%	14%	29%	
Upper limb	38%	36%	33%	
Lower limb	19%	14%	25%	
Others	15%	7%	8%	

Contingency tables were analyzed using the Tukey or Steel–Dwass test after the Bartlett test (*p < 0.05; **p < 0.01 vs. ALS-slow; and ^#^*p* < 0.05 vs. ALS-fast). Categorical variables were compared using the chi-square test.

### 3.2 CSF proteomics, metabolomics, and lipidomics

**Figure 1:**
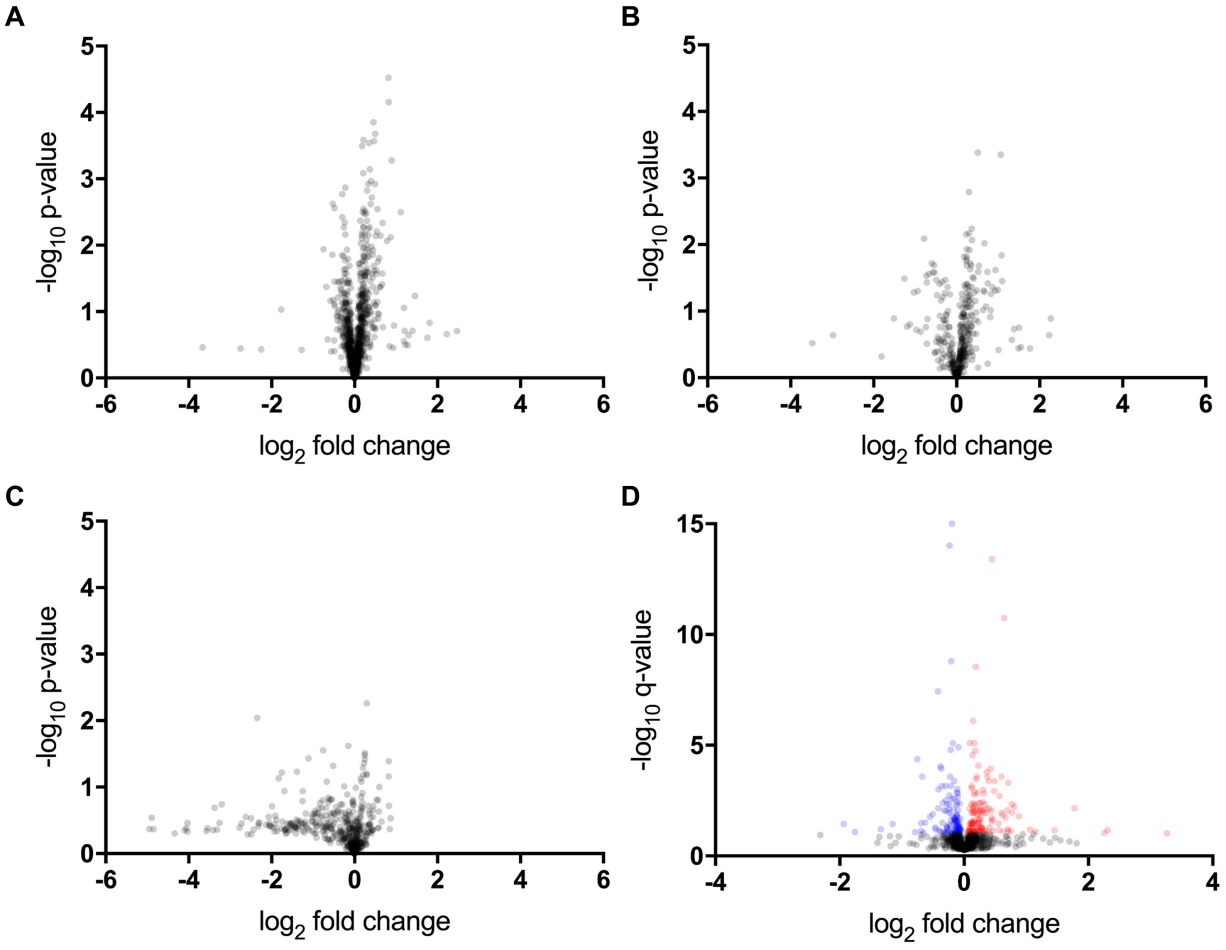
Proteomic, metabolomic, and lipidomic data. **(A–C)** Volcano plot showing the *p*-values (Welch’s *t*-test) that are correlated with fold changes in the slow progression rate (ALS-slow) versus fast progression rate (ALS-fast) groups (a, proteomic; b, metabolomic; c, lipidomic). **(D)** Multivariate analysis in the proteomic data. Volcano plot showing the q-values that are correlated with fold changes in the ALS-slow versus ALS-fast groups. In total, 259 proteins significantly differ (red and blue plots) (*p* < 0.05, *q* < 0.1).

The proteomic, metabolomic, and lipidomic analyses of CSF samples detected 1,012 proteins, 389 metabolites, and 735 lipids (Figures 1A–C and Supplementary Tables S1–S3). The proteomic analysis revealed that the levels of 14 proteins significantly differed between the ALS-slow and ALS-fast groups (*p* < 0.05, *q* < 0.1) (Supplementary Table S4). However, no significant differences were noted between the ALS-slow and ALS-intermediate groups or between the ALS-intermediate and ALS-fast groups. Based on the *p*-values alone, 53 metabolites and 7 lipids were differentially detected between the ALS-slow and ALS-fast groups (*p* < 0.05) (Supplementary Tables S5, S6). Because the progression group showed significant changes in proteins, further analysis focused on proteomic data. Considering age and sex, a multivariate linear mixed model analysis was conducted to assess proteomic data, and the levels of 259 proteins significantly changed between the ALS-slow and ALS-fast groups (*p* < 0.05, *q* < 0.1) (Figure 1D and Supplementary Table S7).

### 3.3 Proteins differently expressed in the slow and fast progression groups

**Figure 2:**
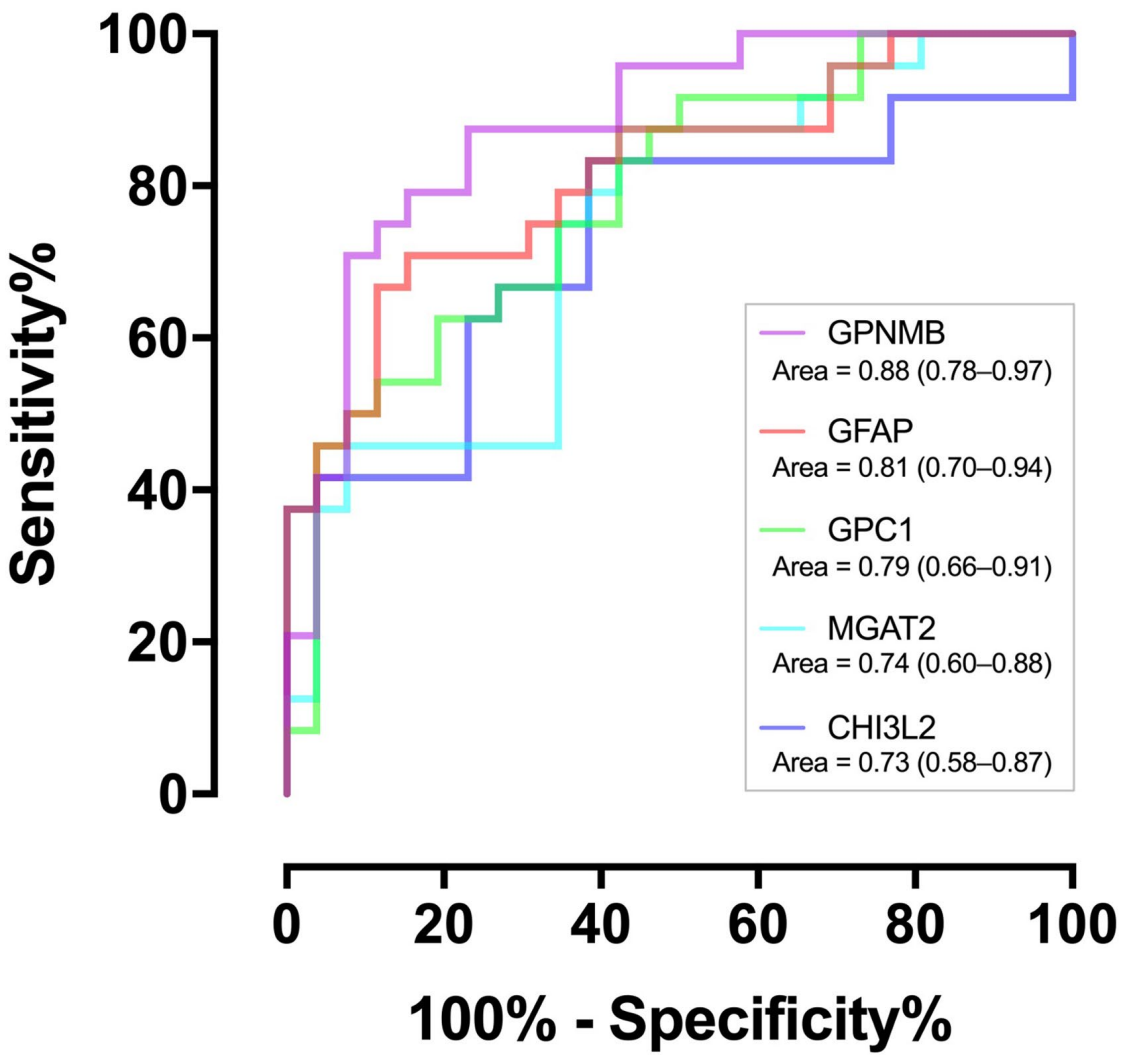
Discriminating performance of the five proteins. The area under the receiver operating characteristic curves with associated 95% confidence intervals.

Of the 259 proteins, 26 were found to have a large dynamic range of expression, comprising 22 upregulated proteins with a fold change of >1.5 and 4 downregulated proteins with a fold change of <0.5 (Table 2). To evaluate the discrimination power of the biomarker candidates, the AUC of these proteins was calculated. CHI3L2, which is reported to be a candidate marker for retrospective disease progression, showed a significant AUC (0.73, 95% confidence interval (95% CI), 0.58–0.87; *p* = 0.006) (19–21). Thus, we focused on CHI3L2 and proteins with AUC values higher than that of CHI3L2. Then, we found four additional proteins: glycoprotein nonmetastatic melanoma protein B (GPNMB; AUC, 0.88, 95% CI, 0.78–0.97; *p* < 0.001), glial fibrillary acidic protein (GFAP; AUC, 0.81, 95% CI, 0.70–0.94; *p* < 0.001), glypican-1 (GPC1; AUC, 0.79, 95% CI, 0.66–0.91; *p* < 0.001), and alpha-1,6-mannosyl-glycoprotein 2-beta-N-acetylglucosaminyltransferase (MGAT2; AUC, 0.74, 95% CI, 0.60–0.88; *p* = 0.003) (Figure 2).

**Table 2:** Proteins with significantly different expression levels between the slow and fast progression groups.

UniProt ID	Gene	Protein description	Fold change	*p*-value	*q*-value
DDR2_HUMAN	DDR2	Discoidin domain-containing receptor 2	9.60	0.045	0.095
ACES_HUMAN	ACHE	Acetylcholinesterase	4.95	0.025	0.069
FUCO_HUMAN	FUCA1	Tissue alpha-L-fucosidase	4.78	0.040	0.090
GPC1_HUMAN	GPC1	Glypican-1	3.43	0.001	0.007
CLIC1_HUMAN	CLIC1	Chloride intracellular channel protein 1	2.74	0.024	0.069
MGAT2_HUMAN	MGAT2	Alpha-1,6-mannosyl-glycoprotein 2-beta-N-acetylglucosaminyltransferase	2.21	0.035	0.082
CD177_HUMAN	CD177	CD177 antigen	2.08	0.023	0.065
CHIT1_HUMAN	CHIT1	Chitotriosidase-1	1.84	0.003	0.016
GFAP_HUMAN	GFAP	Glial fibrillary acidic protein	1.75	0.001	0.006
APOC2_HUMAN	APOC2	Apolipoprotein C-II	1.71	0.002	0.011
FUCO2_HUMAN	FUCA2	Plasma alpha-L-fucosidase	1.70	0.001	0.005
POSTN_HUMAN	POSTN	Periostin	1.69	0.032	0.079
G3P_HUMAN	GAPDH	Glyceraldehyde-3-phosphate dehydrogenase	1.64	0.029	0.076
MCFD2_HUMAN	MCFD2	Multiple coagulation factor deficiency protein 2	1.63	0.000	0.000
APOC1_HUMAN	APOC1	Apolipoprotein C-I	1.63	0.021	0.061
GPNMB_HUMAN	GPNMB	Transmembrane glycoprotein NMB	1.62	0.001	0.009
CP089_HUMAN	C16orf89	UPF0764 protein C16orf89	1.59	0.004	0.019
CH3L2_HUMAN	CHI3L2	Chitinase-3-like protein 2	1.57	0.000	0.000
CHST8_HUMAN	CHST8	Carbohydrate sulfotransferase 8	1.55	0.032	0.079
HBB_HUMAN	HBB	Hemoglobin subunit beta	1.53	0.003	0.014
SORC3_HUMAN	SORCS3	VPS10 domain-containing receptor SorCS3	1.52	0.002	0.011
LDHB_HUMAN	LDHB	L-lactate dehydrogenase B chain	1.52	0.000	0.000
NPDC1_HUMAN	NPDC1	Neural proliferation differentiation and control protein 1	0.45	0.010	0.036
K1C16_HUMAN	KRT16	Keratin, type I cytoskeletal 16	0.39	0.021	0.061
CPSF1_HUMAN	CPSF1	Cleavage and polyadenylation specificity factor subunit 1	0.30	0.035	0.082
EGFR_HUMAN	EGFR	Epidermal growth factor receptor	0.26	0.010	0.036

The list of 26 proteins with significantly different expression levels (*p* < 0.05, *q* < 0.1) and large fold changes in expression (<0.5 or > 1.5).

### 3.4 Correlation between the protein levels and prospective progression rates

**Figure 3:**
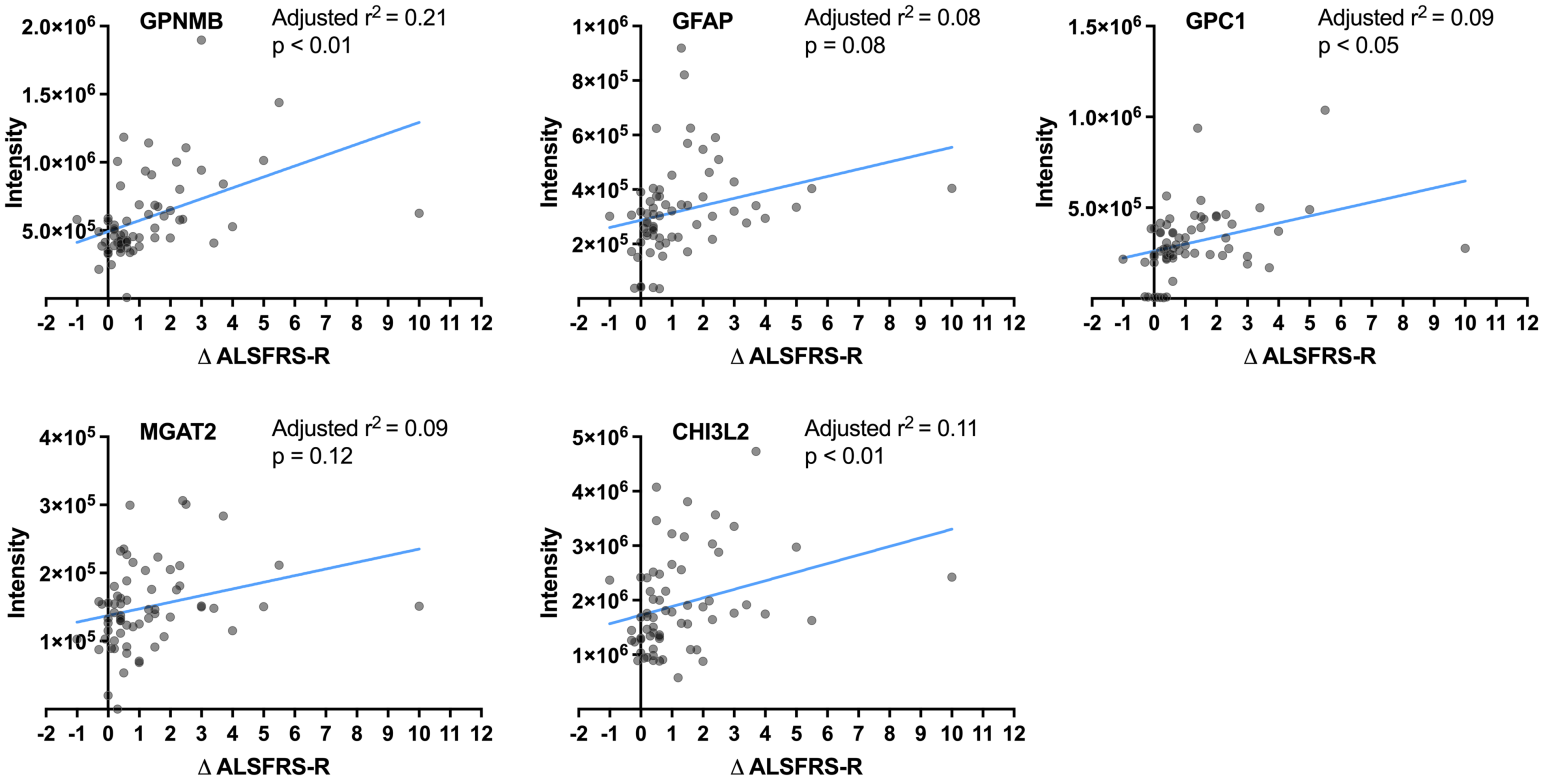
Correlation between the protein levels and progression rates. The lines show linear regression fits. The adjusted *r*^2^ and *p*-value are calculated by multivariate regression analysis considering age.

Correlations between the CSF levels of the five proteins and progression rate were analyzed in samples including ALS-intermediate by multivariate regression models considering age and ALSFRS-R score at the initial clinical visit. The values of GPNMB (adjusted *r*^2^ = 0.21, *p* < 0.01), GPC1 (adjusted *r*^2^ = 0.09, *p* < 0.05), and CHI3L2 (adjusted *r*^2^ = 0.11, *p* < 0.01) were significantly correlated with the progression rate while those of GFAP (adjusted *r*^2^ = 0.08, *p* = 0.08) and MGAT2 (adjusted *r*^2^ = 0.09, *p* = 0.12) were not (Figure 3). None were correlated with age; the value of MGAT2 was correlated with the initial ALSFRS-R score. In comparison with the control by multivariate regression analyses considering the age, the GPNMB level was high in the ALS-fast group (*p* < 0.001) and the CHI3L2 level was high in the ALS-fast and ALS-intermediate groups (both *p* < 0.05) (Supplementary Figure S1).

Additionally, to stratify any progression rate, the disease progression rate was estimated using a linear mixed model that included the effect of the z-score of each protein level, age, and ALSFRS-R score at the initial visit (Supplementary Table S8). Among GPNMB, GPC1, and CHI3L2, the CHI3L2 level showed significant correlation with progression rate (*p* < 0.05).

### 3.5 Comparisons between the slow, intermediate, and fast progression groups

**Figure 4:**
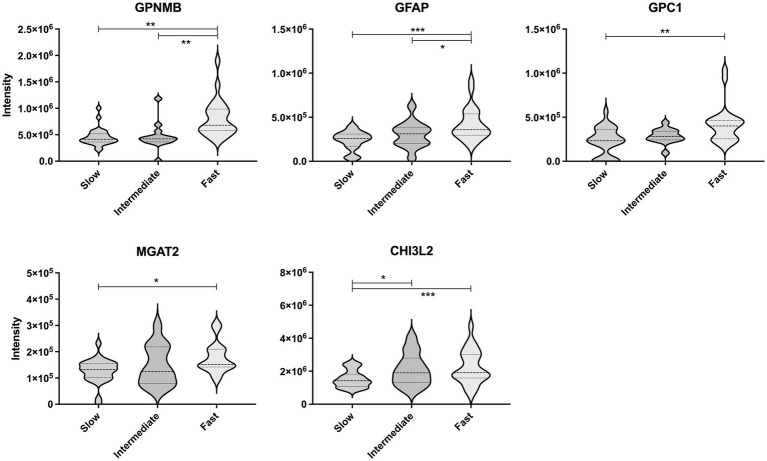
Comparison of the protein levels between the amyotrophic lateral sclerosis progression groups. The violin plot represents the weight distributions of variables. The dashed line in each plot represents the median, and the dotted line represents the 25th and 75th percentiles. *p < 0.05; **p < 0.01; and ***p < 0.0001, using the multivariate regression analyses considering age.

Next, the CSF levels of the five proteins were compared between the ALS progression groups using multivariate regression analyses considering age and ALSFRS-R score at the initial clinical visit (Figure 4). The levels of GPNMB (*p* < 0.01 and *p* < 0.01) and GFAP (*p* < 0.001 and *p* < 0.05) were significantly higher in the ALS-fast group than in the ALS-slow and ALS-intermediate groups. GPC1 was significantly higher in the ALS-fast group than in the ALS-slow (*p* < 0.01). The MGAT2 level was significantly different between the ALS-slow and ALS-fast groups (*p* < 0.05); however, the level was also negatively correlated to the ALSFRS-R score. The CHI3L2 level was significantly higher in the ALS-intermediate (*p* < 0.05) and ALS-fast (*p* < 0.001) groups than in the ALS-slow group.

## 4 Discussion

This study explored CSF prognostic biomarkers for predicting future functional decline in ALS using a comprehensive proteomic analysis. Several clinical studies retrospectively calculate the progression rate based on the ALSFRS-R score at the initial clinical visit. In contrast, this study prospectively evaluated the progression rate based on the ALSFRS-R scores obtained at two time points, that is, at CSF collection and at 6 months.

Many of the 26 proteins with significant changes are functionally involved in glycan modifications, including carbohydrate sulfotransferase 8 (29), tissue alpha-L-fucosidase, plasma alpha-L-fucosidase (30), and glyceraldehyde-3-phosphate dehydrogenase (31). Furthermore, CHI3L2 plays a role in the binding and degradation of oligosaccharides and other glycans (32). Recently, N-glycosylation pattern changes were reported in CSF glycoproteins (33), CSF immunoglobulin G (IgG) (34), and serum IgG (35) in patients with ALS. Moreover, ALS-causing mutations were found in the substrate-binding domain of glycosyltransferase 8 domain-containing protein 1 (36). Hence, the proteins that were correlated with glycan modification may be related to the pathophysiology of ALS progression.

While CHI3L2 has been associated with the retrospective progression rate (19–21), it was also related to the prospective progression rate in this study. In line with this, higher CSF CHI3L2 levels were reportedly associated with shorter survival in patients with ALS (37). The cellular source of CHI3L2 has not been clarified in ALS; however, CSF CHI3L2 was parallelly elevated with CSF CHI3L1, which is related to neuroinflammation and expressed in microglia of the spinal cord of patients with ALS (19, 20, 22, 37). CHI3L1 and CHIT1, members of the chitinase family, were also changed with retrospective progression rate in several studies. Although CHIT1 was listed in the 26 proteins with significant changes in this study, it was excluded from the candidates due to the low discrimination power (AUC, 0.54).

In addition, we showed that GPNMB and GPC1 had higher relationships with future fast progression than CHI3L2. Thus, these proteins were highlighted as candidate predictive markers of rapid progression in ALS.

GPNMB is a type I transmembrane glycoprotein with a neuroprotective effect (38). SOD1-G93A mutation inhibited glycosylation of GPNMB, which increased motor neuron vulnerability, whereas extracellular fragments of GPNMB secreted from activated astrocytes attenuated the neurotoxicity in neural cells (38). GPNMB is also involved in pre-mRNA splicing, endoplasmic reticulum stress regulation (27), and endo-lysosome function (39, 40). Although precise localization of GPNMB has still been under discussion in patients with ALS (37, 38), the GPNMB level increased in the CSF and plasma in ALS (37–39). Moreover, higher CSF GPNMB levels were correlated with shorter survival in patients with ALS (39–44), which is consistent with our findings of faster progression.

GPC1 is a glycosylphosphatidylinositol-anchored protein with a high affinity to the slit homolog 2 (SLIT2) protein, which has a repulsive guidance effect on growing axons (45). GPC1 and SLIT2 mRNA were co-expressed in reactive astrocytes in the injured brain or spinal cord of adult mice (46). Therefore, GPC1 acting with SLIT2 may prevent axonal regeneration (46). Whilst, a glypican ortholog in *Drosophila melanogaster* was associated with synaptic bouton growth at the neuromuscular junction (47). GPC1 was also reported to express in neurons and regulate brain size through fibroblast growth factor signaling in mice (48), which indicated GPC1 might directly regulate neural function. These findings suggest that an increase of GPC1 may have compensatory neuroprotective mechanisms.

If the prognostic biomarker candidates revealed in this study are validated, their use in patient stratification would improve clinical trials. Currently, several ALS clinical trials have set run-in periods, commonly of 12 weeks, to evaluate the rate of functional decline and exclude patients with extremely fast- or slow-progressing ALS. Although important for appropriate patient enrollment, this period delays the administration of investigational drugs, which could adversely affect participants in a trial because early intervention may be critical (4, 7). The CSF markers detected in this study could improve clinical trials by facilitating the assessment of future progression rates at the initial evaluation and by substantially shortening the time before allocation.

This study has several limitations. First, the ALS-fast group was older and had lower ALSFRS-R scores than the ALS-slow group. These features have been previously reported in ALS with fast progression (9, 49, 50). Nonetheless, we performed a multivariate linear mixed model analysis and addressed concerns related to the differences. Second, the samples in this study were collected only from Japanese patients. For generalization of the findings, larger sample size and more diverse cohort would be encouraged in future studies. Third, the obtained proteomic data were not quantified using the calibration curve method. The accuracy of the prediction model is limited to estimate the progression rate, although the degree of change in the quantitative concentrations of our benchmark protein, CHI3L2, was close to the change in this study (37). Quantitative studies with an independent cohort and larger set of CSF samples, such as studies using multiple reaction monitoring assays, would further elucidate the prognostic utility in the clinical uses of the nominated proteins. Finally, we set the assessment period of 6 months based on the typical intervention period in ALS clinical trials. Longer-term evaluation and longitudinal data might further reveal the utility of these markers.

In conclusion, we showed that the CSF values of neuroinflammation and glycosylation-related proteins were correlated with prospective functional decline in patients with ALS. Although further quantitative studies in independent cohorts are required, these proteins are potentially useful as prognostic biomarkers for ALS.

## Data availability statement

The mass spectrometry proteomics data have been deposited to the ProteomeXchange Consortium via the PRIDE (51) partner repository with the dataset identifier PXD057276, http://www.ebi.ac.uk/pride/archive/projects/PXD057276.

## Ethics statement

The studies involving humans were approved by the Ethics Committees of Tokushima University Hospital, Takeda Pharmaceutical Co., Ltd. and Daiichi Sankyo Co., Ltd. The studies were conducted in accordance with the local legislation and institutional requirements. The human samples used in this study were acquired from a by-product of routine care. The participants provided their written informed consent to participate in this study.

## Author contributions

KN: Conceptualization, Formal analysis, Investigation, Visualization, Writing – original draft, Writing – review & editing. KF: Resources, Supervision, Visualization, Writing – review & editing, Formal analysis, Investigation. MS: Conceptualization, Formal analysis, Investigation, Writing – review & editing. AK: Conceptualization, Formal analysis, Investigation, Writing – review & editing. YH: Formal analysis, Investigation, Writing – review & editing. TK: Conceptualization, Writing – review & editing. BT: Conceptualization, Writing – review & editing. GN: Conceptualization, Writing – review & editing. HK: Conceptualization, Writing – review & editing. SH: Resources, Writing – review & editing. KH: Writing – review & editing, Conceptualization, Formal analysis, Investigation. YI: Formal analysis, Investigation, Resources, Writing – review & editing, Funding acquisition, Conceptualization.
