# Supplementary material for: Neuroinflammation and glycosylation-related cerebrospinal fluid proteins for predicting functional decline in amyotrophic lateral sclerosis: a proteomic study
Source: Front Neurol. 2024 Nov 18;15:1418320. doi: 10.3389/fneur.2024.1418320 (PMC11608993; doi:10.3389/fneur.2024.1418320)
Supplement: Supplementary file 1 [file Table_1.PDF]

Supplementary Material

1 Supplementary Table S1. Factors detected via the proteomic analysis.

Protein	Protein description	Fold change	p-value	q-value
VGF	Neurosecretory protein VGF	0.92	0.000	0.001
CHL1	Neural cell adhesion molecule L1-like protein	0.93	0.000	0.001
CHGB	Secretogranin-1	1.05	0.140	0.183
CHGA	Chromogranin-A	0.86	0.000	0.000
SCG3	Secretogranin-3	0.94	0.009	0.034
C9	Complement component C9	0.90	0.007	0.027
SERPINC1	Antithrombin-III	0.86	0.000	0.001
ITIH2	Inter-alpha-trypsin inhibitor heavy chain H2	0.93	0.020	0.058
C6	Complement component C6	0.93	0.003	0.017
CFB	Complement factor B	0.94	0.013	0.044
FN1	Fibronectin	1.00	0.957	0.509
HPX	Hemopexin	0.91	0.004	0.019
FAT2	Protocadherin Fat 2	1.09	0.010	0.036
SCG2	Secretogranin-2	0.94	0.031	0.077
NEO1	Neogenin	1.06	0.000	0.000
NRCAM	Neuronal cell adhesion molecule	0.95	0.070	0.127
CP	Ceruloplasmin	1.00	0.791	0.465
PLG	Plasminogen	0.93	0.044	0.093

MCAM	Cell surface glycoprotein MUC18	1.06	0.001	0.007
FAM3C	Protein FAM3C	0.95	0.021	0.061
ITIH4	Inter-alpha-trypsin inhibitor heavy chain H4	1.07	0.050	0.102
LSAMP	Limbic system-associated membrane protein	0.94	0.000	0.000
PAM	Peptidyl-glycine alpha-amidating monooxygenase	1.01	0.623	0.413
PLXNB2	Plexin-B2	1.10	0.000	0.000
ENO1	Alpha-enolase	1.09	0.001	0.005
SEMA7A	Semaphorin-7A	0.99	0.662	0.428
SERPINF2	Alpha-2-antiplasmin	0.97	0.469	0.360
APOE	Apolipoprotein E	1.36	0.000	0.000
GSN	Gelsolin	0.98	0.299	0.277
CTSD	Cathepsin D	0.99	0.721	0.444
ALDOA	Fructose-bisphosphate aldolase A	1.07	0.017	0.053
CPB2	Carboxypeptidase B2	1.00	0.986	0.517
OGN	Mimecan	1.11	0.001	0.009
F2	Prothrombin	0.87	0.000	0.003
CFH	Complement factor H	0.91	0.007	0.028
NELL2	Protein kinase C-binding protein NELL2	0.92	0.013	0.044
RELN	Reelin	1.13	0.001	0.005
CFI	Complement factor I	0.95	0.048	0.099
THY1	Thy-1 membrane glycoprotein	0.92	0.001	0.005

PTPRN	Receptor-type tyrosine-protein phosphatase-like N	0.90	0.005	0.023
CNTN1	Contactin-1	1.03	0.071	0.127
TGOLN2	Trans-Golgi network integral membrane protein 2	0.95	0.000	0.004
APLP1	Amyloid-like protein 1	1.02	0.457	0.355
PPIB	Peptidyl-prolyl cis-trans isomerase B	0.94	0.006	0.025
AHSG	Alpha-2-HS-glycoprotein	1.04	0.625	0.413
NEGR1	Neuronal growth regulator 1	0.92	0.031	0.078
PTPRG	Receptor-type tyrosine-protein phosphatase gamma	1.01	0.719	0.444
DCC	Netrin receptor DCC	0.95	0.073	0.130
ASAH1	Acid ceramidase	0.94	0.109	0.161
CDH13	Cadherin-13	0.90	0.000	0.000
CACNA2D1	Voltage-dependent calcium channel subunit alpha-2/delta-1	0.95	0.269	0.260
SUSD5	Sushi domain-containing protein 5	0.97	0.128	0.176
RTN4RL2	Reticulon-4 receptor-like 2	0.92	0.006	0.026
MAPT	Microtubule-associated protein tau	0.84	0.210	0.231
C8B	Complement component C8 beta chain	1.00	0.938	0.505
YWHAZ	14-3-3 protein zeta/delta	1.06	0.109	0.161
KRT9	Keratin, type I cytoskeletal 9	0.99	0.957	0.509
NRXN1	Neurexin-1-beta	0.96	0.064	0.120
SERPINF1	Pigment epithelium-derived factor	0.93	0.000	0.001

CHI3L2	Chitinase-3-like protein 2	1.57	0.000	0.000
DCN	Decorin	1.08	0.028	0.073
GC	Vitamin D-binding protein	0.85	0.001	0.008
CADM3	Cell adhesion molecule 3	0.99	0.669	0.431
CHIT1	Chitotriosidase-1	1.84	0.003	0.016
CLSTN1	Calsyntenin-1	0.93	0.001	0.009
C2	Complement C2	1.11	0.000	0.004
PCOLCE	Procollagen C-endopeptidase enhancer 1	0.93	0.000	0.002
COL6A1	Collagen alpha-1(VI) chain	1.01	0.645	0.421
GAP43	Neuromodulin	0.98	0.567	0.395
COL6A3	Collagen alpha-3(VI) chain	1.01	0.771	0.459
HBA1	Hemoglobin subunit alpha	1.40	0.000	0.001
PTPRS	Receptor-type tyrosine-protein phosphatase S	0.99	0.817	0.472
CHI3L1	Chitinase-3-like protein 1	1.07	0.319	0.287
NCAM1	Neural cell adhesion molecule 1	0.95	0.052	0.103
NOV	Protein NOV homolog	1.14	0.206	0.229
PCSK1N	ProSAAS	0.98	0.675	0.434
NUCB1	Nucleobindin-1	0.95	0.012	0.042
LUM	Lumican	0.93	0.010	0.037
ANG	Angiogenin	1.01	0.942	0.506
CEL	Bile salt-activated lipase	1.06	0.636	0.418

SELENBP1	Selenium-binding protein 1	0.95	0.019	0.058
AGA	N(4)-(beta-N-acetylglucosaminyl)-L-asparaginase	0.89	0.000	0.003
A1BG	Alpha-1B-glycoprotein	0.99	0.787	0.465
KRT1	Keratin, type II cytoskeletal 1	0.94	0.480	0.364
QSOX1	Sulfhydryl oxidase 1	1.01	0.566	0.395
ENPP2	Ectonucleotide pyrophosphatase/phosphodiesterase family member 2	1.09	0.048	0.099
IL6ST	Interleukin-6 receptor subunit beta	1.01	0.824	0.474
RGMB	RGM domain family member B	1.00	0.910	0.494
AZGP1	Zinc-alpha-2-glycoprotein	0.85	0.001	0.005
LMAN2	Vesicular integral-membrane protein VIP36	1.01	0.756	0.454
APOA4	Apolipoprotein A-IV	0.89	0.115	0.165
YWHAE	14-3-3 protein epsilon	0.99	0.910	0.494
NTM	Neurotrimin	0.96	0.093	0.149
RBP4	Retinol-binding protein 4	0.75	0.000	0.000
AGAP3	Arf-GAP with GTPase, ANK repeat and PH domain-containing protein 3	0.95	0.619	0.412
FTH1	Ferritin heavy chain	0.97	0.579	0.398
CTBS	Di-N-acetylchitobiase	0.87	0.000	0.000
LRP8	Low-density lipoprotein receptor-related protein 8	1.17	0.041	0.090
CNTNAP2	Contactin-associated protein-like 2	1.00	0.912	0.494
NPTX2	Neuronal pentraxin-2	1.08	0.519	0.380

CRTAC1	Cartilage acidic protein 1	0.92	0.007	0.027
CBLN1	Cerebellin-1	0.99	0.888	0.489
GOT1	Aspartate aminotransferase, cytoplasmic	1.14	0.000	0.000
PRAP1	Proline-rich acidic protein 1	1.08	0.085	0.143
MYDGF	Myeloid-derived growth factor	0.98	0.558	0.394
IGFALS	Insulin-like growth factor-binding protein complex acid labile subunit	0.77	0.000	0.000
MEGF8	Multiple epidermal growth factor-like domains protein 8	1.08	0.057	0.110
MYOC	Myocilin	1.26	0.079	0.137
CA1	Carbonic anhydrase 1	0.93	0.592	0.402
F5	Coagulation factor V	0.96	0.349	0.303
KIT	Mast/stem cell growth factor receptor Kit	1.04	0.215	0.232
QDPR	Dihydropteridine reductase	0.95	0.162	0.199
CPN1	Carboxypeptidase N catalytic chain	0.86	0.043	0.093
C8A	Complement component C8 alpha chain	0.94	0.214	0.231
GDI1	Rab GDP dissociation inhibitor alpha	1.12	0.006	0.026
NID2	Nidogen-2	1.03	0.478	0.364
SCG5	Neuroendocrine protein 7B2	1.09	0.000	0.001
COL1A1	Collagen alpha-1(I) chain	0.96	0.130	0.176
AFM	Afamin	0.99	0.907	0.494
GPR158	Probable G-protein coupled receptor 158	1.01	0.819	0.473
IGFBP6	Insulin-like growth factor-binding protein 6	0.91	0.005	0.023

OMD	Osteomodulin	1.16	0.000	0.001
LCP1	Plastin-2	1.02	0.477	0.364
PROS1	Vitamin K-dependent protein S	1.05	0.302	0.277
ZP2	Zona pellucida sperm-binding protein 2	0.68	0.739	0.448
SERPINB1	Leukocyte elastase inhibitor	0.98	0.529	0.384
MB	Myoglobin	1.14	0.349	0.303
SEL1L	Protein sel-1 homolog 1	0.86	0.075	0.132
ITIH1	Inter-alpha-trypsin inhibitor heavy chain H1	0.93	0.093	0.149
CNTN3	Contactin-3	0.99	0.835	0.476
APCS	Serum amyloid P-component	0.87	0.046	0.096
NRXN3	Neurexin-3	1.05	0.113	0.163
CTSF	Cathepsin F	1.12	0.033	0.079
HRNR	Hornerin	0.89	0.830	0.475
CANT1	Soluble calcium-activated nucleotidase 1	0.96	0.004	0.020
FBLN7	Fibulin-7	1.06	0.214	0.231
HK1	Hexokinase-1	1.07	0.354	0.303
ADAM22	Disintegrin and metalloproteinase domain-containing protein 22	1.05	0.080	0.137
LCAT	Phosphatidylcholine-sterol acyltransferase	1.03	0.336	0.295
CD14	Monocyte differentiation antigen CD14	1.08	0.009	0.034
CDC37	Hsp90 co-chaperone Cdc37	0.90	0.115	0.165
CLCNKB	Chloride channel protein ClC-Kb	1.00	0.943	0.506

MST1	Hepatocyte growth factor-like protein	0.94	0.294	0.273
PXDN	Peroxidasin homolog	1.12	0.148	0.188
RTN4R	Reticulon-4 receptor	0.92	0.164	0.199
GFRA2	GDNF family receptor alpha-2	0.90	0.005	0.022
CDH7	Cadherin-7	1.04	0.237	0.248
TPP1	Tripeptidyl-peptidase 1	0.97	0.368	0.312
AMBP	Protein AMBP	0.71	0.002	0.013
GPX3	Glutathione peroxidase 3	1.07	0.046	0.096
SPON1	Spondin-1	0.97	0.198	0.224
TPPP3	Tubulin polymerization-promoting protein family member 3	1.04	0.569	0.395
SEMA3B	Semaphorin-3B	1.11	0.427	0.341
YWHAG	14-3-3 protein gamma	1.15	0.001	0.008
NAGLU	Alpha-N-acetylglucosaminidase	0.97	0.527	0.383
SERPINA4	Kallistatin	1.00	0.947	0.507
LRTM2	Leucine-rich repeat and transmembrane domain-containing protein 2	0.99	0.990	0.519
MAN2B1	Lysosomal alpha-mannosidase	1.13	0.041	0.090
PLXDC1	Plexin domain-containing protein 1	0.95	0.107	0.161
QPCT	Glutaminyl-peptide cyclotransferase	0.99	0.659	0.427
HBB	Hemoglobin subunit beta	1.53	0.003	0.014
OPCML	Opioid-binding protein/cell adhesion molecule	0.93	0.378	0.317
ROBO1	Roundabout homolog 1	0.91	0.108	0.161

TMSB4X	Thymosin beta-4	0.98	0.506	0.375
MMP17	Matrix metalloproteinase-17	0.86	0.114	0.164
CPE	Carboxypeptidase E	1.10	0.000	0.000
WFIKKN2	WAP, Kazal, immunoglobulin, Kunitz and NTR domain-containing protein 2	1.08	0.067	0.123
N4BP2L1	NEDD4-binding protein 2-like 1	0.85	0.379	0.317
EFNA5	Ephrin-A5	0.94	0.690	0.440
PGRMC1	Membrane-associated progesterone receptor component 1	0.99	0.770	0.458
TMSB10	Thymosin beta-10	0.98	0.729	0.445
COL18A1	Collagen alpha-1(XVIII) chain	0.96	0.051	0.102
FTL	Ferritin light chain	0.90	0.071	0.128
DKK3	Dickkopf-related protein 3	0.86	0.014	0.046
MMP2	72 kDa type IV collagenase	0.94	0.030	0.076
FSTL4	Follistatin-related protein 4	1.13	0.092	0.149
SDF4	45 kDa calcium-binding protein	1.04	0.048	0.099
ATP6AP2	Renin receptor	1.29	0.000	0.000
TIMP2	Metalloproteinase inhibitor 2	0.96	0.324	0.289
NCAN	Neurocan core protein	1.05	0.103	0.157
OAF	Out at first protein homolog	1.01	0.868	0.484
TNXB	Tenascin-X	0.97	0.252	0.251
ITIH5	Inter-alpha-trypsin inhibitor heavy chain H5	1.03	0.280	0.266
MDH1	Malate dehydrogenase, cytoplasmic	1.30	0.000	0.000

CADM1	Cell adhesion molecule 1	0.92	0.015	0.049
CSPG5	Chondroitin sulfate proteoglycan 5	0.98	0.723	0.445
PCDH17	Protocadherin-17	1.03	0.319	0.287
SST	Somatostatin	0.99	0.848	0.481
CACNA2D2	Voltage-dependent calcium channel subunit alpha-2/delta-2	1.05	0.348	0.303
CDH8	Cadherin-8	0.95	0.173	0.206
BLMH	Bleomycin hydrolase	1.09	0.145	0.187
KNG1	Kininogen-1	0.93	0.285	0.269
LGALS3BP	Galectin-3-binding protein	0.85	0.000	0.000
BASP1	Brain acid soluble protein 1	0.94	0.082	0.140
SLITRK1	SLIT and NTRK-like protein 1	0.94	0.288	0.271
L1CAM	Neural cell adhesion molecule L1	0.97	0.301	0.277
AGT	Angiotensinogen	1.03	0.385	0.319
SEZ6L2	Seizure 6-like protein 2	1.06	0.437	0.345
PCSK2	Neuroendocrine convertase 2	1.09	0.457	0.355
TCN2	Transcobalamin-2	1.07	0.261	0.254
AGRN	Agrin	0.97	0.258	0.254
COL1A2	Collagen alpha-2(I) chain	0.93	0.057	0.110
KRT2	Keratin, type II cytoskeletal 2 epidermal	0.73	0.021	0.061
PPIA	Peptidyl-prolyl cis-trans isomerase A	1.18	0.000	0.004
MOG	Myelin-oligodendrocyte glycoprotein	0.94	0.207	0.229

PRELP	Prolargin	1.37	0.005	0.023
SEZ6L	Seizure 6-like protein	0.82	0.002	0.009
C3	Complement C3	0.87	0.000	0.000
LRP4	Low-density lipoprotein receptor-related protein 4	0.93	0.452	0.353
PTPRN2	Receptor-type tyrosine-protein phosphatase N2	0.94	0.432	0.344
MIA	Melanoma-derived growth regulatory protein	0.96	0.487	0.367
PDGFB	Platelet-derived growth factor subunit B	0.99	0.831	0.475
ICAM5	Intercellular adhesion molecule 5	0.95	0.121	0.168
ADAMTS4	A disintegrin and metalloproteinase with thrombospondin motifs 4	0.98	0.426	0.340
METRNL	Meteorin-like protein	1.07	0.302	0.277
CNTFR	Ciliary neurotrophic factor receptor subunit alpha	1.07	0.031	0.077
GDA	Guanine deaminase	1.49	0.000	0.002
PCDH1	Protocadherin-1	1.07	0.381	0.318
TPBG	Trophoblast glycoprotein	1.05	0.647	0.422
CHID1	Chitinase domain-containing protein 1	1.01	0.817	0.472
CSF1R	Macrophage colony-stimulating factor 1 receptor	1.09	0.140	0.183
HSPA13	Heat shock 70 kDa protein 13	1.14	0.144	0.186
PSAP	Prosaposin	1.07	0.087	0.145
AXL	Tyrosine-protein kinase receptor UFO	1.06	0.010	0.036
PCDHAC2	Protocadherin alpha-C2	0.92	0.168	0.202

CAMK2A	Calcium/calmodulin-dependent protein kinase type II subunit alpha	1.09	0.267	0.258
HSPG2	Basement membrane-specific heparan sulfate proteoglycan core protein	1.05	0.102	0.156
CDH9	Cadherin-9	0.90	0.323	0.289
MAN2A2	Alpha-mannosidase 2x	1.09	0.009	0.033
OMG	Oligodendrocyte-myelin glycoprotein	0.98	0.729	0.445
PLXNB1	Plexin-B1	1.00	0.985	0.517
CA10	Carbonic anhydrase-related protein 10	1.11	0.258	0.254
HEXA	Beta-hexosaminidase subunit alpha	0.99	0.721	0.444
ALDOC	Fructose-bisphosphate aldolase C	1.04	0.032	0.079
IGSF21	Immunoglobulin superfamily member 21	0.94	0.264	0.256
NCAM2	Neural cell adhesion molecule 2	0.95	0.029	0.074
MASP1	Mannan-binding lectin serine protease 1	1.12	0.104	0.158
PEPD	Xaa-Pro dipeptidase	0.81	0.765	0.456
PCMT1	Protein-L-isoaspartate(D-aspartate) O-methyltransferase	1.00	0.980	0.516
CTSS	Cathepsin S	1.05	0.032	0.079
IGFBP7	Insulin-like growth factor-binding protein 7	0.92	0.231	0.244
PDYN	Proenkephalin-B	0.92	0.400	0.327
MARCO	Macrophage receptor MARCO	1.11	0.533	0.385
SERPINB6	Serpin B6	1.04	0.098	0.151
CDH15	Cadherin-15	1.11	0.201	0.225

EEA1	Early endosome antigen 1	1.11	0.170	0.203
EPHB6	Ephrin type-B receptor 6	0.97	0.571	0.395
FSTL1	Follistatin-related protein 1	0.99	0.825	0.474
FXYD6	FXYD domain-containing ion transport regulator 6	0.84	0.177	0.208
HABP2	Hyaluronan-binding protein 2	1.10	0.033	0.080
LHPP	Phospholysine phosphohistidine inorganic pyrophosphate phosphatase	1.00	0.995	0.519
TAGLN	Transgelin	1.04	0.659	0.427
PCSK1	Neuroendocrine convertase 1	0.91	0.354	0.303
BMP1	Bone morphogenetic protein 1	1.07	0.331	0.292
GOLIM4	Golgi integral membrane protein 4	0.99	0.565	0.395
MAN1A1	Mannosyl-oligosaccharide 1,2-alpha-mannosidase IA	1.05	0.128	0.176
PGAM1	Phosphoglycerate mutase 1	1.11	0.002	0.011
SEMA4B	Semaphorin-4B	1.06	0.507	0.375
IL18BP	Interleukin-18-binding protein	1.06	0.215	0.232
MAN2B2	Epididymis-specific alpha-mannosidase	1.10	0.335	0.295
ST3GAL6	Type 2 lactosamine alpha-2,3-sialyltransferase	1.09	0.369	0.313
CD163	Scavenger receptor cysteine-rich type 1 protein M130	1.07	0.065	0.121
IGLON5	IgLON family member 5	1.01	0.790	0.465
CFD	Complement factor D	1.03	0.385	0.319
MDH2	Malate dehydrogenase, mitochondrial	1.23	0.025	0.070

PCDHGC5	Protocadherin gamma-C5	1.12	0.293	0.273
VIP	VIP peptides	1.61	0.388	0.320
MERTK	Tyrosine-protein kinase Mer	0.97	0.595	0.402
GM2A	Ganglioside GM2 activator	1.09	0.007	0.028
EFCAB14	EF-hand calcium-binding domain-containing protein 14	1.10	0.043	0.093
PVR	Poliovirus receptor	0.97	0.397	0.326
CA4	Carbonic anhydrase 4	0.97	0.732	0.446
HPRT1	Hypoxanthine-guanine phosphoribosyltransferase	1.36	0.004	0.019
REG1A	Lithostathine-1-alpha	0.95	0.778	0.462
LAMB2	Laminin subunit beta-2	0.91	0.006	0.026
VWC2	Brorin	0.97	0.597	0.403
PRRT3	Proline-rich transmembrane protein 3	1.00	0.908	0.494
RGMA	Repulsive guidance molecule A	1.02	0.696	0.440
CAT	Catalase	1.21	0.168	0.202
CGREF1	Cell growth regulator with EF hand domain protein 1	0.96	0.253	0.252
FUCA1	Tissue alpha-L-fucosidase	4.78	0.040	0.090
FUCA2	Plasma alpha-L-fucosidase	1.70	0.001	0.005
LCN2	Neutrophil gelatinase-associated lipocalin	0.96	0.542	0.389
PLA2G15	Group XV phospholipase A2	1.16	0.092	0.149
PCDH19	Protocadherin-19	1.00	0.964	0.511

MARCKS	Myristoylated alanine-rich C-kinase substrate	1.06	0.198	0.224
CCT4	T-complex protein 1 subunit delta	0.91	0.273	0.261
HS6ST3	Heparan-sulfate 6-O-sulfotransferase 3	0.81	0.000	0.001
ITIH3	Inter-alpha-trypsin inhibitor heavy chain H3	1.12	0.071	0.128
APOA1BP	NAD(P)H-hydrate epimerase	1.01	0.864	0.484
NFASC	Neurofascin	0.98	0.531	0.385
TPM2	Tropomyosin beta chain	1.09	0.545	0.389
SOD1	Superoxide dismutase [Cu-Zn]	0.96	0.020	0.058
CASC4	Protein CASC4	0.96	0.138	0.183
MYL6	Myosin light polypeptide 6	0.82	0.110	0.161
ANGPTL7	Angiopoietin-related protein 7	0.96	0.562	0.395
EPHA4	Ephrin type-A receptor 4	0.95	0.581	0.398
HAPLN4	Hyaluronan and proteoglycan link protein 4	0.97	0.728	0.445
ADAM10	Disintegrin and metalloproteinase domain-containing protein 10	1.07	0.099	0.153
TMEM132A	Transmembrane protein 132A	1.01	0.736	0.448
LAMB1	Laminin subunit beta-1	0.99	0.848	0.481
IGSF8	Immunoglobulin superfamily member 8	1.02	0.477	0.364
PPT1	Palmitoyl-protein thioesterase 1	0.99	0.868	0.484
EFNA1	Ephrin-A1	0.99	0.830	0.475
GPNMB	Transmembrane glycoprotein NMB	1.62	0.001	0.009
FETUB	Fetuin-B	0.85	0.153	0.194

PRSS8	Prostasin	1.03	0.760	0.455
HTRA1	Serine protease HTRA1	1.17	0.000	0.000
COL14A1	Collagen alpha-1(XIV) chain	1.00	0.958	0.509
PCDH8	Protocadherin-8	0.92	0.214	0.231
SNAP25	Synaptosomal-associated protein 25	0.87	0.108	0.161
ZMYM4	Zinc finger MYM-type protein 4	0.91	0.096	0.151
ATRN	Attractin	0.92	0.003	0.014
CA3	Carbonic anhydrase 3	1.00	0.991	0.519
FRRS1L	DOMON domain-containing protein FRRS1L	0.91	0.054	0.105
GALNT10	Polypeptide N-acetylgalactosaminyltransferase 10	1.48	0.024	0.068
B2M	Beta-2-microglobulin	1.05	0.380	0.317
CADM2	Cell adhesion molecule 2	0.89	0.138	0.183
GAS6	Growth arrest-specific protein 6	0.96	0.745	0.450
ISLR	Immunoglobulin superfamily containing leucine-rich repeat protein	1.12	0.007	0.027
NID1	Nidogen-1	1.06	0.133	0.178
CBLN3	Cerebellin-3	1.06	0.461	0.357
BTD	Biotinidase	0.93	0.398	0.326
C11orf87	Uncharacterized protein C11orf87	1.01	0.974	0.515
CLEC11A	C-type lectin domain family 11 member A	1.08	0.155	0.194
ECM2	Extracellular matrix protein 2	1.26	0.174	0.207
FBLN5	Fibulin-5	1.05	0.587	0.401

RPS6KA3	Ribosomal protein S6 kinase alpha-3	1.00	0.962	0.511
LPHN1	Latrophilin-1	0.94	0.201	0.225
SPARCL1	SPARC-like protein 1	0.98	0.244	0.249
PTHLH	Parathyroid hormone-related protein	0.82	0.044	0.093
TPI1	Triosephosphate isomerase	1.09	0.000	0.001
PLOD1	Procollagen-lysine,2-oxoglutarate 5-dioxygenase 1	1.34	0.057	0.110
IGFBP5	Insulin-like growth factor-binding protein 5	1.17	0.111	0.162
PTK7	Inactive tyrosine-protein kinase 7	0.83	0.017	0.054
CHST10	Carbohydrate sulfotransferase 10	1.11	0.066	0.123
GPR37	Prosaposin receptor GPR37	0.91	0.034	0.082
VCL	Vinculin	0.71	0.420	0.337
B4GAT1	Beta-1,4-glucuronyltransferase 1	1.01	0.867	0.484
CA2	Carbonic anhydrase 2	1.11	0.530	0.384
VNN1	Pantetheinase	0.76	0.199	0.224
NRP1	Neuropilin-1	0.98	0.438	0.346
CNTN4	Contactin-4	0.92	0.384	0.319
CDH2	Cadherin-2	0.95	0.070	0.127
CADM4	Cell adhesion molecule 4	0.97	0.024	0.068
KLK6	Kallikrein-6	0.94	0.013	0.045
SIAE	Sialate O-acetylesterase	1.02	0.625	0.413
VIM	Vimentin	0.86	0.168	0.202

SLC39A10	Zinc transporter ZIP10	1.06	0.135	0.180
SEZ6	Seizure protein 6 homolog	1.08	0.080	0.137
ADAMDEC 1	ADAM DEC1	0.95	0.564	0.395
CD44	CD44 antigen	1.11	0.001	0.006
EFNB2	Ephrin-B2	0.95	0.162	0.199
MIS12	Protein MIS12 homolog	0.79	0.036	0.083
PRDX1	Peroxiredoxin-1	1.04	0.134	0.179
GNAS	Neuroendocrine secretory protein 55	0.97	0.623	0.413
PLOD3	Procollagen-lysine,2-oxoglutarate 5-dioxygenase 3	1.03	0.598	0.403
PVRL2	Nectin-2	1.11	0.112	0.162
RNASET2	Ribonuclease T2	1.13	0.000	0.001
SIL1	Nucleotide exchange factor SIL1	1.05	0.209	0.231
USP42	Ubiquitin carboxyl-terminal hydrolase 42	0.98	0.856	0.481
TNFRSF21	Tumor necrosis factor receptor superfamily member 21	0.90	0.043	0.093
SNCG	Gamma-synuclein	1.10	0.017	0.052
GSTP1	Glutathione S-transferase P	1.25	0.039	0.089
IDS	Iduronate 2-sulfatase	1.02	0.436	0.345
SELL	L-selectin	0.98	0.498	0.371
EPHA10	Ephrin type-A receptor 10	0.89	0.008	0.031
IMPAD1	Inositol monophosphatase 3	1.07	0.446	0.349

MASP2	Mannan-binding lectin serine protease 2	0.94	0.617	0.412
GPATCH2	G patch domain-containing protein 2	0.99	0.836	0.476
ART3	Ecto-ADP-ribosyltransferase 3	1.02	0.292	0.273
SUMF2	Sulfatase-modifying factor 2	0.96	0.494	0.370
COL15A1	Collagen alpha-1(XV) chain	0.90	0.238	0.248
RNF13	E3 ubiquitin-protein ligase RNF13	0.90	0.062	0.116
VMO1	Vitelline membrane outer layer protein 1 homolog	0.97	0.731	0.446
MSN	Moesin	1.05	0.399	0.327
HRG	Histidine-rich glycoprotein	0.59	0.000	0.000
APP	Amyloid beta A4 protein	1.00	0.850	0.481
FCN3	Ficolin-3	0.58	0.037	0.085
SERPINA10	Protein Z-dependent protease inhibitor	1.93	0.053	0.104
PRDX2	Peroxiredoxin-2	1.18	0.249	0.250
RAB22A	Ras-related protein Rab-22A	0.87	0.052	0.104
TALDO1	Transaldolase	1.05	0.334	0.295
DLG5	Disks large homolog 5	0.97	0.692	0.440
DSC1	Desmocollin-1	0.78	0.000	0.001
PALM	Paralemmin-1	0.90	0.177	0.208
GGH	Gamma-glutamyl hydrolase	0.94	0.186	0.216
ABCA2	ATP-binding cassette sub-family A member 2	0.99	0.709	0.441
SORL1	Sortilin-related receptor	1.06	0.433	0.344

APOA2	Apolipoprotein A-II	0.96	0.454	0.353
TIMP1	Metalloproteinase inhibitor 1	1.01	0.866	0.484
CEMIP	Cell migration-inducing and hyaluronan-binding protein	1.03	0.461	0.357
GRIA4	Glutamate receptor 4	1.15	0.225	0.239
TAGLN3	Transgelin-3	0.48	0.075	0.131
FAIM2	Protein lifeguard 2	0.99	0.852	0.481
ENPP5	Ectonucleotide pyrophosphatase/phosphodiesterase family member 5	0.79	0.057	0.110
GPLD1	Phosphatidylinositol-glycan-specific phospholipase D	0.85	0.011	0.039
CALCA	Calcitonin	0.88	0.273	0.261
BCAM	Basal cell adhesion molecule	0.93	0.191	0.219
NEDD8	NEDD8	1.05	0.229	0.242
CNTN6	Contactin-6	0.97	0.359	0.306
PTPRD	Receptor-type tyrosine-protein phosphatase delta	0.98	0.316	0.286
LRG1	Leucine-rich alpha-2-glycoprotein	1.07	0.026	0.071
HSPA5	78 kDa glucose-regulated protein	0.99	0.704	0.441
KIAA0319	Dyslexia-associated protein KIAA0319	0.94	0.147	0.188
KCNE5	Potassium voltage-gated channel subfamily E regulatory beta subunit 5	1.00	0.977	0.515
CD99L2	CD99 antigen-like protein 2	0.93	0.014	0.047
CTSZ	Cathepsin Z	0.95	0.074	0.131

LRRC4	Leucine-rich repeat-containing protein 4	0.92	0.605	0.406
SERPINI1	Neuroserpin	0.92	0.000	0.004
SOD2	Superoxide dismutase [Mn], mitochondrial	1.02	0.482	0.365
HGFAC	Hepatocyte growth factor activator	0.94	0.444	0.348
CAMK2D	Calcium/calmodulin-dependent protein kinase type II subunit delta	0.91	0.256	0.253
NENF	Neudesin	0.97	0.270	0.260
SLC1A2	Excitatory amino acid transporter 2	1.32	0.124	0.171
FABP5	Fatty acid-binding protein, epidermal	1.08	0.186	0.216
DDR1	Epithelial discoidin domain-containing receptor 1	1.15	0.132	0.178
LYVE1	Lymphatic vessel endothelial hyaluronic acid receptor 1	0.97	0.512	0.377
KRT77	Keratin, type II cytoskeletal 1b	3.51	0.287	0.270
B3GNT2	N-acetyllactosaminide beta-1,3-N-acetylglucosaminyltransferase 2	1.10	0.009	0.034
LIPA	Lysosomal acid lipase/cholesteryl ester hydrolase	0.77	0.035	0.082
LTBP2	Latent-transforming growth factor beta-binding protein 2	1.14	0.249	0.250
NPC2	Epididymal secretory protein E1	1.03	0.090	0.148
WSCD1	WSC domain-containing protein 1	1.10	0.148	0.188
CLCN6	Chloride transport protein 6	0.87	0.178	0.209
NDRG2	Protein NDRG2	1.15	0.148	0.188
F11	Coagulation factor XI	0.93	0.602	0.405
KLKB1	Plasma kallikrein	1.01	0.918	0.496

MINPP1	Multiple inositol polyphosphate phosphatase 1	1.06	0.233	0.245
PGLYRP2	N-acetylmuramoyl-L-alanine amidase	1.08	0.182	0.213
HRSP12	Ribonuclease UK114	0.96	0.570	0.395
NLGN1	Neuroligin-1	1.22	0.436	0.345
COL12A1	Collagen alpha-1(XII) chain	0.97	0.702	0.441
TBCA	Tubulin-specific chaperone A	1.05	0.029	0.074
CCDC13	Coiled-coil domain-containing protein 13	1.21	0.801	0.466
EDN3	Endothelin-3	0.87	0.248	0.250
EPHA5	Ephrin type-A receptor 5	1.02	0.633	0.417
FKBP1A	Peptidyl-prolyl cis-trans isomerase FKBP1A	0.83	0.130	0.176
RNASE4	Ribonuclease 4	0.97	0.742	0.449
WBP2	WW domain-binding protein 2	0.81	0.104	0.158
SERPING1	Plasma protease C1 inhibitor	0.93	0.273	0.261
C1RL	Complement C1r subcomponent-like protein	0.95	0.467	0.360
GALNT2	Polypeptide N-acetylgalactosaminyltransferase 2	0.96	0.720	0.444
PTPRK	Receptor-type tyrosine-protein phosphatase kappa	0.99	0.875	0.485
NRXN2	Neurexin-2-beta	1.16	0.003	0.014
TANGO6	Transport and Golgi organization protein 6 homolog	0.90	0.259	0.254
GPR180	Integral membrane protein GPR180	0.90	0.143	0.186
NPTX1	Neuronal pentraxin-1	0.90	0.319	0.287

PODXL2	Podocalyxin-like protein 2	1.05	0.402	0.328
PLD4	Phospholipase D4	1.10	0.594	0.402
CLU	Clusterin	1.14	0.000	0.000
DNASE2	Deoxyribonuclease-2-alpha	0.93	0.244	0.249
PTPRF	Receptor-type tyrosine-protein phosphatase F	0.84	0.031	0.077
C1QTNF3	Complement C1q tumor necrosis factor-related protein 3	1.26	0.001	0.005
C14orf37	Uncharacterized protein C14orf37	0.92	0.214	0.231
METRN	Meteorin	1.25	0.000	0.002
MRC2	C-type mannose receptor 2	0.95	0.159	0.196
DPP10	Inactive dipeptidyl peptidase 10	1.19	0.088	0.146
FGFR2	Fibroblast growth factor receptor 2	0.97	0.355	0.303
PCDH18	Protocadherin-18	0.99	0.887	0.489
PVRL1	Nectin-1	0.93	0.044	0.093
ADAM23	Disintegrin and metalloproteinase domain-containing protein 23	0.99	0.897	0.493
FAM3A	Protein FAM3A	1.00	0.968	0.512
FBLN2	Fibulin-2	1.13	0.035	0.082
FGA	Fibrinogen alpha chain	0.83	0.001	0.005
MENT	Protein MENT	0.95	0.923	0.498
TFRC	Transferrin receptor protein 1	0.88	0.092	0.149
BST1	ADP-ribosyl cyclase/cyclic ADP-ribose hydrolase 2	1.21	0.002	0.011
SPOCK2	Testican-2	1.04	0.483	0.365

TWSG1	Twisted gastrulation protein homolog 1	1.03	0.372	0.314
ATP6AP1	V-type proton ATPase subunit S1	1.16	0.254	0.252
C1S	Complement C1s subcomponent	0.97	0.228	0.242
GFAP	Glial fibrillary acidic protein	1.75	0.001	0.006
IGFBP1	Insulin-like growth factor-binding protein 1	0.57	0.306	0.279
LPHN3	Latrophilin-3	0.95	0.017	0.054
LTBP4	Latent-transforming growth factor beta-binding protein 4	0.96	0.403	0.328
MFAP4	Microfibril-associated glycoprotein 4	0.65	0.008	0.031
TRMT2A	tRNA (uracil-5-)-methyltransferase homolog A	1.02	0.859	0.482
XXYLT1	Xyloside xylosyltransferase 1	1.02	0.758	0.455
APOL1	Apolipoprotein L1	0.38	0.251	0.251
C1QTNF4	Complement C1q tumor necrosis factor-related protein 4	1.04	0.652	0.424
CA11	Carbonic anhydrase-related protein 11	0.95	0.543	0.389
COMP	Cartilage oligomeric matrix protein	0.78	0.008	0.032
EFNB1	Ephrin-B1	0.98	0.619	0.412
FAM196A	Protein FAM196A	1.03	0.800	0.466
ATP1B2	Sodium/potassium-transporting ATPase subunit beta-2	1.05	0.341	0.298
CALY	Neuron-specific vesicular protein calcyon	0.95	0.201	0.225
ESAM	Endothelial cell-selective adhesion molecule	0.91	0.066	0.123
GALNT7	N-acetylgalactosaminyltransferase 7	1.09	0.264	0.256

MRC1	Macrophage mannose receptor 1	1.05	0.508	0.375
SPRN	Shadow of prion protein	1.00	0.991	0.519
TMEM132D	Transmembrane protein 132D	0.62	0.058	0.111
ABI3BP	Target of Nesh-SH3	0.97	0.569	0.395
GABARAP L2	Gamma-aminobutyric acid receptor-associated protein-like 2	0.83	0.171	0.204
LOX	Protein-lysine 6-oxidase	1.21	0.620	0.412
SGCE	Epsilon-sarcoglycan	1.07	0.095	0.151
CD33	Myeloid cell surface antigen CD33	0.97	0.752	0.453
IFNAR1	Interferon alpha/beta receptor 1	0.86	0.709	0.441
PKM	Pyruvate kinase PKM	1.64	0.121	0.168
PLTP	Phospholipid transfer protein	1.18	0.009	0.033
TXNDC17	Thioredoxin domain-containing protein 17	1.28	0.001	0.008
DPYSL2	Dihydropyrimidinase-related protein 2	1.12	0.117	0.165
FAM174A	Membrane protein FAM174A	0.89	0.023	0.065
BPGM	Bisphosphoglycerate mutase	0.71	0.595	0.402
CECR1	Adenosine deaminase CECR1	1.14	0.040	0.089
THBS1	Thrombospondin-1	1.42	0.117	0.165
TYRO3	Tyrosine-protein kinase receptor TYRO3	0.94	0.015	0.050
CDH10	Cadherin-10	1.04	0.660	0.427
CTSO	Cathepsin O	0.62	0.008	0.033
ACYP1	Acylphosphatase-1	1.15	0.205	0.228

APOB	Apolipoprotein B-100	1.21	0.035	0.082
C1R	Complement C1r subcomponent	0.98	0.263	0.256
CTHRC1	Collagen triple helix repeat-containing protein 1	0.91	0.243	0.249
KLK11	Kallikrein-11	1.02	0.805	0.467
RCN1	Reticulocalbin-1	0.97	0.235	0.247
S100A8	Protein S100-A8	1.02	0.712	0.442
SELM	Selenoprotein M	0.96	0.418	0.336
ACAN	Aggrecan core protein	0.89	0.544	0.389
CNDP1	Beta-Ala-His dipeptidase	1.06	0.018	0.054
RTBDN	Retbindin	1.03	0.711	0.441
LRRC4B	Leucine-rich repeat-containing protein 4B	0.96	0.620	0.412
SAA4	Serum amyloid A-4 protein	0.91	0.243	0.249
HEG1	Protein HEG homolog 1	1.03	0.593	0.402
CUTA	Protein CutA	1.02	0.583	0.399
FAM198A	Protein FAM198A	1.35	0.164	0.199
SPP1	Osteopontin	1.02	0.686	0.438
PCDH7	Protocadherin-7	0.88	0.003	0.016
NRP2	Neuropilin-2	0.99	0.657	0.427
PI16	Peptidase inhibitor 16	0.63	0.000	0.000
SFRP4	Secreted frizzled-related protein 4	0.94	0.637	0.418
CYTL1	Cytokine-like protein 1	0.98	0.564	0.395

LRRN1	Leucine-rich repeat neuronal protein 1	1.20	0.005	0.022
MAN1B1	Endoplasmic reticulum mannosyl-oligosaccharide 1,2-alpha-mannosidase	1.14	0.140	0.183
POSTN	Periostin	1.69	0.032	0.079
CORO1A	Coronin-1A	1.11	0.240	0.249
OLFML3	Olfactomedin-like protein 3	1.09	0.440	0.346
PENK	Proenkephalin-A	0.91	0.121	0.168
RNASE6	Ribonuclease K6	0.98	0.693	0.440
SAA2	Serum amyloid A-2 protein	2.42	0.115	0.164
B3GNT9	UDP-GlcNAc:betaGal beta-1,3-N-acetylglucosaminyltransferase 9	1.09	0.284	0.269
CSF1	Macrophage colony-stimulating factor 1	1.08	0.257	0.254
DYNLL2	Dynein light chain 2, cytoplasmic	1.08	0.243	0.249
GRB2	Growth factor receptor-bound protein 2	1.06	0.156	0.194
PITHD1	PITH domain-containing protein 1	1.12	0.009	0.034
ADAM9	Disintegrin and metalloproteinase domain-containing protein 9	1.05	0.351	0.303
BAI1	Brain-specific angiogenesis inhibitor 1	1.20	0.372	0.314
CPQ	Carboxypeptidase Q	1.04	0.480	0.364
C17orf47	Uncharacterized protein C17orf47	0.95	0.516	0.380
DSC2	Desmocollin-2	0.91	0.026	0.071
INADL	InaD-like protein	0.94	0.525	0.383
HIST1H4A	Histone H4	1.02	0.893	0.491

RELT	Tumor necrosis factor receptor superfamily member 19L	1.00	0.981	0.516
LAMA2	Laminin subunit alpha-2	0.97	0.241	0.249
AIFM1	Apoptosis-inducing factor 1, mitochondrial	0.93	0.305	0.279
TM9SF3	Transmembrane 9 superfamily member 3	0.96	0.811	0.469
EPHA6	Ephrin type-A receptor 6	1.12	0.145	0.187
PTMA	Prothymosin alpha	0.20	0.061	0.115
SPON2	Spondin-2	0.92	0.191	0.219
SYT1	Synaptotagmin-1	0.83	0.249	0.250
SORCS1	VPS10 domain-containing receptor SorCS1	1.11	0.221	0.236
CPD	Carboxypeptidase D	0.93	0.275	0.263
SEMA3G	Semaphorin-3G	1.06	0.365	0.310
ADAM11	Disintegrin and metalloproteinase domain-containing protein 11	0.92	0.217	0.233
CLIC1	Chloride intracellular channel protein 1	2.74	0.024	0.069
VCAN	Versican core protein	1.01	0.852	0.481
EPDR1	Mammalian ependymin-related protein 1	0.90	0.184	0.215
FCGR3A	Low affinity immunoglobulin gamma Fc region receptor III-A	1.09	0.051	0.102
FCGBP	IgGFc-binding protein	0.82	0.039	0.088
FSTL5	Follistatin-related protein 5	1.02	0.885	0.489
HSPA4	Heat shock 70 kDa protein 4	1.01	0.871	0.484
LAMA4	Laminin subunit alpha-4	1.10	0.242	0.249

MAN1A2	Mannosyl-oligosaccharide 1,2-alpha-mannosidase IB	1.17	0.022	0.063
MIA3	Melanoma inhibitory activity protein 3	0.99	0.788	0.465
PRG4	Proteoglycan 4	1.21	0.610	0.409
PRSS23	Serine protease 23	1.01	0.977	0.515
TMEM130	Transmembrane protein 130	0.92	0.544	0.389
AFP	Alpha-fetoprotein	1.28	0.130	0.176
PLXNB3	Plexin-B3	1.09	0.211	0.231
FGFR1	Fibroblast growth factor receptor 1	1.05	0.291	0.272
SOD3	Extracellular superoxide dismutase [Cu-Zn]	0.87	0.006	0.025
VWF	von Willebrand factor	0.95	0.451	0.352
GOLM1	Golgi membrane protein 1	1.08	0.177	0.208
EXTL2	Exostosin-like 2	1.02	0.841	0.479
SEMA6A	Semaphorin-6A	1.23	0.236	0.248
KRT16	Keratin, type I cytoskeletal 16	0.39	0.021	0.061
OSCAR	Osteoclast-associated immunoglobulin-like receptor	0.90	0.298	0.277
CDH6	Cadherin-6	0.96	0.034	0.081
TMEM132E	Transmembrane protein 132E	1.78	0.581	0.398
CTSH	Pro-cathepsin H	0.85	0.002	0.011
MCFD2	Multiple coagulation factor deficiency protein 2	1.63	0.000	0.000
APOD	Apolipoprotein D	0.96	0.283	0.268
HSPE1	10 kDa heat shock protein, mitochondrial	1.05	0.163	0.199

CEP135	Centrosomal protein of 135 kDa	0.89	0.397	0.326
NPPC	C-type natriuretic peptide	1.01	0.882	0.488
ASPH	Aspartyl/asparaginyl beta-hydroxylase	0.88	0.165	0.199
CDH5	Cadherin-5	0.99	0.903	0.494
CALR	Calreticulin	0.95	0.351	0.303
CANX	Calnexin	0.93	0.377	0.317
CBLN2	Cerebellin-2	1.85	0.386	0.319
COL3A1	Collagen alpha-1(III) chain	0.99	0.738	0.448
COL6A2	Collagen alpha-2(VI) chain	1.16	0.009	0.034
EFNB3	Ephrin-B3	0.98	0.728	0.445
FAM20C	Extracellular serine/threonine protein kinase FAM20C	1.02	0.636	0.418
FGFR3	Fibroblast growth factor receptor 3	0.91	0.049	0.100
HAVCR2	Hepatitis A virus cellular receptor 2	1.19	0.001	0.008
IGF2	Insulin-like growth factor II	0.98	0.695	0.440
MANSC1	MANSC domain-containing protein 1	0.55	0.280	0.266
MGP	Matrix Gla protein	1.01	0.923	0.498
MYRF	Myelin regulatory factor	1.22	0.692	0.440
PDGFRB	Platelet-derived growth factor receptor beta	1.02	0.871	0.484
HNRNPA2 B1	Heterogeneous nuclear ribonucleoproteins A2/B1	1.04	0.946	0.507
ROBO2	Roundabout homolog 2	1.00	0.993	0.519
SLITRK4	SLIT and NTRK-like protein 4	1.26	0.097	0.151

UCHL1	Ubiquitin carboxyl-terminal hydrolase isozyme L1	1.34	0.000	0.000
VWA1	von Willebrand factor A domain-containing protein 1	1.05	0.464	0.358
WFDC1	WAP four-disulfide core domain protein 1	0.88	0.248	0.250
CTSL	Cathepsin L1	0.86	0.000	0.000
GLDN	Gliomedin	1.13	0.110	0.161
SORCS3	VPS10 domain-containing receptor SorCS3	1.52	0.002	0.011
CFHR2	Complement factor H-related protein 2	1.27	0.211	0.231
FLNA	Filamin-A	0.98	0.914	0.495
GPI	Glucose-6-phosphate isomerase	1.17	0.027	0.071
VSTM2A	V-set and transmembrane domain-containing protein 2A	0.83	0.026	0.070
F10	Coagulation factor X	0.44	0.470	0.361
ACYP2	Acylphosphatase-2	0.96	0.123	0.170
PARK7	Protein deglycase DJ-1	0.94	0.550	0.391
ROBO4	Roundabout homolog 4	0.88	0.222	0.237
SNED1	Sushi, nidogen and EGF-like domain-containing protein 1	1.02	0.579	0.398
QSOX2	Sulfhydryl oxidase 2	1.36	0.326	0.290
ADGRB2	Adhesion G protein-coupled receptor B2	0.98	0.523	0.383
CRISPLD1	Cysteine-rich secretory protein LCCL domain-containing 1	1.14	0.067	0.123
EMILIN2	EMILIN-2	1.04	0.442	0.347
RARRES2	Retinoic acid receptor responder protein 2	1.16	0.350	0.303

DDB1	DNA damage-binding protein 1	1.06	0.542	0.389
CDH11	Cadherin-11	0.89	0.439	0.346
SMPDL3B	Acid sphingomyelinase-like phosphodiesterase 3b	1.17	0.008	0.032
CD109	CD109 antigen	1.35	0.089	0.146
LAMP1	Lysosome-associated membrane glycoprotein 1	0.97	0.320	0.287
PLXDC2	Plexin domain-containing protein 2	1.13	0.129	0.176
RNASE1	Ribonuclease pancreatic	1.24	0.739	0.448
CDH22	Cadherin-22	0.94	0.852	0.481
GOT2	Aspartate aminotransferase, mitochondrial	1.32	0.001	0.007
KRT10	Keratin, type I cytoskeletal 10	0.83	0.154	0.194
NPY	Pro-neuropeptide Y	0.95	0.386	0.319
MATN2	Matrilin-2	1.05	0.491	0.369
GMFB	Glia maturation factor beta	1.00	0.955	0.509
HBD	Hemoglobin subunit delta	1.33	0.175	0.208
EEF1G	Elongation factor 1-gamma	1.02	0.861	0.483
ARHGDIB	Rho GDP-dissociation inhibitor 2	1.36	0.511	0.377
LBP	Lipopolysaccharide-binding protein	1.19	0.133	0.178
MDGA1	MAM domain-containing glycosylphosphatidylinositol anchor protein 1	1.09	0.715	0.442
NUDT5	ADP-sugar pyrophosphatase	2.17	0.053	0.105
PTGDS	Prostaglandin-H2 D-isomerase	0.91	0.041	0.090

TNC	Tenascin	1.38	0.301	0.277
LRP1B	Low-density lipoprotein receptor-related protein 1B	1.08	0.739	0.448
SIRPA	Tyrosine-protein phosphatase non-receptor type substrate 1	0.83	0.014	0.046
SERPINA3	Alpha-1-antichymotrypsin	1.15	0.000	0.000
NEFH	Neurofilament heavy polypeptide	2.76	0.097	0.151
PCDHGC3	Protocadherin gamma-C3	1.12	0.789	0.465
CNTNAP4	Contactin-associated protein-like 4	1.16	0.109	0.161
UNC5C	Netrin receptor UNC5C	0.98	0.682	0.437
CPSF1	Cleavage and polyadenylation specificity factor subunit 1	0.30	0.035	0.082
CASP14	Caspase-14	1.17	0.854	0.481
CCK	Cholecystokinin	0.83	0.016	0.052
HSPA2	Heat shock-related 70 kDa protein 2	1.07	0.238	0.248
ICOSLG	ICOS ligand	0.95	0.800	0.466
KIAA1549L	UPF0606 protein KIAA1549L	0.69	0.463	0.357
P4HB	Protein disulfide-isomerase	0.79	0.000	0.004
PON1	Serum paraoxonase/arylesterase 1	0.78	0.000	0.000
TMEM132C	Transmembrane protein 132C	0.97	0.903	0.494
CHAD	Chondroadherin	0.97	0.866	0.484
CRYM	Ketimine reductase mu-crystallin	0.74	0.500	0.372
GAA	Lysosomal alpha-glucosidase	1.33	0.041	0.090
PRNP	Major prion protein	0.82	0.000	0.003

SBSN	Suprabasin	1.03	0.808	0.468
SPOCK3	Testican-3	0.96	0.241	0.249
VSIG4	V-set and immunoglobulin domain-containing protein 4	0.89	0.004	0.019
CD177	CD177 antigen	2.08	0.023	0.065
APOH	Beta-2-glycoprotein 1	0.88	0.313	0.284
LTBP1	Latent-transforming growth factor beta-binding protein 1	0.98	0.822	0.474
IGF2R	Cation-independent mannose-6-phosphate receptor	1.09	0.533	0.385
SEMA3F	Semaphorin-3F	1.24	0.247	0.250
SPOCK1	Testican-1	0.96	0.352	0.303
IL1R2	Interleukin-1 receptor type 2	1.93	0.309	0.281
LSR	Lipolysis-stimulated lipoprotein receptor	0.92	0.003	0.014
ADAMTS8	A disintegrin and metalloproteinase with thrombospondin motifs 8	1.09	0.685	0.438
CDH12	Cadherin-12	0.72	0.017	0.052
COL5A1	Collagen alpha-1(V) chain	0.88	0.120	0.168
CPN2	Carboxypeptidase N subunit 2	0.96	0.678	0.435
SERPINE2	Glia-derived nexin	0.44	0.071	0.127
NBL1	Neuroblastoma suppressor of tumorigenicity 1	0.93	0.112	0.163
PTPRM	Receptor-type tyrosine-protein phosphatase mu	0.96	0.568	0.395
SCN3B	Sodium channel subunit beta-3	0.90	0.031	0.077

SEMA3C	Semaphorin-3C	1.03	0.904	0.494
COCH	Cochlin	1.01	0.919	0.496
ACP2	Lysosomal acid phosphatase	2.60	0.243	0.249
NCSTN	Nicastrin	1.32	0.164	0.199
PPBP	Platelet basic protein	1.61	0.073	0.130
YWHAB	14-3-3 protein beta/alpha	1.09	0.149	0.189
SLC6A1	Sodium- and chloride-dependent GABA transporter 1	1.05	0.434	0.345
IMPA1	Inositol monophosphatase 1	1.20	0.062	0.117
APOC1	Apolipoprotein C-I	1.63	0.021	0.061
FGL2	Fibroleukin	0.95	0.454	0.353
FABP3	Fatty acid-binding protein, heart	0.92	0.542	0.389
ANPEP	Aminopeptidase N	0.84	0.139	0.183
C12orf49	UPF0454 protein C12orf49	1.20	0.000	0.004
DBH	Dopamine beta-hydroxylase	1.13	0.800	0.466
NUP153	Nuclear pore complex protein Nup153	1.08	0.564	0.395
BSG	Basigin	0.94	0.034	0.081
B3GAT3	Galactosylgalactosylxylosylprotein 3-beta-glucuronosyltransferase 3	1.19	0.015	0.049
EPHA7	Ephrin type-A receptor 7	0.98	0.494	0.370
FAM20B	Glycosaminoglycan xylosylkinase	1.13	0.001	0.006
AEBP1	Adipocyte enhancer-binding protein 1	0.89	0.477	0.364
MAN1C1	Mannosyl-oligosaccharide 1,2-alpha-mannosidase IC	1.09	0.034	0.081

MGAT2	Alpha-1,6-mannosyl-glycoprotein 2-beta-N-acetylglucosaminyltransferase	2.21	0.035	0.082
SH3BGRL	SH3 domain-binding glutamic acid-rich-like protein	1.02	0.551	0.391
APLP2	Amyloid-like protein 2	1.01	0.773	0.459
ADAM17	Disintegrin and metalloproteinase domain-containing protein 17	1.05	0.694	0.440
B4GALNT4	N-acetyl-beta-glucosaminyl-glycoprotein 4-beta-N-acetylgalactosaminyltransferase 1	0.98	0.793	0.465
PEBP4	Phosphatidylethanolamine-binding protein 4	1.13	0.156	0.194
VAMP2	Vesicle-associated membrane protein 2	1.65	0.307	0.280
PGK1	Phosphoglycerate kinase 1	1.23	0.117	0.165
ANTXR1	Anthrax toxin receptor 1	1.30	0.351	0.303
LGALS3	Galectin-3	1.12	0.102	0.156
CHRD	Chordin	1.14	0.061	0.115
GLO1	Lactoylglutathione lyase	1.15	0.095	0.151
FBLN1	Fibulin-1	1.01	0.649	0.423
GSR	Glutathione reductase, mitochondrial	0.73	0.170	0.203
PON3	Serum paraoxonase/lactonase 3	0.78	0.092	0.149
PROCR	Endothelial protein C receptor	1.05	0.783	0.464
CFHR3	Complement factor H-related protein 3	1.14	0.187	0.216
PTPRJ	Receptor-type tyrosine-protein phosphatase eta	1.05	0.416	0.335
SHBG	Sex hormone-binding globulin	0.75	0.001	0.007

POLR3F	DNA-directed RNA polymerase III subunit RPC6	0.96	0.573	0.396
KRT5	Keratin, type II cytoskeletal 5	0.81	0.390	0.321
MXRA8	Matrix-remodeling-associated protein 8	1.12	0.000	0.002
THBS2	Thrombospondin-2	0.97	0.630	0.416
NTNG1	Netrin-G1	0.91	0.688	0.439
ECM1	Extracellular matrix protein 1	1.00	0.953	0.509
GLOD4	Glyoxalase domain-containing protein 4	1.06	0.238	0.248
PLD3	Phospholipase D3	1.07	0.644	0.421
PFN1	Profilin-1	1.32	0.016	0.052
SLC38A10	Putative sodium-coupled neutral amino acid transporter 10	1.09	0.009	0.034
FCGR2A	Low affinity immunoglobulin gamma Fc region receptor II-a	0.95	0.096	0.151
PSAT1	Phosphoserine aminotransferase	1.06	0.223	0.238
CTSA	Lysosomal protective protein	1.04	0.849	0.481
C6orf201	Uncharacterized protein C6orf201	0.96	0.560	0.395
DPP6	Dipeptidyl aminopeptidase-like protein 6	1.09	0.330	0.292
ENPP4	Bis(5'-adenosyl)-triphosphatase ENPP4	1.06	0.312	0.283
PRL	Prolactin	0.59	0.364	0.310
SEMA6D	Semaphorin-6D	1.06	0.269	0.260
CD84	SLAM family member 5	1.02	0.949	0.508
MMRN1	Multimerin-1	1.08	0.302	0.277
AJAP1	Adherens junction-associated protein 1	0.93	0.229	0.242

GPC1	Glypican-1	3.43	0.001	0.007
JAM3	Junctional adhesion molecule C	0.94	0.248	0.250
PCDH9	Protocadherin-9	0.88	0.071	0.127
PVALB	Parvalbumin alpha	1.05	0.673	0.433
AMPH	Amphiphysin	0.97	0.762	0.456
BACE1	Beta-secretase 1	1.04	0.754	0.453
IGFBPL1	Insulin-like growth factor-binding protein-like 1	0.98	0.878	0.486
LFNG	Beta-1,3-N-acetylglucosaminyltransferase lunatic fringe	2.12	0.110	0.161
PRDX6	Peroxiredoxin-6	1.25	0.412	0.333
TLN2	Talin-2	1.15	0.147	0.188
ENDOD1	Endonuclease domain-containing 1 protein	0.96	0.313	0.284
HEPACAM	Hepatocyte cell adhesion molecule	0.99	0.951	0.509
GAPDH	Glyceraldehyde-3-phosphate dehydrogenase	1.64	0.029	0.076
C5	Complement C5	1.13	0.412	0.333
EFEMP2	EGF-containing fibulin-like extracellular matrix protein 2	0.92	0.040	0.090
SEPP1	Selenoprotein P	1.15	0.116	0.165
COL5A2	Collagen alpha-2(V) chain	0.81	0.044	0.093
ARRDC2	Arrestin domain-containing protein 2	1.10	0.617	0.412
RCN2	Reticulocalbin-2	1.10	0.543	0.389
CAPG	Macrophage-capping protein	1.18	0.006	0.026

MSTN	Growth/differentiation factor 8	1.00	0.986	0.517
LRP1	Prolow-density lipoprotein receptor-related protein 1	0.91	0.015	0.049
NSG1	Neuron-specific protein family member 1	0.91	0.244	0.249
PDGFA	Platelet-derived growth factor subunit A	1.00	0.902	0.494
HEXB	Beta-hexosaminidase subunit beta	1.11	0.185	0.215
LAMC1	Laminin subunit gamma-1	0.97	0.119	0.168
ERAP1	Endoplasmic reticulum aminopeptidase 1	0.90	0.082	0.140
BLVRB	Flavin reductase (NADPH)	1.45	0.164	0.199
CALB1	Calbindin	1.05	0.529	0.384
ENO2	Gamma-enolase	0.97	0.765	0.456
FAH	Fumarylacetoacetase	0.84	0.104	0.158
MANEAL	Glycoprotein endo-alpha-1,2-mannosidase-like protein	1.08	0.106	0.159
VNN3	Vascular non-inflammatory molecule 3	0.92	0.790	0.465
ABHD14A	Alpha/beta hydrolase domain-containing protein 14A	1.06	0.414	0.335
COL11A1	Collagen alpha-1(XI) chain	0.96	0.304	0.279
DSP	Desmoplakin	0.87	0.077	0.134
PRDX3	Thioredoxin-dependent peroxide reductase, mitochondrial	1.21	0.019	0.057
ACE	Angiotensin-converting enzyme	0.97	0.709	0.441
CDH18	Cadherin-18	0.93	0.642	0.420
CELSR2	Cadherin EGF LAG seven-pass G-type receptor 2	1.16	0.373	0.314

CHST15	Carbohydrate sulfotransferase 15	1.20	0.001	0.008
FGB	Fibrinogen beta chain	0.80	0.001	0.009
FGG	Fibrinogen gamma chain	0.75	0.000	0.001
LAYN	Layilin	0.88	0.047	0.098
NTRK2	BDNF/NT-3 growth factors receptor	0.89	0.526	0.383
SULF2	Extracellular sulfatase Sulf-2	1.03	0.319	0.287
NPDC1	Neural proliferation differentiation and control protein 1	0.45	0.010	0.036
PTPRU	Receptor-type tyrosine-protein phosphatase U	1.08	0.725	0.445
LYZ	Lysozyme C	1.32	0.000	0.000
LDHB	L-lactate dehydrogenase B chain	1.52	0.000	0.000
CLN5	Ceroid-lipofuscinosis neuronal protein 5	0.98	0.698	0.440
FKBP4	Peptidyl-prolyl cis-trans isomerase FKBP4	0.74	0.085	0.143
CHST8	Carbohydrate sulfotransferase 8	1.55	0.032	0.079
ICAM2	Intercellular adhesion molecule 2	0.98	0.698	0.440
PDIA6	Protein disulfide-isomerase A6	1.04	0.352	0.303
TKT	Transketolase	1.21	0.084	0.141
VTN	Vitronectin	1.28	0.055	0.107
DBI	Acyl-CoA-binding protein	1.04	0.247	0.250
PEBP1	Phosphatidylethanolamine-binding protein 1	1.10	0.176	0.208
BOC	Brother of CDO	0.98	0.795	0.465
SHISA5	Protein shisa-5	1.07	0.855	0.481

COLEC12	Collectin-12	0.96	0.026	0.071
VASN	Vasorin	1.09	0.014	0.048
ALCAM	CD166 antigen	0.89	0.001	0.008
GALNT18	Polypeptide N-acetylgalactosaminyltransferase 18	1.25	0.002	0.011
LRP11	Low-density lipoprotein receptor-related protein 11	0.95	0.787	0.465
TRIL	TLR4 interactor with leucine rich repeats	0.85	0.087	0.145
ALB	Serum albumin	0.70	0.154	0.194
CD248	Endosialin	0.99	0.788	0.465
DDAH1	N(G),N(G)-dimethylarginine dimethylaminohydrolase 1	1.00	0.967	0.512
IGFBP3	Insulin-like growth factor-binding protein 3	0.99	0.954	0.509
LMAN1	Protein ERGIC-53	1.11	0.002	0.012
BCAN	Brevican core protein	1.03	0.104	0.158
ZFPL1	Zinc finger protein-like 1	1.06	0.692	0.440
RNASE2	Non-secretory ribonuclease	1.06	0.425	0.340
RNF123	E3 ubiquitin-protein ligase RNF123	0.89	0.052	0.103
C8G	Complement component C8 gamma chain	0.97	0.805	0.467
CSF2RA	Granulocyte-macrophage colony-stimulating factor receptor subunit alpha	0.95	0.565	0.395
ARHGDIA	Rho GDP-dissociation inhibitor 1	1.04	0.592	0.402
UNC5D	Netrin receptor UNC5D	0.68	0.003	0.017
BCHE	Cholinesterase	0.91	0.087	0.145

CNTN2	Contactin-2	1.08	0.002	0.011
GPR37L1	Prosaposin receptor GPR37L1	1.27	0.826	0.474
IGSF1	Immunoglobulin superfamily member 1	0.96	0.329	0.292
ST6GALNAC1	Alpha-N-acetylgalactosaminide alpha-2,6-sialyltransferase 1	1.81	0.090	0.147
TRH	Pro-thyrotropin-releasing hormone	1.28	0.592	0.402
CYCS	Cytochrome c	0.93	0.049	0.100
SERPINA6	Corticosteroid-binding globulin	1.09	0.255	0.252
HYAL1	Hyaluronidase-1	0.96	0.711	0.441
LILRA2	Leukocyte immunoglobulin-like receptor subfamily A member 2	1.54	0.325	0.290
ATP1B1	Sodium/potassium-transporting ATPase subunit beta-1	0.72	0.143	0.186
B4GALT1	Beta-1,4-galactosyltransferase 1	1.36	0.007	0.028
DSC3	Desmocollin-3	1.10	0.339	0.297
DMKN	Dermokine	1.19	0.004	0.021
EGFR	Epidermal growth factor receptor	0.26	0.010	0.036
DSG2	Desmoglein-2	0.93	0.037	0.084
GCHFR	GTP cyclohydrolase 1 feedback regulatory protein	1.10	0.497	0.371
SRBD1	S1 RNA-binding domain-containing protein 1	0.94	0.353	0.303
COL4A2	Collagen alpha-2(IV) chain	0.79	0.375	0.316
DAG1	Dystroglycan	1.04	0.159	0.196
ERP44	Endoplasmic reticulum resident protein 44	1.24	0.498	0.371

F13B	Coagulation factor XIII B chain	0.81	0.609	0.408
F12	Coagulation factor XII	0.97	0.708	0.441
GDF11	Growth/differentiation factor 11	1.10	0.044	0.093
GPR56	G-protein coupled receptor 56	1.22	0.018	0.054
MATN3	Matrilin-3	1.23	0.053	0.104
ST6GAL2	Beta-galactoside alpha-2,6-sialyltransferase 2	1.14	0.025	0.070
XYLT1	Xylosyltransferase 1	1.28	0.026	0.070
ANGPTL1	Angiopoietin-related protein 1	1.07	0.427	0.341
EFEMP1	EGF-containing fibulin-like extracellular matrix protein 1	1.04	0.494	0.370
PBXIP1	Pre-B-cell leukemia transcription factor-interacting protein 1	1.08	0.118	0.166
PCDH10	Protocadherin-10	0.85	0.259	0.254
DNAH5	Dynein heavy chain 5, axonemal	1.28	0.002	0.011
DNAJC3	DnaJ homolog subfamily C member 3	1.06	0.196	0.223
LAMA5	Laminin subunit alpha-5	1.39	0.112	0.162
CDH4	Cadherin-4	1.07	0.091	0.149
GALNS	N-acetylgalactosamine-6-sulfatase	1.20	0.631	0.416
C2orf40	Augurin	0.95	0.554	0.392
APOC2	Apolipoprotein C-II	1.71	0.002	0.011
CST3	Cystatin-C	0.94	0.154	0.194
EFNA3	Ephrin-A3	0.95	0.189	0.218
IGFBP4	Insulin-like growth factor-binding protein 4	0.66	0.080	0.137

CRABP1	Cellular retinoic acid-binding protein 1	1.23	0.000	0.004
S100A4	Protein S100-A4	1.09	0.861	0.483
SLC12A2	Solute carrier family 12 member 2	1.22	0.014	0.046
CLEC3B	Tetranectin	1.01	0.918	0.496
TREM2	Triggering receptor expressed on myeloid cells 2	1.13	0.126	0.174
LAMP2	Lysosome-associated membrane glycoprotein 2	1.02	0.795	0.465
NPEPPS	Puromycin-sensitive aminopeptidase	0.47	0.259	0.254
MAG	Myelin-associated glycoprotein	1.03	0.551	0.391
ATP4A	Potassium-transporting ATPase alpha chain 1	1.01	0.741	0.448
SMPDL3A	Acid sphingomyelinase-like phosphodiesterase 3a	0.92	0.191	0.219
SCN1B	Sodium channel subunit beta-1	0.62	0.108	0.161
APOC3	Apolipoprotein C-III	1.07	0.251	0.251
ADAM28	Disintegrin and metalloproteinase domain-containing protein 28	0.93	0.873	0.484
C4B	Complement C4-B	1.05	0.552	0.391
CXADR	Coxsackievirus and adenovirus receptor	0.94	0.415	0.335
GALNT13	Polypeptide N-acetylgalactosaminyltransferase 13	1.20	0.028	0.073
HDHD2	Haloacid dehalogenase-like hydrolase domain-containing protein 2	3.23	0.198	0.224
MSLN	Mesothelin	1.40	0.000	0.000
OLFM1	Noelin	1.02	0.888	0.489

NUTF2	Nuclear transport factor 2	1.19	0.002	0.009
TMEM132B	Transmembrane protein 132B	1.38	0.028	0.073
LINGO1	Leucine-rich repeat and immunoglobulin-like domain-containing nogo receptor-interacting protein 1	1.13	0.095	0.150
RAD23B	UV excision repair protein RAD23 homolog B	1.04	0.379	0.317
TGFBI	Transforming growth factor-beta-induced protein ig-h3	1.01	0.907	0.494
COTL1	Coactosin-like protein	0.90	0.194	0.222
NTRK3	NT-3 growth factor receptor	0.91	0.282	0.267
PAPLN	Papilin	0.98	0.697	0.440
PFN2	Profilin-2	1.78	0.273	0.261
SECTM1	Secreted and transmembrane protein 1	0.92	0.130	0.176
AP2B1	AP-2 complex subunit beta	0.90	0.075	0.132
CSTB	Cystatin-B	0.97	0.493	0.370
DDR2	Discoidin domain-containing receptor 2	9.60	0.045	0.095
MMRN2	Multimerin-2	1.30	0.643	0.420
PPIC	Peptidyl-prolyl cis-trans isomerase C	1.22	0.089	0.146
PTCHD2	Patched domain-containing protein 2	0.95	0.330	0.292
SLC3A2	4F2 cell-surface antigen heavy chain	1.12	0.001	0.009
ARSA	Arylsulfatase A	1.04	0.726	0.445
C7	Complement component C7	0.88	0.000	0.000
FAM69C	Protein FAM69C	1.08	0.571	0.395

PRCP	Lysosomal Pro-X carboxypeptidase	0.91	0.540	0.389
PRSS1	Trypsin-1	0.93	0.408	0.332
CDH20	Cadherin-20	1.19	0.057	0.110
GLRX	Glutaredoxin-1	0.82	0.214	0.231
SERPIND1	Heparin cofactor 2	1.08	0.095	0.150
LAIR1	Leukocyte-associated immunoglobulin-like receptor 1	1.12	0.503	0.374
MLEC	Malectin	1.02	0.808	0.468
ACHE	Acetylcholinesterase	4.95	0.025	0.069
CLIC4	Chloride intracellular channel protein 4	1.22	0.203	0.226
EMC10	ER membrane protein complex subunit 10	0.92	0.518	0.380
PRKCSH	Glucosidase 2 subunit beta	1.01	0.765	0.456
GNPTG	N-acetylglucosamine-1-phosphotransferase subunit gamma	1.12	0.000	0.000
PAMR1	Inactive serine protease PAMR1	1.63	0.290	0.272
PCSK9	Proprotein convertase subtilisin/kexin type 9	1.18	0.195	0.222
TMED4	Transmembrane emp24 domain-containing protein 4	1.36	0.001	0.009
C16orf89	UPF0764 protein C16orf89	1.59	0.004	0.019
FRMPD1	FERM and PDZ domain-containing protein 1	1.02	0.707	0.441
HYI	Putative hydroxypyruvate isomerase	0.79	0.106	0.160
CDH1	Cadherin-1	0.92	0.575	0.397
CTSB	Cathepsin B	1.01	0.705	0.441

B3GALNT1	UDP-GalNAc:beta-1,3-N-acetylgalactosaminyltransferase 1	0.92	0.099	0.153
C1QB	Complement C1q subcomponent subunit B	1.02	0.680	0.436
CXCL16	C-X-C motif chemokine 16	1.14	0.018	0.054
FAM198B	Protein FAM198B	1.23	0.001	0.009
GGCT	Gamma-glutamylcyclotransferase	1.04	0.753	0.453
ICAM1	Intercellular adhesion molecule 1	0.94	0.743	0.449
SFTPD	Pulmonary surfactant-associated protein D	0.99	0.934	0.503
TTR	Transthyretin	0.85	0.000	0.002
PCP4	Purkinje cell protein 4	0.91	0.214	0.231
POMGNT1	Protein O-linked-mannose beta-1,2-N-acetylglucosaminyltransferase 1	1.24	0.000	0.001
BDNF	Brain-derived neurotrophic factor	0.94	0.349	0.303
C1QC	Complement C1q subcomponent subunit C	1.01	0.829	0.475
HSP90B1	Endoplasmin	1.60	0.077	0.134
NXPH1	Neurexophilin-1	0.98	0.703	0.441
TPM3	Tropomyosin alpha-3 chain	0.39	0.082	0.140
GSTO1	Glutathione S-transferase omega-1	1.24	0.212	0.231
PLEKHB1	Pleckstrin homology domain-containing family B member 1	1.10	0.184	0.214
CNTN5	Contactin-5	0.64	0.040	0.090
GSS	Glutathione synthetase	1.21	0.018	0.055
JAM2	Junctional adhesion molecule B	0.98	0.707	0.441
MAN2A1	Alpha-mannosidase 2	1.00	0.996	0.519

PRRT1	Proline-rich transmembrane protein 1	1.12	0.097	0.151
YIPF3	Protein YIPF3	1.10	0.254	0.252
GANAB	Neutral alpha-glucosidase AB	1.32	0.297	0.276
ESD	S-formylglutathione hydrolase	2.93	0.157	0.195
SPARC	SPARC	0.99	0.582	0.398
KIAA1467	Uncharacterized protein KIAA1467	1.31	0.616	0.412
KLK7	Kallikrein-7	0.98	0.845	0.481
CLSTN3	Calsyntenin-3	1.16	0.002	0.013
GALNT6	Polypeptide N-acetylgalactosaminyltransferase 6	0.62	0.029	0.074
C4A	Complement C4-A	1.45	0.289	0.271
FLNC	Filamin-C	1.02	0.518	0.380
GALNT1	Polypeptide N-acetylgalactosaminyltransferase 1	0.91	0.550	0.391
CLSTN2	Calsyntenin-2	0.96	0.483	0.365
HSPA8	Heat shock cognate 71 kDa protein	1.03	0.767	0.457
KLK10	Kallikrein-10	0.98	0.853	0.481
MANBA	Beta-mannosidase	0.80	0.075	0.131
MGAT1	Alpha-1,3-mannosyl-glycoprotein 2-beta-N-acetylglucosaminyltransferase	1.25	0.086	0.144
NUCB2	Nucleobindin-2	0.89	0.715	0.442
PILRA	Paired immunoglobulin-like type 2 receptor alpha	1.15	0.139	0.183
TNR	Tenascin-R	1.06	0.594	0.402

THBS4	Thrombospondin-4	0.86	0.483	0.365
CCDC126	Coiled-coil domain-containing protein 126	1.06	0.337	0.295
KRT14	Keratin, type I cytoskeletal 14	1.04	0.666	0.430
S100A12	Protein S100-A12	1.03	0.797	0.466
MMP14	Matrix metalloproteinase-14	1.11	0.135	0.180
STX1B	Syntaxin-1B	0.96	0.476	0.364
ATP1A2	Sodium/potassium-transporting ATPase subunit alpha-2	1.14	0.036	0.083
CRISP3	Cysteine-rich secretory protein 3	0.86	0.415	0.335
IGFBP2	Insulin-like growth factor-binding protein 2	1.05	0.472	0.362
SERPINA5	Plasma serine protease inhibitor	1.20	0.159	0.196
SAA1	Serum amyloid A-1 protein	1.32	0.507	0.375
LGALS1	Galectin-1	1.47	0.009	0.034
CRP	C-reactive protein	1.26	0.038	0.086
VCAM1	Vascular cell adhesion protein 1	1.07	0.155	0.194
CSPG4	Chondroitin sulfate proteoglycan 4	0.97	0.143	0.186
SLFN14	Schlafen family member 14	1.00	0.994	0.519
TGFBR3	Transforming growth factor beta receptor type 3	1.13	0.000	0.000
HYOU1	Hypoxia up-regulated protein 1	1.05	0.569	0.395
SCN2B	Sodium channel subunit beta-2	0.80	0.409	0.332
SH3BGRL3	SH3 domain-binding glutamic acid-rich-like protein 3	1.02	0.577	0.398
SORT1	Sortilin	1.06	0.330	0.292

LILRB4	Leukocyte immunoglobulin-like receptor subfamily B member 4	1.89	0.133	0.178
TONSL	Tonsoku-like protein	0.99	0.909	0.494
SHISA6	Protein shisa-6 homolog	1.13	0.066	0.122
CACHD1	VWFA and cache domain-containing protein 1	1.04	0.602	0.405
MANEA	Glycoprotein endo-alpha-1,2-mannosidase	1.05	0.527	0.383
PTPRZ1	Receptor-type tyrosine-protein phosphatase zeta	1.11	0.033	0.079
ADM	ADM	0.99	0.830	0.475
NDRG4	Protein NDRG4	1.14	0.056	0.108
NPTN	Neuroplastin	0.76	0.027	0.073
SERPINA7	Thyroxine-binding globulin	0.94	0.211	0.231
FMOD	Fibromodulin	0.99	0.902	0.494
LAMC3	Laminin subunit gamma-3	1.54	0.201	0.225
SLC8A2	Sodium/calcium exchanger 2	0.99	0.909	0.494
PDIA3	Protein disulfide-isomerase A3	1.04	0.387	0.320
S100A9	Protein S100-A9	1.00	0.974	0.515

Supplementary Table S2. Factors detected via the metabolomic analysis.

Metabolite	Class	Fold change	p-value	q-value
glycine	Amino Acid	1.12	0.176	0.537
N-acetylglycine	Amino Acid	0.96	0.559	0.819
dimethylglycine	Amino Acid	1.21	0.225	0.602
betaine	Amino Acid	1.04	0.481	0.774
serine	Amino Acid	1.10	0.109	0.476
N-acetylserine	Amino Acid	0.99	0.789	0.966
threonine	Amino Acid	1.06	0.435	0.763
N-acetylthreonine	Amino Acid	1.06	0.435	0.763
alanine	Amino Acid	1.07	0.340	0.711
N-acetylalanine	Amino Acid	1.07	0.298	0.669
aspartate	Amino Acid	0.63	0.028	0.359
N-acetylaspartate (NAA)	Amino Acid	1.02	0.869	>0.999
asparagine	Amino Acid	1.10	0.067	0.388
N-acetylasparagine	Amino Acid	0.94	0.307	0.675
hydroxyasparagine	Amino Acid	1.24	0.012	0.359
glutamate	Amino Acid	0.58	0.008	0.359
glutamine	Amino Acid	1.07	0.164	0.537
alpha-ketoglutaramate	Amino Acid	1.05	0.558	0.819
N-acetylglutamate	Amino Acid	0.93	0.479	0.774
N-acetylglutamine	Amino Acid	0.99	0.897	>0.999

glutamate, gamma-methyl ester	Amino Acid	0.96	0.640	0.870
pyroglutamine	Amino Acid	1.05	0.669	0.901
N-acetyl-aspartyl-glutamate (NAAG)	Amino Acid	1.10	0.511	0.789
beta-citrylglutamate	Amino Acid	1.35	0.052	0.359
carboxyethyl-GABA	Amino Acid	1.04	0.561	0.819
S-1-pyrroline-5-carboxylate	Amino Acid	1.69	0.038	0.359
citramalate	Amino Acid	0.76	0.119	0.476
histidine	Amino Acid	1.07	0.367	0.729
1-methylhistidine	Amino Acid	1.18	0.190	0.557
3-methylhistidine	Amino Acid	1.23	0.271	0.644
N-acetylhistidine	Amino Acid	1.02	0.883	>0.999
N-acetyl-3-methylhistidine	Amino Acid	1.39	0.218	0.597
formiminoglutamate	Amino Acid	1.28	0.167	0.537
imidazole lactate	Amino Acid	1.11	0.464	0.774
homocarnosine	Amino Acid	0.80	0.260	0.634
N-acetylcarnosine	Amino Acid	1.07	0.627	0.861
1-methyl-4-imidazoleacetate	Amino Acid	1.09	0.630	0.861
1-ribosyl-imidazoleacetate*	Amino Acid	0.99	0.911	>0.999
lysine	Amino Acid	0.96	0.560	0.819
N6,N6,N6-trimethyllysine	Amino Acid	1.05	0.407	0.748
5-(galactosylhydroxy)-L-lysine	Amino Acid	1.22	0.071	0.389

glutaroylcarnitine (C5)	Amino Acid	1.12	0.430	0.763
pipecolate	Amino Acid	0.92	0.512	0.789
6-oxopiperidine-2-carboxylate	Amino Acid	0.93	0.449	0.769
N,N,N-trimethyl-5-aminovalerate	Amino Acid	0.91	0.532	0.806
phenylalanine	Amino Acid	1.05	0.480	0.774
N-acetylphenylalanine	Amino Acid	0.99	0.912	>0.999
1-carboxyethylphenylalanine	Amino Acid	1.15	0.386	0.738
phenyllactate (PLA)	Amino Acid	1.27	0.062	0.368
tyrosine	Amino Acid	0.99	0.866	>0.999
3-(4-hydroxyphenyl)lactate (HPLA)	Amino Acid	1.14	0.361	0.729
phenol sulfate	Amino Acid	1.90	0.049	0.359
3-methoxytyrosine	Amino Acid	4.81	0.130	0.491
homovanillate (HVA)	Amino Acid	1.19	0.202	0.574
dopamine 3-O-sulfate	Amino Acid	2.83	0.177	0.537
tryptophan	Amino Acid	1.04	0.676	0.906
C-glycosyltryptophan	Amino Acid	1.09	0.262	0.634
tryptophan betaine	Amino Acid	0.80	0.687	0.912
kynurenine	Amino Acid	1.24	0.057	0.359
kynurenate	Amino Acid	1.46	0.021	0.359
N-formylanthranilic acid	Amino Acid	1.29	0.532	0.806
anthranilate	Amino Acid	0.97	0.914	>0.999

5-hydroxyindoleacetate	Amino Acid	1.28	0.170	0.537
indoleacetate	Amino Acid	1.43	0.026	0.359
3-indoxyl sulfate	Amino Acid	1.24	0.332	0.705
leucine	Amino Acid	1.02	0.726	0.919
N-acetylleucine	Amino Acid	1.11	0.378	0.738
4-methyl-2-oxopentanoate	Amino Acid	1.99	0.048	0.359
alpha-hydroxyisocaproate	Amino Acid	1.09	0.288	0.661
isovalerate (C5)	Amino Acid	0.90	0.397	0.746
isovalerylcarnitine (C5)	Amino Acid	1.08	0.421	0.758
beta-hydroxyisovalerate	Amino Acid	0.86	0.100	0.460
3-methylglutaconate	Amino Acid	1.09	0.234	0.607
3-methylglutarylcarnitine (2)	Amino Acid	1.23	0.043	0.359
isoleucine	Amino Acid	1.06	0.405	0.748
N-acetylisoleucine	Amino Acid	0.97	0.735	0.920
3-methyl-2-oxovalerate	Amino Acid	1.63	0.084	0.427
alpha-hydroxyisovalerate	Amino Acid	1.21	0.427	0.763
2-methylbutyroylcarnitine (C5)	Amino Acid	1.13	0.207	0.575
tiglyl carnitine (C5)	Amino Acid	1.18	0.020	0.359
3-hydroxy-2-ethylpropionate	Amino Acid	1.04	0.627	0.861
ethylmalonate	Amino Acid	1.01	0.902	>0.999
methylsuccinoylcarnitine	Amino Acid	1.07	0.567	0.821

valine	Amino Acid	1.04	0.542	0.818
N-acetylvaline	Amino Acid	1.05	0.512	0.789
1-carboxyethylvaline	Amino Acid	1.07	0.555	0.819
3-methyl-2-oxobutyrate	Amino Acid	1.75	0.122	0.476
2-hydroxy-3-methylvalerate	Amino Acid	1.06	0.745	0.929
isobutyrylcarnitine (C4)	Amino Acid	1.23	0.002	0.216
3-hydroxyisobutyrate	Amino Acid	1.10	0.259	0.634
2,3-dihydroxy-2-methylbutyrate	Amino Acid	0.78	0.384	0.738
N-acetylmethionine	Amino Acid	1.37	0.071	0.389
N-formylmethionine	Amino Acid	1.40	0.049	0.359
S-methylmethionine	Amino Acid	0.09	0.305	0.675
methionine sulfone	Amino Acid	0.95	0.834	0.994
methionine sulfoxide	Amino Acid	0.69	0.020	0.359
N-acetylmethionine sulfoxide	Amino Acid	0.73	0.248	0.627
5-methylthioribose	Amino Acid	1.02	0.716	0.919
cystathionine	Amino Acid	1.09	0.686	0.912
cysteine	Amino Acid	1.26	0.023	0.359
S-methylcysteine	Amino Acid	1.11	0.555	0.819
S-methylcysteine sulfoxide	Amino Acid	0.84	0.228	0.602
cystine	Amino Acid	1.15	0.468	0.774
taurine	Amino Acid	0.97	0.723	0.919
N-acetyltaurine	Amino Acid	0.97	0.849	>0.999

arginine	Amino Acid	0.99	0.836	0.994
argininosuccinate	Amino Acid	1.05	0.497	0.787
urea	Amino Acid	1.04	0.582	0.833
ornithine	Amino Acid	1.10	0.122	0.476
2-oxoarginine*	Amino Acid	1.92	0.024	0.359
citrulline	Amino Acid	1.05	0.476	0.774
homoarginine	Amino Acid	0.83	0.054	0.359
proline	Amino Acid	1.03	0.847	>0.999
dimethylarginine (ADMA + SDMA)	Amino Acid	1.18	0.012	0.359
N-acetylarginine	Amino Acid	1.02	0.773	0.950
N-delta-acetylornithine	Amino Acid	1.04	0.828	0.994
N-alpha-acetylornithine	Amino Acid	0.84	0.139	0.496
hydroxyproline	Amino Acid	1.14	0.120	0.476
prolylhydroxyproline	Amino Acid	1.29	0.044	0.359
N-methylproline	Amino Acid	0.78	0.502	0.788
argininate	Amino Acid	1.11	0.360	0.729
creatine	Amino Acid	1.05	0.081	0.417
creatinine	Amino Acid	1.06	0.170	0.537
spermidine	Amino Acid	1.14	0.334	0.705
diacetylspermidine	Amino Acid	1.20	0.039	0.359
acisoga	Amino Acid	1.59	0.009	0.359

5-methylthioadenosine (MTA)	Amino Acid	1.05	0.490	0.781
N-acetylputrescine	Amino Acid	1.06	0.452	0.770
4-acetamidobutanoate	Amino Acid	1.03	0.723	0.919
(N(1) + N(8))-acetylspermidine	Amino Acid	1.18	0.040	0.359
4-guanidinobutanoate	Amino Acid	0.81	0.134	0.491
cysteinylglycine	Amino Acid	1.22	0.191	0.557
cysteinylglycine disulfide	Amino Acid	0.98	0.833	0.994
cys-gly, oxidized	Amino Acid	0.78	0.037	0.359
5-oxoproline	Amino Acid	0.75	0.039	0.359
2-aminobutyrate	Amino Acid	1.05	0.510	0.789
2-hydroxybutyrate/2-hydroxyisobutyrate	Amino Acid	1.17	0.170	0.537
gamma-glutamylalanine	Peptide	1.23	0.049	0.359
gamma-glutamylglutamate	Peptide	0.60	0.030	0.359
gamma-glutamylglutamine	Peptide	0.92	0.256	0.634
gamma-glutamylhistidine	Peptide	0.95	0.598	0.848
gamma-glutamylisoleucine	Peptide	0.94	0.570	0.823
gamma-glutamylleucine	Peptide	0.88	0.433	0.763
gamma-glutamyl-alpha-lysine	Peptide	0.86	0.044	0.359
gamma-glutamyl-epsilon-lysine	Peptide	0.69	0.026	0.359
gamma-glutamylmethionine	Peptide	1.17	0.436	0.763
gamma-glutamylphenylalanine	Peptide	0.99	0.954	>0.999

gamma-glutamylthreonine	Peptide	1.01	0.900	>0.999
gamma-glutamyltyrosine	Peptide	0.95	0.629	0.861
gamma-glutamylvaline	Peptide	0.96	0.625	0.861
gamma-glutamylcitrulline	Peptide	0.94	0.712	0.919
gamma-glutamyl-2-aminobutyrate	Peptide	1.17	0.280	0.657
cyclo(ala-pro)	Peptide	0.80	0.136	0.494
cyclo(leu-pro)	Peptide	0.52	0.050	0.359
cyclo(met-pro)	Peptide	1.03	0.853	>0.999
cyclo(phe-pro)	Peptide	0.46	0.160	0.537
cyclo(L-phe-D-pro)	Peptide	0.55	0.205	0.575
cyclo(pro-val)	Peptide	0.67	0.026	0.359
glycylproline	Peptide	0.83	0.186	0.555
prolylglycine	Peptide	0.82	0.287	0.661
prolylproline	Peptide	0.94	0.447	0.769
phenylacetylglutamine	Peptide	1.67	0.026	0.359
1,5-anhydroglucitol (1,5-AG)	Carbohydrate	1.02	0.891	>0.999
glucose	Carbohydrate	1.06	0.203	0.574
pyruvate	Carbohydrate	1.57	0.049	0.359
lactate	Carbohydrate	1.07	0.237	0.610
glycerate	Carbohydrate	0.84	0.112	0.476
ribitol	Carbohydrate	1.01	0.867	>0.999

ribonate (ribonolactone)	Carbohydrate	1.17	0.068	0.388
arabinose	Carbohydrate	1.01	0.888	>0.999
arabitol/xylitol	Carbohydrate	1.04	0.564	0.820
arabonate/xylonate	Carbohydrate	1.31	0.031	0.359
sedoheptulose	Carbohydrate	0.98	0.890	>0.999
ribulonate/xylulonate	Carbohydrate	1.14	0.311	0.675
sucrose	Carbohydrate	0.71	0.290	0.662
fructose	Carbohydrate	1.08	0.310	0.675
mannitol/sorbitol	Carbohydrate	1.16	0.044	0.359
mannose	Carbohydrate	1.18	0.018	0.359
galactonate	Carbohydrate	1.78	0.055	0.359
glucuronate	Carbohydrate	1.03	0.772	0.950
N-acetylneuraminate	Carbohydrate	1.07	0.387	0.738
N-acetylglucosaminylasparagine	Carbohydrate	1.19	0.313	0.675
erythronate	Carbohydrate	1.08	0.121	0.476
N-acetylglucosamine/N-acetylgalactosamine	Carbohydrate	1.10	0.089	0.429
citrate	Energy	1.12	0.107	0.476
isocitrate	Energy	1.77	0.098	0.459
alpha-ketoglutarate	Energy	1.56	0.096	0.453
succinylcarnitine (C4)	Energy	1.12	0.111	0.476
succinate	Energy	0.93	0.650	0.877

2-methylcitrate/homocitrate	Energy	1.14	0.041	0.359
phosphate	Energy	1.00	0.866	>0.999
malonylcarnitine	Lipid	1.17	0.133	0.491
malonate	Lipid	0.99	0.987	>0.999
caproate (6:0)	Lipid	1.10	0.513	0.789
dimethylmalonic acid	Lipid	1.13	0.238	0.610
glutarate (C5-DC)	Lipid	0.94	0.710	0.919
2-hydroxyadipate	Lipid	0.92	0.628	0.861
3-carboxy-4-methyl-5-propyl-2-furanpropanoate (CMPF)	Lipid	1.10	0.697	0.916
hydroxy-CMPF	Lipid	0.97	0.836	0.994
2-aminoheptanoate	Lipid	1.26	0.155	0.536
2-aminooctanoate	Lipid	0.76	0.173	0.537
propionylcarnitine (C3)	Lipid	1.27	0.021	0.359
methylmalonate (MMA)	Lipid	0.89	0.175	0.537
acetylcarnitine (C2)	Lipid	1.81	0.044	0.359
octanoylcarnitine (C8)	Lipid	1.12	0.383	0.738
decanoylcarnitine (C10)	Lipid	1.25	0.137	0.494
cis-4-decenoylcarnitine (C10:1)	Lipid	1.33	0.032	0.359
myristoleoylcarnitine (C14:1)	Lipid	0.88	0.175	0.537
deoxycarnitine	Lipid	1.01	0.944	>0.999
carnitine	Lipid	1.06	0.396	0.746

3-hydroxybutyrate (BHBA)	Lipid	4.69	0.231	0.602
3-hydroxyhexanoate	Lipid	1.18	0.118	0.476
3-hydroxyoctanoate	Lipid	1.06	0.719	0.919
myo-inositol	Lipid	1.12	0.450	0.769
chiro-inositol	Lipid	0.60	0.174	0.537
choline	Lipid	1.07	0.207	0.575
phosphocholine	Lipid	1.02	0.820	0.994
phosphoethanolamine (PE)	Lipid	1.01	0.948	>0.999
glycerophosphoethanolamine	Lipid	1.01	0.958	>0.999
glycerophosphoinositol	Lipid	1.15	0.090	0.429
trimethylamine N-oxide	Lipid	1.01	0.950	>0.999
galactosylglycerol	Lipid	1.10	0.256	0.634
glycerol	Lipid	1.14	0.354	0.728
glycerol 3-phosphate	Lipid	0.94	0.591	0.841
glycerophosphoglycerol	Lipid	1.04	0.770	0.950
3-hydroxy-3-methylglutarate	Lipid	1.07	0.497	0.787
cholesterol	Lipid	1.13	0.111	0.476
7-HOCA	Lipid	1.01	0.936	>0.999
cortisol	Lipid	1.29	0.119	0.476
cortisone	Lipid	1.14	0.400	0.748
androstenediol (3beta,17beta) disulfate (1)	Lipid	1.13	0.840	0.995

chenodeoxycholate	Lipid	0.82	0.691	0.915
glycochenodeoxycholate	Lipid	0.61	0.307	0.675
deoxycholate	Lipid	1.31	0.469	0.774
inosine	Nucleotide	0.99	0.901	>0.999
hypoxanthine	Nucleotide	0.97	0.805	0.981
xanthine	Nucleotide	1.08	0.396	0.746
N1-methylinosine	Nucleotide	1.19	0.087	0.427
urate	Nucleotide	1.19	0.170	0.537
allantoin	Nucleotide	0.86	0.331	0.705
adenosine	Nucleotide	1.04	0.825	0.994
adenine	Nucleotide	1.04	0.713	0.919
1-methyladenosine	Nucleotide	1.18	0.054	0.359
N6-methyladenosine	Nucleotide	1.01	0.958	>0.999
N6-carbamoylthreonyladenosine	Nucleotide	1.21	0.062	0.368
N6-succinyladenosine	Nucleotide	1.11	0.382	0.738
guanosine	Nucleotide	1.10	0.055	0.359
7-methylguanine	Nucleotide	1.18	0.016	0.359
N2,N2-dimethylguanosine	Nucleotide	1.18	0.015	0.359
orotate	Nucleotide	0.92	0.624	0.861
orotidine	Nucleotide	1.06	0.700	0.917
uridine	Nucleotide	0.92	0.365	0.729

pseudouridine	Nucleotide	1.17	0.007	0.359
5,6-dihydrouridine	Nucleotide	1.23	0.007	0.359
5-methyluridine (ribothymidine)	Nucleotide	0.99	0.866	>0.999
5,6-dihydrouracil	Nucleotide	1.16	0.080	0.417
2'-deoxyuridine	Nucleotide	0.90	0.379	0.738
3-ureidopropionate	Nucleotide	0.90	0.456	0.774
N-acetyl-beta-alanine	Nucleotide	0.99	0.926	>0.999
cytidine	Nucleotide	1.00	0.916	>0.999
cytosine	Nucleotide	1.14	0.551	0.819
3-methylcytidine	Nucleotide	1.24	0.230	0.602
5-methylcytidine	Nucleotide	1.08	0.156	0.536
N4-acetylcytidine	Nucleotide	1.38	0.104	0.473
2'-deoxycytidine	Nucleotide	0.90	0.056	0.359
2'-O-methylcytidine	Nucleotide	1.09	0.473	0.774
5,6-dihydrothymine	Nucleotide	1.08	0.194	0.558
3-aminoisobutyrate	Nucleotide	1.28	0.077	0.410
nicotinamide riboside	Cofactors and Vitamins	1.12	0.245	0.623
1-methylnicotinamide	Cofactors and Vitamins	1.17	0.477	0.774
trigonelline (N'-methylnicotinate)	Cofactors and Vitamins	0.74	0.261	0.634
N1-Methyl-2-pyridone-5-carboxamide	Cofactors and Vitamins	1.00	0.983	>0.999
N1-Methyl-4-pyridone-3-carboxamide	Cofactors and Vitamins	1.17	0.480	0.774

pantothenate (Vitamin B5)	Cofactors and Vitamins	0.77	0.309	0.675
ascorbate (Vitamin C)	Cofactors and Vitamins	2.59	0.185	0.555
threonate	Cofactors and Vitamins	0.80	0.061	0.368
oxalate (ethanedioate)	Cofactors and Vitamins	1.01	0.900	>0.999
gulonate	Cofactors and Vitamins	1.08	0.438	0.763
alpha-tocopherol	Cofactors and Vitamins	0.94	0.604	0.853
gamma-tocopherol/beta-tocopherol	Cofactors and Vitamins	0.96	0.879	>0.999
heme	Cofactors and Vitamins	3.41	0.366	0.729
bilirubin	Cofactors and Vitamins	1.32	0.220	0.598
bilirubin (E,E)	Cofactors and Vitamins	0.88	0.630	0.861
retinol (Vitamin A)	Cofactors and Vitamins	1.00	0.981	>0.999
carotene diol (1)	Cofactors and Vitamins	0.94	0.793	0.969
pyridoxamine	Cofactors and Vitamins	0.98	0.947	>0.999
pyridoxate	Cofactors and Vitamins	1.19	0.546	0.818
hippurate	Xenobiotics	1.25	0.269	0.644
benzoate	Xenobiotics	1.38	0.134	0.491
catechol sulfate	Xenobiotics	1.14	0.682	0.912
guaiacol sulfate	Xenobiotics	1.05	0.893	>0.999
p-cresol sulfate	Xenobiotics	2.11	0.014	0.359
caffeine	Xenobiotics	0.42	0.033	0.359
paraxanthine	Xenobiotics	0.61	0.037	0.359

theobromine	Xenobiotics	0.49	0.052	0.359
theophylline	Xenobiotics	2.81	0.363	0.729
3-methylxanthine	Xenobiotics	2.02	0.378	0.738
7-methylxanthine	Xenobiotics	0.62	0.132	0.491
5-acetylamino-6-amino-3-methyluracil	Xenobiotics	1.03	0.914	>0.999
cotinine	Xenobiotics	1.43	0.702	0.917
hydroxycotinine	Xenobiotics	0.29	0.474	0.774
2-piperidinone	Xenobiotics	0.88	0.631	0.861
2,3-dihydroxyisovalerate	Xenobiotics	0.74	0.298	0.669
3-hydroxyindolin-2-one	Xenobiotics	1.43	0.143	0.508
gluconate	Xenobiotics	1.26	0.019	0.359
N-acetylalliin	Xenobiotics	0.73	0.411	0.752
ergothioneine	Xenobiotics	2.14	0.036	0.359
erythritol	Xenobiotics	1.14	0.336	0.705
histidine betaine (hercynine)	Xenobiotics	0.91	0.715	0.919
homostachydrine	Xenobiotics	0.82	0.294	0.667
piperine	Xenobiotics	0.77	0.584	0.833
quinate	Xenobiotics	1.16	0.648	0.877
S-allylcysteine	Xenobiotics	0.74	0.545	0.818
stachydrine	Xenobiotics	0.70	0.406	0.748
3-hydroxystachydrine*	Xenobiotics	0.51	0.192	0.557

tartarate	Xenobiotics	0.99	0.974	>0.999
methyl glucopyranoside (alpha + beta)	Xenobiotics	0.83	0.482	0.774
tartronate (hydroxymalonate)	Xenobiotics	0.71	0.034	0.359
N-methylpipecolate	Xenobiotics	1.14	0.418	0.758
4-acetamidophenol	Xenobiotics			
4-acetaminophen sulfate	Xenobiotics			
4-acetamidophenylglucuronide	Xenobiotics			
lidocaine	Xenobiotics	0.13	0.228	0.602
metformin	Xenobiotics			
allopurinol	Xenobiotics			
oxypurinol	Xenobiotics			
carbamazepine	Xenobiotics			
carbamazepine 10,11-epoxide	Xenobiotics			
pregabalin	Xenobiotics			
salicylate	Xenobiotics	1.68	0.623	0.861
diglycerol	Xenobiotics	0.92	0.581	0.833
sulfate	Xenobiotics	1.13	0.015	0.359
O-sulfo-L-tyrosine	Xenobiotics	1.28	0.006	0.359
ethyl glucuronide	Xenobiotics	2.52	0.269	0.644
dimethyl sulfone	Xenobiotics	0.78	0.274	0.646
succinimide	Xenobiotics	0.77	0.070	0.389

3-hydroxypyridine sulfate	Xenobiotics	0.90	0.730	0.919
thioproline	Xenobiotics	1.06	0.732	0.919
X - 10445		0.95	0.753	0.937
X - 10457		1.12	0.283	0.660
X - 11261		1.27	0.087	0.427
X - 11612		1.22	0.216	0.595
X - 11787		0.96	0.526	0.803
X - 11795		0.99	0.946	>0.999
X - 12007		0.81	0.448	0.769
X - 12100		1.29	0.008	0.359
X - 12101		1.59	0.051	0.359
X - 12104		1.21	0.087	0.427
X - 12127		2.94	0.349	0.726
X - 12206		1.08	0.460	0.774
X - 12411		2.09	0.000	0.088
X - 12680		0.83	0.034	0.359
X - 12689		0.96	0.763	0.946
X - 12906		1.05	0.523	0.801
X - 14056		1.24	0.191	0.557
X - 14838		1.25	0.420	0.758
X - 15674		1.08	0.865	>0.999

X - 16938		0.87	0.227	0.602
X - 17299		1.42	0.000	0.088
X - 21286		1.39	0.364	0.729
X - 21729		0.83	0.156	0.536
X - 21733		1.26	0.354	0.728
X - 21785		1.09	0.170	0.537
X - 22162		1.01	0.929	>0.999
X - 22775		0.85	0.114	0.476
X - 23195		0.99	0.883	>0.999
X - 23314		0.69	0.422	0.758
X - 23590		0.92	0.316	0.678
X - 23593		1.10	0.288	0.661
X - 23639		0.98	0.831	0.994
X - 23644		0.44	0.170	0.537
X - 23666		1.28	0.113	0.476
X - 23739		0.99	0.907	>0.999
X - 24228		1.11	0.122	0.476
X - 24293		0.35	0.130	0.491
X - 24452		1.08	0.259	0.634
X - 24455		1.08	0.501	0.788
X - 24686		0.72	0.058	0.359

X - 24699		1.15	0.076	0.409
X - 24728		1.24	0.068	0.388
X - 24736		1.02	0.968	>0.999
X - 24952		1.13	0.489	0.781
X - 25109		1.08	0.694	0.915
X - 25182		1.29	0.405	0.748
X - 25271		0.76	0.730	0.919
X - 25416		0.66	0.019	0.359
X - 25422		1.16	0.157	0.536

Supplementary Table S3. Factors detected via the lipidomic analysis.

Lipid	Class	Fold change	p-value	q-value
CE(12:0)	CE	N.D.		
CE(14:0)	CE	-0.07	0.667	0.961
CE(14:1)	CE	-1.02	0.284	0.894
CE(15:0)	CE	0.11	0.470	0.894
CE(16:0)	CE	0.19	0.123	0.894
CE(16:1)	CE	0.07	0.639	0.953
CE(17:0)	CE	-0.06	0.790	0.986
CE(18:0)	CE	0.11	0.426	0.894
CE(18:1)	CE	0.11	0.392	0.894
CE(18:2)	CE	0.01	0.982	>0.999
CE(18:3)	CE	-0.24	0.439	0.894
CE(18:4)	CE	-0.59	0.401	0.894
CE(20:0)	CE	-1.41	0.403	0.894
CE(20:1)	CE	-0.66	0.601	0.939
CE(20:2)	CE	-0.52	0.242	0.894
CE(20:3)	CE	0.15	0.436	0.894
CE(20:4)	CE	0.12	0.526	0.906
CE(20:5)	CE	-0.10	0.741	0.972
CE(22:0)	CE	N.D.		

CE(22:1)	CE	N.D.		
CE(22:2)	CE	N.D.		
CE(22:4)	CE	-0.67	0.307	0.894
CE(22:5)	CE	-0.11	0.715	0.961
CE(22:6)	CE	0.05	0.741	0.972
CE(24:0)	CE	N.D.		
CE(24:1)	CE	N.D.		
CER(14:0)	CER	-0.04	0.405	0.894
CER(16:0)	CER	-0.01	0.967	>0.999
CER(18:0)	CER	0.21	0.067	0.894
CER(20:0)	CER	-0.19	0.698	0.961
CER(22:0)	CER	-0.30	0.181	0.894
CER(22:1)	CER	-0.06	0.168	0.894
CER(24:0)	CER	-0.08	0.702	0.961
CER(24:1)	CER	-0.09	0.540	0.911
DAG(12:0/16:0)	DAG	-1.91	0.266	0.894
DAG(12:0/18:0)	DAG	N.D.		
DAG(12:0/18:1)	DAG	-0.50	0.253	0.894
DAG(12:0/18:2)	DAG	-0.17	0.340	0.894
DAG(14:0/14:0)	DAG	N.D.		
DAG(14:0/16:0)	DAG	0.19	0.710	0.961
DAG(14:0/16:1)	DAG	N.D.		

DAG(14:0/18:1)	DAG	-0.35	0.444	0.894
DAG(14:0/18:2)	DAG	-0.03	0.928	>0.999
DAG(14:0/20:0)	DAG	-0.56	0.278	0.894
DAG(14:0/22:6)	DAG	N.D.		
DAG(14:1/16:0)	DAG	0.87	0.297	0.894
DAG(14:1/18:1)	DAG	0.26	0.379	0.894
DAG(15:0/18:1)	DAG	-0.21	0.575	0.939
DAG(15:0/18:2)	DAG	N.D.		
DAG(16:0/16:0)	DAG	-0.18	0.307	0.894
DAG(16:0/16:1)	DAG	N.D.		
DAG(16:0/18:0)	DAG	-0.14	0.230	0.894
DAG(16:0/18:1)	DAG	-0.70	0.387	0.894
DAG(16:0/18:2)	DAG	-0.30	0.654	0.961
DAG(16:0/18:3)	DAG	N.D.		
DAG(16:0/20:4)	DAG	N.D.		
DAG(16:0/22:5)	DAG	N.D.		
DAG(16:0/22:6)	DAG	N.D.		
DAG(16:1/16:1)	DAG	-1.83	0.074	0.894
DAG(16:1/18:0)	DAG	-0.06	0.857	0.999
DAG(16:1/18:1)	DAG	-0.23	0.292	0.894
DAG(16:1/18:2)	DAG	N.D.		

DAG(16:1/20:0)	DAG	-0.42	0.590	0.939
DAG(16:1/20:2)	DAG	N.D.		
DAG(18:0/18:1)	DAG	-0.06	0.812	0.998
DAG(18:0/18:2)	DAG	N.D.		
DAG(18:0/22:6)	DAG	0.65	0.328	0.894
DAG(18:1/18:1)	DAG	-0.57	0.154	0.894
DAG(18:1/18:2)	DAG	-0.07	0.777	0.986
DAG(18:1/20:1)	DAG	N.D.		
DAG(18:1/20:2)	DAG	N.D.		
DAG(18:1/20:4)	DAG	N.D.		
DAG(18:1/20:5)	DAG	N.D.		
DAG(18:1/22:6)	DAG	-0.11	0.710	0.961
DCER(14:0)	DCER	N.D.		
DCER(16:0)	DCER	-0.39	0.226	0.894
DCER(20:0)	DCER	0.00	>0.999	>0.999
DCER(20:1)	DCER	N.D.		
DCER(22:0)	DCER	-0.15	0.024	0.894
DCER(22:1)	DCER	-0.01	0.893	>0.999
DCER(22:2)	DCER	-0.06	0.353	0.894
DCER(24:0)	DCER	-0.06	0.543	0.914
DCER(24:1)	DCER	-0.05	0.427	0.894

DCER(26:0)	DCER	0.04	0.659	0.961
DCER(26:1)	DCER	-0.04	0.625	0.944
FFA(12:0)	FFA	0.04	0.859	0.999
FFA(14:0)	FFA	-0.06	0.804	0.994
FFA(14:1)	FFA	0.58	0.405	0.894
FFA(15:0)	FFA	0.27	0.548	0.919
FFA(16:0)	FFA	-0.12	0.415	0.894
FFA(16:1)	FFA	-0.46	0.249	0.894
FFA(17:0)	FFA	-0.11	0.270	0.894
FFA(18:0)	FFA	-0.11	0.323	0.894
FFA(18:1)	FFA	-0.21	0.464	0.894
FFA(18:2)	FFA	0.12	0.687	0.961
FFA(18:4)	FFA	-0.05	0.684	0.961
FFA(20:0)	FFA	-0.11	0.417	0.894
FFA(20:1)	FFA	-0.08	0.625	0.944
FFA(20:2)	FFA	-0.12	0.496	0.894
FFA(20:3)	FFA	0.29	0.066	0.894
FFA(20:4)	FFA	0.09	0.644	0.959
FFA(20:5)	FFA	0.05	0.701	0.961
FFA(22:0)	FFA	0.06	0.745	0.972
FFA(22:1)	FFA	-0.05	0.725	0.969

FFA(22:2)	FFA	-0.10	0.664	0.961
FFA(22:4)	FFA	0.03	0.836	0.999
FFA(22:5)	FFA	-0.58	0.417	0.894
FFA(22:6)	FFA	0.10	0.363	0.894
FFA(24:0)	FFA	-0.04	0.789	0.986
FFA(24:1)	FFA	0.01	0.963	>0.999
HCER(16:0)	HCER	0.06	0.687	0.961
HCER(18:0)	HCER	0.06	0.657	0.961
HCER(20:0)	HCER	0.16	0.366	0.894
HCER(22:0)	HCER	0.01	0.968	>0.999
HCER(22:1)	HCER	N.D.		
HCER(24:0)	HCER	0.07	0.599	0.939
HCER(24:1)	HCER	0.19	0.168	0.894
HCER(26:1)	HCER	N.D.		
LCER(14:0)	LCER	-1.11	0.037	0.894
LCER(16:0)	LCER	-0.33	0.304	0.894
LCER(18:0)	LCER	0.05	0.672	0.961
LCER(18:1)	LCER	0.01	0.836	0.999
LCER(20:0)	LCER	N.D.		
LCER(22:1)	LCER	N.D.		
LCER(24:1)	LCER	N.D.		
LPC(16:0)	LPC	N.D.		

LPC(18:0)	LPC	N.D.		
LPC(18:1)	LPC	-0.08	0.821	0.999
LPC(18:2)	LPC	N.D.		
LPC(20:0)	LPC	0.02	0.602	0.939
LPC(20:4)	LPC	N.D.		
LPE(18:0)	LPE	N.D.		
LPE(18:1)	LPE	0.20	0.650	0.961
LPE(20:4)	LPE	N.D.		
LPE(22:6)	LPE	N.D.		
MAG(12:0)	MAG	-0.04	0.786	0.986
MAG(14:0)	MAG	-0.18	0.326	0.894
MAG(14:1)	MAG	N.D.		
MAG(15:0)	MAG	0.40	0.496	0.894
MAG(16:0)	MAG	-0.05	0.827	0.999
MAG(16:1)	MAG	-0.53	0.370	0.894
MAG(17:0)	MAG	-0.23	0.244	0.894
MAG(18:0)	MAG	-0.01	0.939	>0.999
MAG(18:1)	MAG	-0.52	0.048	0.894
MAG(18:2)	MAG	-0.31	0.475	0.894
MAG(20:0)	MAG	0.00	0.991	>0.999
MAG(22:0)	MAG	N.D.		

MAG(22:5)	MAG	N.D.		
PC(14:0/18:1)	PC	0.00	0.990	>0.999
PC(16:0/14:0)	PC	0.00	0.981	>0.999
PC(16:0/16:0)	PC	0.24	0.033	0.894
PC(16:0/16:1)	PC	0.25	0.038	0.894
PC(16:0/18:0)	PC	0.16	0.140	0.894
PC(16:0/18:1)	PC	0.20	0.051	0.894
PC(16:0/18:2)	PC	-0.04	0.869	0.999
PC(16:0/20:1)	PC	0.32	0.192	0.894
PC(16:0/20:2)	PC	-0.76	0.028	0.894
PC(16:0/20:3)	PC	-0.06	0.692	0.961
PC(16:0/20:4)	PC	0.03	0.855	0.999
PC(16:0/20:5)	PC	N.D.		
PC(16:0/22:4)	PC	-0.54	0.151	0.894
PC(16:0/22:5)	PC	0.04	0.868	0.999
PC(16:0/22:6)	PC	-0.10	0.483	0.894
PC(18:0/18:0)	PC	0.09	0.531	0.909
PC(18:0/18:1)	PC	0.11	0.336	0.894
PC(18:0/18:2)	PC	-0.09	0.677	0.961
PC(18:0/20:0)	PC	-0.04	0.667	0.961
PC(18:0/20:3)	PC	-0.03	0.793	0.988
PC(18:0/20:4)	PC	0.03	0.796	0.988

PC(18:0/20:5)	PC	-0.32	0.240	0.894
PC(18:0/22:4)	PC	N.D.		
PC(18:0/22:5)	PC	N.D.		
PC(18:0/22:6)	PC	-0.03	0.835	0.999
PC(18:1/16:1)	PC	0.16	0.083	0.894
PC(18:1/18:1)	PC	0.15	0.075	0.894
PC(18:1/18:2)	PC	-0.04	0.701	0.961
PC(18:1/18:3)	PC	0.29	0.062	0.894
PC(18:1/20:3)	PC	-0.18	0.130	0.894
PC(18:1/20:4)	PC	0.05	0.499	0.894
PC(18:1/20:5)	PC	N.D.		
PC(18:1/22:5)	PC	N.D.		
PC(18:1/22:6)	PC	0.09	0.707	0.961
PC(18:2/16:1)	PC	0.06	0.564	0.933
PC(18:2/18:2)	PC	0.01	0.841	0.999
PC(18:2/18:3)	PC	0.05	0.441	0.894
PC(18:2/20:2)	PC	-0.08	0.711	0.961
PC(18:2/20:3)	PC	0.03	0.550	0.920
PC(18:2/20:4)	PC	0.04	0.472	0.894
PC(18:2/20:5)	PC	0.06	0.335	0.894
PC(18:2/22:4)	PC	0.03	0.637	0.953

PC(18:2/22:5)	PC	0.00	0.993	>0.999
PC(18:2/22:6)	PC	0.06	0.333	0.894
PC(20:0/18:1)	PC	0.06	0.509	0.894
PE(18:0/18:0)	PE	0.06	0.817	0.999
PE(18:0/18:1)	PE	0.10	0.416	0.894
PE(18:0/18:2)	PE	N.D.		
PE(18:0/20:4)	PE	-0.07	0.717	0.961
PE(18:0/22:4)	PE	N.D.		
PE(18:0/22:6)	PE	0.03	0.868	0.999
PE(18:1/18:1)	PE	N.D.		
PE(18:1/22:0)	PE	N.D.		
PE(O-18:0/22:4)	PE	N.D.		
PE(O-18:0/22:6)	PE	0.17	0.503	0.894
PE(P-16:0/18:1)	PE	0.25	0.031	0.894
PE(P-16:0/20:4)	PE	-0.16	0.359	0.894
PE(P-16:0/22:4)	PE	0.09	0.787	0.986
PE(P-16:0/22:6)	PE	-0.11	0.347	0.894
PE(P-18:0/18:1)	PE	0.16	0.193	0.894
PE(P-18:0/18:2)	PE	N.D.		
PE(P-18:0/20:4)	PE	-0.13	0.259	0.894
PE(P-18:0/22:4)	PE	0.07	0.627	0.944
PE(P-18:0/22:5)	PE	N.D.		

PE(P-18:0/22:6)	PE	0.02	0.852	0.999
PE(P-18:1/18:1)	PE	0.20	0.209	0.894
PE(P-18:1/20:4)	PE	-0.15	0.517	0.900
PE(P-18:1/22:6)	PE	-0.03	0.892	>0.999
PI(16:0/18:1)	PI	N.D.		
PI(18:0/20:3)	PI	N.D.		
PI(18:0/20:4)	PI	0.12	0.441	0.894
SM(14:0)	SM	0.16	0.229	0.894
SM(16:0)	SM	0.16	0.209	0.894
SM(18:0)	SM	0.30	0.006	0.894
SM(18:1)	SM	0.23	0.044	0.894
SM(20:0)	SM	0.11	0.382	0.894
SM(20:1)	SM	0.14	0.298	0.894
SM(22:0)	SM	0.05	0.732	0.971
SM(22:1)	SM	0.13	0.391	0.894
SM(24:0)	SM	0.05	0.409	0.894
SM(24:1)	SM	0.15	0.277	0.894
SM(26:0)	SM	0.03	0.582	0.939
SM(26:1)	SM	0.00	0.983	>0.999
TAG36:0-FA12:0	TAG	-0.16	0.741	0.972
TAG38:0-FA12:0	TAG	-0.53	0.575	0.939

TAG40:0-FA12:0	TAG	-1.66	0.448	0.894
TAG40:0-FA14:0	TAG	-1.64	0.421	0.894
TAG40:0-FA16:0	TAG	-1.52	0.437	0.894
TAG42:0-FA12:0	TAG	-2.49	0.511	0.894
TAG42:0-FA14:0	TAG	-2.58	0.512	0.894
TAG42:0-FA16:0	TAG	-1.67	0.444	0.894
TAG42:1-FA12:0	TAG	N.D.		
TAG42:1-FA14:0	TAG	N.D.		
TAG42:1-FA16:0	TAG	N.D.		
TAG42:1-FA16:1	TAG	N.D.		
TAG42:1-FA18:1	TAG	N.D.		
TAG42:2-FA12:0	TAG	N.D.		
TAG42:2-FA18:2	TAG	N.D.		
TAG44:0-FA12:0	TAG	-4.34	0.496	0.894
TAG44:0-FA14:0	TAG	-1.89	0.310	0.894
TAG44:0-FA16:0	TAG	-2.30	0.343	0.894
TAG44:0-FA18:0	TAG	-4.85	0.426	0.894
TAG44:1-FA12:0	TAG	-4.95	0.423	0.894
TAG44:1-FA14:0	TAG	-4.03	0.470	0.894
TAG44:1-FA14:1	TAG	N.D.		
TAG44:1-FA16:0	TAG	-4.03	0.345	0.894

TAG44:1-FA16:1	TAG	N.D.		
TAG44:1-FA18:1	TAG	N.D.		
TAG44:2-FA12:0	TAG	N.D.		
TAG44:2-FA14:0	TAG	N.D.		
TAG44:2-FA16:0	TAG	-0.27	0.604	0.939
TAG44:2-FA16:1	TAG	N.D.		
TAG44:2-FA18:1	TAG	N.D.		
TAG44:2-FA18:2	TAG	N.D.		
TAG44:3-FA18:2	TAG	N.D.		
TAG45:0-FA14:0	TAG	-1.02	0.257	0.894
TAG45:0-FA15:0	TAG	-0.69	0.202	0.894
TAG45:0-FA16:0	TAG	-1.04	0.331	0.894
TAG45:1-FA15:0	TAG	N.D.		
TAG45:1-FA16:0	TAG	-1.67	0.424	0.894
TAG45:1-FA18:1	TAG	N.D.		
TAG46:0-FA12:0	TAG	N.D.		
TAG46:0-FA14:0	TAG	-1.53	0.344	0.894
TAG46:0-FA16:0	TAG	-1.19	0.332	0.894
TAG46:0-FA18:0	TAG	N.D.		
TAG46:1-FA12:0	TAG	-4.89	0.291	0.894
TAG46:1-FA14:0	TAG	-2.32	0.353	0.894

TAG46:1-FA14:1	TAG	N.D.		
TAG46:1-FA16:0	TAG	-2.32	0.385	0.894
TAG46:1-FA16:1	TAG	-1.69	0.114	0.894
TAG46:1-FA18:0	TAG	N.D.		
TAG46:1-FA18:1	TAG	-4.06	0.415	0.894
TAG46:2-FA12:0	TAG	-3.32	0.432	0.894
TAG46:2-FA14:0	TAG	-2.05	0.381	0.894
TAG46:2-FA14:1	TAG	0.01	0.957	>0.999
TAG46:2-FA16:0	TAG	-2.01	0.394	0.894
TAG46:2-FA16:1	TAG	-2.35	0.009	0.894
TAG46:2-FA18:1	TAG	-2.78	0.411	0.894
TAG46:2-FA18:2	TAG	-3.26	0.345	0.894
TAG46:3-FA12:0	TAG	N.D.		
TAG46:3-FA14:0	TAG	N.D.		
TAG46:3-FA14:1	TAG	-0.40	0.238	0.894
TAG46:3-FA16:1	TAG	N.D.		
TAG46:3-FA18:1	TAG	-0.34	0.184	0.894
TAG46:3-FA18:2	TAG	-0.35	0.474	0.894
TAG46:3-FA18:3	TAG	N.D.		
TAG46:4-FA18:2	TAG	N.D.		
TAG47:0-FA14:0	TAG	-1.43	0.411	0.894
TAG47:0-FA15:0	TAG	-0.77	0.158	0.894

TAG47:0-FA16:0	TAG	-0.58	0.249	0.894
TAG47:0-FA17:0	TAG	-0.97	0.502	0.894
TAG47:1-FA14:0	TAG	N.D.		
TAG47:1-FA15:0	TAG	-1.40	0.309	0.894
TAG47:1-FA16:0	TAG	-2.06	0.344	0.894
TAG47:1-FA16:1	TAG	-0.86	0.209	0.894
TAG47:1-FA17:0	TAG	N.D.		
TAG47:1-FA18:1	TAG	-3.57	0.459	0.894
TAG47:2-FA14:0	TAG	-0.67	0.421	0.894
TAG47:2-FA15:0	TAG	N.D.		
TAG47:2-FA16:1	TAG	-1.25	0.114	0.894
TAG47:2-FA18:1	TAG	-1.95	0.404	0.894
TAG47:2-FA18:2	TAG	N.D.		
TAG48:0-FA14:0	TAG	-1.86	0.461	0.894
TAG48:0-FA16:0	TAG	-0.80	0.269	0.894
TAG48:0-FA18:0	TAG	-1.92	0.365	0.894
TAG48:1-FA12:0	TAG	-3.41	0.448	0.894
TAG48:1-FA14:0	TAG	-1.42	0.498	0.894
TAG48:1-FA14:1	TAG	N.D.		
TAG48:1-FA16:0	TAG	-1.20	0.364	0.894
TAG48:1-FA16:1	TAG	-0.91	0.246	0.894

TAG48:1-FA18:0	TAG	-3.54	0.417	0.894
TAG48:1-FA18:1	TAG	-1.69	0.440	0.894
TAG48:2-FA12:0	TAG	-2.72	0.356	0.894
TAG48:2-FA14:0	TAG	-0.38	0.685	0.961
TAG48:2-FA14:1	TAG	-0.77	0.471	0.894
TAG48:2-FA16:0	TAG	-0.25	0.742	0.972
TAG48:2-FA16:1	TAG	-0.81	0.291	0.894
TAG48:2-FA18:0	TAG	N.D.		
TAG48:2-FA18:1	TAG	-1.63	0.491	0.894
TAG48:2-FA18:2	TAG	-0.62	0.631	0.948
TAG48:3-FA12:0	TAG	-1.46	0.377	0.894
TAG48:3-FA14:0	TAG	-0.33	0.492	0.894
TAG48:3-FA14:1	TAG	0.14	0.244	0.894
TAG48:3-FA16:0	TAG	-0.03	0.949	>0.999
TAG48:3-FA16:1	TAG	-0.67	0.083	0.894
TAG48:3-FA18:1	TAG	-1.21	0.474	0.894
TAG48:3-FA18:2	TAG	-0.93	0.486	0.894
TAG48:3-FA18:3	TAG	N.D.		
TAG48:4-FA12:0	TAG	N.D.		
TAG48:4-FA16:1	TAG	-0.44	0.201	0.894
TAG48:4-FA18:1	TAG	N.D.		
TAG48:4-FA18:2	TAG	N.D.		

TAG48:4-FA18:3	TAG	N.D.		
TAG49:0-FA15:0	TAG	-1.10	0.442	0.894
TAG49:0-FA16:0	TAG	-0.88	0.250	0.894
TAG49:0-FA17:0	TAG	-0.46	0.299	0.894
TAG49:0-FA18:0	TAG	N.D.		
TAG49:1-FA14:0	TAG	N.D.		
TAG49:1-FA15:0	TAG	-1.99	0.196	0.894
TAG49:1-FA16:0	TAG	-1.37	0.324	0.894
TAG49:1-FA16:1	TAG	-1.18	0.249	0.894
TAG49:1-FA17:0	TAG	-1.56	0.375	0.894
TAG49:1-FA18:1	TAG	-1.56	0.352	0.894
TAG49:2-FA14:0	TAG	N.D.		
TAG49:2-FA15:0	TAG	-0.96	0.229	0.894
TAG49:2-FA16:0	TAG	-1.31	0.322	0.894
TAG49:2-FA16:1	TAG	-1.76	0.061	0.894
TAG49:2-FA17:0	TAG	N.D.		
TAG49:2-FA18:1	TAG	-2.50	0.297	0.894
TAG49:2-FA18:2	TAG	N.D.		
TAG49:3-FA16:0	TAG	N.D.		
TAG49:3-FA16:1	TAG	N.D.		
TAG49:3-FA18:2	TAG	N.D.		

TAG50:0-FA14:0	TAG	-1.24	0.459	0.894
TAG50:0-FA16:0	TAG	-0.69	0.263	0.894
TAG50:0-FA18:0	TAG	-0.96	0.250	0.894
TAG50:1-FA14:0	TAG	-2.14	0.375	0.894
TAG50:1-FA16:0	TAG	-0.93	0.386	0.894
TAG50:1-FA16:1	TAG	-0.72	0.521	0.904
TAG50:1-FA18:0	TAG	-1.51	0.388	0.894
TAG50:1-FA18:1	TAG	-1.16	0.387	0.894
TAG50:1-FA20:1	TAG	N.D.		
TAG50:2-FA14:0	TAG	-0.72	0.588	0.939
TAG50:2-FA14:1	TAG	0.03	0.857	0.999
TAG50:2-FA16:0	TAG	-0.31	0.577	0.939
TAG50:2-FA16:1	TAG	-0.54	0.423	0.894
TAG50:2-FA18:0	TAG	-1.25	0.399	0.894
TAG50:2-FA18:1	TAG	-0.95	0.355	0.894
TAG50:2-FA18:2	TAG	0.15	0.827	0.999
TAG50:2-FA20:2	TAG	N.D.		
TAG50:3-FA14:0	TAG	0.18	0.800	0.991
TAG50:3-FA14:1	TAG	-0.26	0.578	0.939
TAG50:3-FA16:0	TAG	0.34	0.393	0.894
TAG50:3-FA16:1	TAG	-0.31	0.466	0.894
TAG50:3-FA18:0	TAG	N.D.		

TAG50:3-FA18:1	TAG	-0.35	0.398	0.894
TAG50:3-FA18:2	TAG	-0.17	0.756	0.972
TAG50:3-FA18:3	TAG	N.D.		
TAG50:4-FA14:0	TAG	0.15	0.688	0.961
TAG50:4-FA14:1	TAG	N.D.		
TAG50:4-FA16:0	TAG	0.13	0.591	0.939
TAG50:4-FA16:1	TAG	-0.13	0.600	0.939
TAG50:4-FA18:1	TAG	-0.08	0.855	0.999
TAG50:4-FA18:2	TAG	0.35	0.374	0.894
TAG50:4-FA18:3	TAG	0.37	0.363	0.894
TAG50:5-FA14:1	TAG	-0.22	0.507	0.894
TAG50:5-FA16:0	TAG	N.D.		
TAG50:5-FA20:5	TAG	N.D.		
TAG51:0-FA16:0	TAG	-1.52	0.361	0.894
TAG51:0-FA17:0	TAG	-1.26	0.163	0.894
TAG51:0-FA18:0	TAG	-0.90	0.392	0.894
TAG51:1-FA15:0	TAG	N.D.		
TAG51:1-FA16:0	TAG	-1.51	0.355	0.894
TAG51:1-FA17:0	TAG	-0.18	0.575	0.939
TAG51:1-FA18:0	TAG	-0.95	0.284	0.894
TAG51:1-FA18:1	TAG	-1.43	0.401	0.894

TAG51:2-FA15:0	TAG	-1.12	0.417	0.894
TAG51:2-FA16:0	TAG	-0.04	0.459	0.894
TAG51:2-FA16:1	TAG	-2.62	0.281	0.894
TAG51:2-FA17:0	TAG	-0.79	0.209	0.894
TAG51:2-FA18:0	TAG	0.00	0.985	>0.999
TAG51:2-FA18:1	TAG	-0.09	0.404	0.894
TAG51:2-FA18:2	TAG	-1.36	0.425	0.894
TAG51:3-FA15:0	TAG	0.27	0.615	0.939
TAG51:3-FA16:0	TAG	0.01	0.729	0.969
TAG51:3-FA16:1	TAG	-0.83	0.173	0.894
TAG51:3-FA17:0	TAG	N.D.		
TAG51:3-FA18:1	TAG	0.00	0.855	0.999
TAG51:3-FA18:2	TAG	-0.34	0.613	0.939
TAG51:4-FA16:0	TAG	0.02	0.445	0.894
TAG51:4-FA16:1	TAG	0.08	0.302	0.894
TAG51:4-FA18:1	TAG	0.00	0.898	>0.999
TAG51:4-FA18:2	TAG	-0.05	0.752	0.972
TAG51:5-FA18:2	TAG	N.D.		
TAG52:0-FA16:0	TAG	-0.61	0.154	0.894
TAG52:0-FA18:0	TAG	-0.63	0.171	0.894
TAG52:0-FA20:0	TAG	N.D.		
TAG52:1-FA16:0	TAG	-1.29	0.352	0.894

TAG52:1-FA16:1	TAG	N.D.		
TAG52:1-FA18:0	TAG	-1.64	0.352	0.894
TAG52:1-FA18:1	TAG	-1.36	0.350	0.894
TAG52:1-FA20:0	TAG	N.D.		
TAG52:1-FA20:1	TAG	N.D.		
TAG52:2-FA14:0	TAG	N.D.		
TAG52:2-FA16:0	TAG	-0.64	0.384	0.894
TAG52:2-FA16:1	TAG	-0.47	0.499	0.894
TAG52:2-FA18:0	TAG	-0.46	0.534	0.911
TAG52:2-FA18:1	TAG	-0.79	0.371	0.894
TAG52:2-FA18:2	TAG	-0.02	0.978	>0.999
TAG52:2-FA20:0	TAG	N.D.		
TAG52:2-FA20:1	TAG	-2.05	0.384	0.894
TAG52:2-FA20:2	TAG	N.D.		
TAG52:3-FA14:0	TAG	N.D.		
TAG52:3-FA16:0	TAG	-0.04	0.928	>0.999
TAG52:3-FA16:1	TAG	-0.26	0.610	0.939
TAG52:3-FA18:0	TAG	0.13	0.705	0.961
TAG52:3-FA18:1	TAG	-0.25	0.606	0.939
TAG52:3-FA18:2	TAG	-0.15	0.774	0.986
TAG52:3-FA18:3	TAG	-0.18	0.847	0.999

TAG52:3-FA20:0	TAG	N.D.		
TAG52:3-FA20:1	TAG	N.D.		
TAG52:3-FA20:2	TAG	N.D.		
TAG52:3-FA20:3	TAG	-0.02	0.964	>0.999
TAG52:4-FA14:0	TAG	N.D.		
TAG52:4-FA16:0	TAG	0.23	0.621	0.944
TAG52:4-FA16:1	TAG	0.44	0.284	0.894
TAG52:4-FA18:0	TAG	N.D.		
TAG52:4-FA18:1	TAG	-0.06	0.872	0.999
TAG52:4-FA18:2	TAG	0.10	0.829	0.999
TAG52:4-FA18:3	TAG	0.15	0.683	0.961
TAG52:4-FA20:2	TAG	N.D.		
TAG52:4-FA20:3	TAG	0.02	0.937	>0.999
TAG52:4-FA20:4	TAG	N.D.		
TAG52:4-FA22:4	TAG	N.D.		
TAG52:5-FA14:0	TAG	N.D.		
TAG52:5-FA16:0	TAG	0.34	0.364	0.894
TAG52:5-FA16:1	TAG	0.82	0.069	0.894
TAG52:5-FA18:1	TAG	0.05	0.817	0.999
TAG52:5-FA18:2	TAG	0.07	0.777	0.986
TAG52:5-FA18:3	TAG	0.54	0.154	0.894

TAG52:5-FA20:4	TAG	N.D.		
TAG52:5-FA20:5	TAG	0.04	0.901	>0.999
TAG52:5-FA22:5	TAG	-0.93	0.342	0.894
TAG52:6-FA14:0	TAG	-0.03	0.943	>0.999
TAG52:6-FA16:0	TAG	N.D.		
TAG52:6-FA18:1	TAG	-0.63	0.143	0.894
TAG52:6-FA18:2	TAG	0.39	0.287	0.894
TAG52:6-FA18:3	TAG	N.D.		
TAG52:6-FA20:4	TAG	N.D.		
TAG52:6-FA20:5	TAG	N.D.		
TAG52:6-FA22:6	TAG	N.D.		
TAG52:7-FA16:0	TAG	-1.42	0.410	0.894
TAG52:7-FA18:1	TAG	N.D.		
TAG52:7-FA20:5	TAG	N.D.		
TAG52:7-FA22:6	TAG	N.D.		
TAG52:8-FA16:1	TAG	N.D.		
TAG53:0-FA16:0	TAG	-2.07	0.285	0.894
TAG53:1-FA16:0	TAG	N.D.		
TAG53:1-FA17:0	TAG	-0.19	0.264	0.894
TAG53:1-FA18:0	TAG	-1.02	0.329	0.894
TAG53:1-FA18:1	TAG	-1.78	0.414	0.894

TAG53:2-FA16:0	TAG	-4.10	0.443	0.894
TAG53:2-FA17:0	TAG	-0.13	0.505	0.894
TAG53:2-FA18:0	TAG	-0.01	0.827	0.999
TAG53:2-FA18:1	TAG	-0.23	0.346	0.894
TAG53:2-FA18:2	TAG	N.D.		
TAG53:3-FA16:0	TAG	-1.24	0.358	0.894
TAG53:3-FA17:0	TAG	-0.01	0.937	>0.999
TAG53:3-FA18:0	TAG	0.02	0.323	0.894
TAG53:3-FA18:1	TAG	-0.02	0.372	0.894
TAG53:3-FA18:2	TAG	-0.49	0.344	0.894
TAG53:4-FA16:0	TAG	N.D.		
TAG53:4-FA17:0	TAG	0.06	0.496	0.894
TAG53:4-FA18:0	TAG	-0.03	0.232	0.894
TAG53:4-FA18:1	TAG	0.03	0.102	0.894
TAG53:4-FA18:2	TAG	-0.01	0.586	0.939
TAG53:4-FA18:3	TAG	N.D.		
TAG53:5-FA18:1	TAG	0.00	0.854	0.999
TAG53:5-FA18:2	TAG	0.00	0.994	>0.999
TAG53:5-FA18:3	TAG	-0.03	0.389	0.894
TAG53:6-FA18:1	TAG	0.01	0.692	0.961
TAG53:6-FA18:2	TAG	-0.01	0.756	0.972
TAG53:6-FA18:3	TAG	-0.01	0.531	0.909

TAG53:7-FA18:3	TAG	-0.01	0.683	0.961
TAG54:0-FA16:0	TAG	-0.21	0.879	>0.999
TAG54:0-FA18:0	TAG	-0.25	0.097	0.894
TAG54:1-FA16:0	TAG	-2.44	0.433	0.894
TAG54:1-FA18:0	TAG	-0.67	0.456	0.894
TAG54:1-FA18:1	TAG	-0.12	0.431	0.894
TAG54:1-FA20:0	TAG	-3.20	0.184	0.894
TAG54:1-FA20:1	TAG	N.D.		
TAG54:2-FA16:0	TAG	-0.92	0.579	0.939
TAG54:2-FA18:0	TAG	-0.59	0.343	0.894
TAG54:2-FA18:1	TAG	-0.82	0.349	0.894
TAG54:2-FA18:2	TAG	-0.11	0.811	0.998
TAG54:2-FA20:0	TAG	N.D.		
TAG54:2-FA20:1	TAG	-0.68	0.386	0.894
TAG54:2-FA20:2	TAG	N.D.		
TAG54:3-FA16:0	TAG	-0.03	0.970	>0.999
TAG54:3-FA16:1	TAG	-0.63	0.599	0.939
TAG54:3-FA18:0	TAG	-0.04	0.909	>0.999
TAG54:3-FA18:1	TAG	-0.36	0.472	0.894
TAG54:3-FA18:2	TAG	-0.23	0.594	0.939
TAG54:3-FA18:3	TAG	N.D.		

TAG54:3-FA20:1	TAG	0.29	0.717	0.961
TAG54:3-FA20:2	TAG	-0.07	0.783	0.986
TAG54:4-FA16:0	TAG	0.15	0.790	0.986
TAG54:4-FA16:1	TAG	N.D.		
TAG54:4-FA18:0	TAG	0.45	0.189	0.894
TAG54:4-FA18:1	TAG	-0.03	0.938	>0.999
TAG54:4-FA18:2	TAG	-0.09	0.832	0.999
TAG54:4-FA18:3	TAG	-0.01	0.955	>0.999
TAG54:4-FA20:2	TAG	N.D.		
TAG54:4-FA20:3	TAG	0.34	0.460	0.894
TAG54:4-FA20:4	TAG	N.D.		
TAG54:5-FA16:0	TAG	0.16	0.699	0.961
TAG54:5-FA16:1	TAG	N.D.		
TAG54:5-FA18:0	TAG	N.D.		
TAG54:5-FA18:1	TAG	0.07	0.871	0.999
TAG54:5-FA18:2	TAG	0.08	0.850	0.999
TAG54:5-FA18:3	TAG	0.23	0.460	0.894
TAG54:5-FA20:3	TAG	0.44	0.222	0.894
TAG54:5-FA20:4	TAG	0.35	0.480	0.894
TAG54:5-FA20:5	TAG	N.D.		
TAG54:5-FA22:5	TAG	-0.24	0.538	0.911
TAG54:6-FA16:0	TAG	-0.06	0.920	>0.999

TAG54:6-FA16:1	TAG	N.D.		
TAG54:6-FA18:1	TAG	0.60	0.101	0.894
TAG54:6-FA18:2	TAG	0.42	0.451	0.894
TAG54:6-FA18:3	TAG	0.37	0.229	0.894
TAG54:6-FA20:4	TAG	0.56	0.412	0.894
TAG54:6-FA20:5	TAG	-0.51	0.482	0.894
TAG54:6-FA22:5	TAG	0.05	0.857	0.999
TAG54:6-FA22:6	TAG	-0.03	0.969	>0.999
TAG54:7-FA18:1	TAG	-1.93	0.463	0.894
TAG54:7-FA18:2	TAG	0.09	0.893	>0.999
TAG54:7-FA18:3	TAG	-0.25	0.609	0.939
TAG54:7-FA20:4	TAG	N.D.		
TAG54:7-FA20:5	TAG	N.D.		
TAG54:7-FA22:5	TAG	N.D.		
TAG54:7-FA22:6	TAG	0.27	0.699	0.961
TAG54:8-FA20:5	TAG	N.D.		
TAG54:8-FA22:6	TAG	N.D.		
TAG55:1-FA16:0	TAG	N.D.		
TAG55:1-FA18:1	TAG	N.D.		
TAG55:2-FA18:1	TAG	-3.38	0.203	0.894
TAG55:2-FA18:2	TAG	N.D.		

TAG55:3-FA18:1	TAG	-1.66	0.504	0.894
TAG55:3-FA18:2	TAG	N.D.		
TAG55:4-FA18:1	TAG	-0.65	0.357	0.894
TAG55:4-FA18:2	TAG	N.D.		
TAG55:5-FA18:1	TAG	-0.03	0.537	0.911
TAG55:5-FA18:2	TAG	-0.12	0.553	0.921
TAG55:6-FA18:1	TAG	-0.03	0.187	0.894
TAG55:6-FA20:3	TAG	0.02	0.453	0.894
TAG55:6-FA20:4	TAG	0.00	0.989	>0.999
TAG55:7-FA18:1	TAG	-0.03	0.188	0.894
TAG55:7-FA20:3	TAG	0.03	0.297	0.894
TAG55:7-FA20:4	TAG	-0.03	0.165	0.894
TAG55:7-FA22:6	TAG	N.D.		
TAG55:8-FA20:4	TAG	-0.04	0.154	0.894
TAG56:1-FA16:0	TAG	N.D.		
TAG56:1-FA18:1	TAG	-0.03	0.727	0.969
TAG56:10-FA18:2	TAG	N.D.		
TAG56:2-FA16:0	TAG	N.D.		
TAG56:2-FA18:0	TAG	-0.22	0.288	0.894
TAG56:2-FA18:1	TAG	-0.03	0.413	0.894
TAG56:2-FA20:0	TAG	-1.58	0.451	0.894
TAG56:2-FA20:1	TAG	-0.20	0.341	0.894

TAG56:3-FA16:0	TAG	N.D.		
TAG56:3-FA18:0	TAG	-0.58	0.692	0.961
TAG56:3-FA18:1	TAG	-0.13	0.416	0.894
TAG56:3-FA18:2	TAG	-0.56	0.613	0.939
TAG56:3-FA20:0	TAG	N.D.		
TAG56:3-FA20:1	TAG	-0.16	0.616	0.939
TAG56:3-FA20:2	TAG	0.03	0.706	0.961
TAG56:4-FA16:0	TAG	N.D.		
TAG56:4-FA18:0	TAG	N.D.		
TAG56:4-FA18:1	TAG	-0.31	0.526	0.906
TAG56:4-FA18:2	TAG	0.30	0.503	0.894
TAG56:4-FA20:1	TAG	-0.02	0.903	>0.999
TAG56:4-FA20:2	TAG	0.11	0.436	0.894
TAG56:4-FA20:3	TAG	N.D.		
TAG56:5-FA16:0	TAG	N.D.		
TAG56:5-FA18:0	TAG	N.D.		
TAG56:5-FA18:1	TAG	0.05	0.860	0.999
TAG56:5-FA18:2	TAG	0.85	0.426	0.894
TAG56:5-FA20:1	TAG	0.19	0.770	0.986
TAG56:5-FA20:2	TAG	-0.05	0.749	0.972
TAG56:5-FA20:3	TAG	0.12	0.778	0.986

TAG56:5-FA20:4	TAG	0.83	0.192	0.894
TAG56:5-FA22:4	TAG	N.D.		
TAG56:5-FA22:5	TAG	-0.74	0.443	0.894
TAG56:6-FA16:0	TAG	0.38	0.355	0.894
TAG56:6-FA18:0	TAG	N.D.		
TAG56:6-FA18:1	TAG	0.35	0.253	0.894
TAG56:6-FA18:2	TAG	N.D.		
TAG56:6-FA18:3	TAG	N.D.		
TAG56:6-FA20:2	TAG	N.D.		
TAG56:6-FA20:3	TAG	N.D.		
TAG56:6-FA20:4	TAG	0.28	0.555	0.921
TAG56:6-FA20:5	TAG	N.D.		
TAG56:6-FA22:4	TAG	N.D.		
TAG56:6-FA22:5	TAG	-0.55	0.414	0.894
TAG56:6-FA22:6	TAG	-1.37	0.393	0.894
TAG56:7-FA16:0	TAG	0.58	0.235	0.894
TAG56:7-FA18:0	TAG	N.D.		
TAG56:7-FA18:1	TAG	-0.01	0.986	>0.999
TAG56:7-FA18:2	TAG	0.82	0.041	0.894
TAG56:7-FA20:4	TAG	0.40	0.414	0.894
TAG56:7-FA20:5	TAG	0.13	0.923	>0.999

TAG56:7-FA22:5	TAG	0.26	0.482	0.894
TAG56:7-FA22:6	TAG	0.18	0.751	0.972
TAG56:8-FA16:0	TAG	-1.01	0.458	0.894
TAG56:8-FA16:1	TAG	N.D.		
TAG56:8-FA18:1	TAG	N.D.		
TAG56:8-FA18:2	TAG	0.56	0.158	0.894
TAG56:8-FA20:4	TAG	N.D.		
TAG56:8-FA20:5	TAG	-1.74	0.330	0.894
TAG56:8-FA22:5	TAG	N.D.		
TAG56:8-FA22:6	TAG	0.44	0.445	0.894
TAG56:9-FA20:5	TAG	N.D.		
TAG56:9-FA22:6	TAG	N.D.		
TAG57:2-FA18:1	TAG	-0.77	0.399	0.894
TAG57:8-FA22:6	TAG	0.01	0.867	0.999
TAG57:9-FA22:6	TAG	-0.02	0.422	0.894
TAG58:10-FA18:2	TAG	N.D.		
TAG58:10-FA20:5	TAG	N.D.		
TAG58:10-FA22:6	TAG	N.D.		
TAG58:2-FA18:1	TAG	-0.70	0.416	0.894
TAG58:3-FA18:1	TAG	-0.12	0.753	0.972
TAG58:5-FA18:1	TAG	N.D.		

TAG58:6-FA16:0	TAG	N.D.		
TAG58:6-FA18:1	TAG	0.03	0.954	>0.999
TAG58:6-FA22:4	TAG	N.D.		
TAG58:6-FA22:5	TAG	-0.10	0.710	0.961
TAG58:7-FA16:0	TAG	N.D.		
TAG58:7-FA18:0	TAG	N.D.		
TAG58:7-FA18:1	TAG	0.24	0.676	0.961
TAG58:7-FA18:2	TAG	0.07	0.585	0.939
TAG58:7-FA22:5	TAG	0.07	0.909	>0.999
TAG58:7-FA22:6	TAG	-0.16	0.780	0.986
TAG58:8-FA18:1	TAG	0.27	0.739	0.972
TAG58:8-FA18:2	TAG	N.D.		
TAG58:8-FA22:5	TAG	N.D.		
TAG58:8-FA22:6	TAG	0.39	0.466	0.894
TAG58:9-FA18:1	TAG	-1.39	0.059	0.894
TAG58:9-FA18:2	TAG	N.D.		
TAG58:9-FA20:4	TAG	N.D.		
TAG58:9-FA22:5	TAG	0.14	0.665	0.961
TAG58:9-FA22:6	TAG	0.44	0.435	0.894
TAG60:10-FA22:5	TAG	N.D.		
TAG60:11-FA22:5	TAG	N.D.		

CE, cholesteryl ester; CER, ceramide; DAG, diacylglycerol; DCER, dihydroceramide; FFA, free fatty acid; HCER, hexosylceramide; LCER, lactosylceramide; LPC, lysophosphatidylcholine; LPE, lysophosphatidylethanolamine; MAG, monoacylglycerol; N.D., not detected; PC, phosphatidylcholine; PE, phosphatidylethanolamine; PI, phosphatidylinositol; SM, sphingomyelin; TAG, triacylglycerol.

Supplementary Table S4. Differentially expressed proteins between the slow and fast progression groups.

Protein	Protein description	Fold change	p-value	q-value
GPC1	Glypican-1	1.86	0.001	0.066
GFAP	Glial fibrillary acidic protein	1.77	0.000	0.040
GPNMB	Transmembrane glycoprotein NMB	1.76	0.000	0.037
B4GT1	Beta-1,4-Galactosyltransferase 1	1.41	0.000	0.045
MGAT2	Alpha-1,6-mannosyl-glycoprotein 2-beta-N-acetylglucosaminyltransferase	1.41	0.001	0.096
UCHL1	Ubiquitin Carboxy-Terminal Hydrolase L1	1.39	0.000	0.045
TMED4	Transmembrane P24 Trafficking Protein 4	1.37	0.000	0.045
GPR56	G-protein coupled receptor 56	1.32	0.001	0.096
FAM198B	Family with sequence similarity 198, member B	1.29	0.001	0.081
POMGNT1	protein O-linked mannose beta1,2-N-acetylglucosaminyltransferase	1.27	0.000	0.045
CAPG	Macrophage-capping protein	1.26	0.001	0.096
TGBR3	TGF beta receptor type 3	1.16	0.000	0.045
CXL16	C-X-C motif chemokine 16 protein	1.16	0.001	0.084

The differentially expressed proteins ($p < 0.05$, $q < 0.1$) were analyzed using Welch's t-test.

Supplementary Table S5. Differentially expressed metabolites between the slow and fast progression groups.

Metabolite	Class	Fold change	p-value	q-value
ergothioneine	Xenobiotics	2.14	0.036	0.359
p-cresol sulfate	Xenobiotics	2.11	0.014	0.359
X - 12411		2.09	0.000	0.088
4-methyl-2-oxopentanoate	Amino Acid	1.99	0.048	0.359
2-oxoarginine*	Amino Acid	1.92	0.024	0.359
phenol sulfate	Amino Acid	1.90	0.049	0.359
acetylcarnitine (C2)	Lipid	1.81	0.044	0.359
S-1-pyrroline-5-carboxylate	Amino Acid	1.69	0.038	0.359
phenylacetylglutamine	Peptide	1.67	0.026	0.359
acisoga	Amino Acid	1.59	0.009	0.359
pyruvate	Carbohydrate	1.57	0.049	0.359
kynurenate	Amino Acid	1.46	0.021	0.359
indoleacetate	Amino Acid	1.43	0.026	0.359
X - 17299		1.42	0.000	0.088
N-formylmethionine	Amino Acid	1.40	0.049	0.359
cis-4-decenoylcarnitine (C10:1)	Lipid	1.33	0.032	0.359
arabonate/xylonate	Carbohydrate	1.31	0.031	0.359
X - 12100		1.29	0.008	0.359
prolylhydroxyproline	Amino Acid	1.29	0.044	0.359

O-sulfo-L-tyrosine	Xenobiotics	1.28	0.006	0.359
propionylcarnitine (C3)	Lipid	1.27	0.021	0.359
cysteine	Amino Acid	1.26	0.023	0.359
gluconate	Xenobiotics	1.26	0.019	0.359
hydroxyasparagine	Amino Acid	1.24	0.012	0.359
5,6-dihydrouridine	Nucleotide	1.23	0.007	0.359
gamma-glutamylalanine	Peptide	1.23	0.049	0.359
isobutyrylcarnitine (C4)	Amino Acid	1.23	0.002	0.216
3-methylglutarylcarnitine (2)	Amino Acid	1.23	0.043	0.359
diacetylspermidine	Amino Acid	1.20	0.039	0.359
tiglyl carnitine (C5)	Amino Acid	1.18	0.020	0.359
dimethylarginine (ADMA + SDMA)	Amino Acid	1.18	0.012	0.359
7-methylguanine	Nucleotide	1.18	0.016	0.359
N2,N2-dimethylguanosine	Nucleotide	1.18	0.015	0.359
(N(1) + N(8))-acetylspermidine	Amino Acid	1.18	0.040	0.359
mannose	Carbohydrate	1.18	0.018	0.359
pseudouridine	Nucleotide	1.17	0.007	0.359
mannitol/sorbitol	Carbohydrate	1.16	0.044	0.359
2-methylcitrate/homocitrate	Energy	1.14	0.041	0.359
sulfate	Xenobiotics	1.13	0.015	0.359
gamma-glutamyl-alpha-lysine	Peptide	0.86	0.044	0.359
X - 12680		0.83	0.034	0.359

cys-gly, oxidized	Amino Acid	0.78	0.037	0.359
5-oxoproline	Amino Acid	0.75	0.039	0.359
tartronate (hydroxymalonate)	Xenobiotics	0.71	0.034	0.359
gamma-glutamyl-epsilon-lysine	Peptide	0.69	0.026	0.359
methionine sulfoxide	Amino Acid	0.69	0.020	0.359
cyclo(pro-val)	Peptide	0.67	0.026	0.359
X - 25416		0.66	0.019	0.359
aspartate	Amino Acid	0.63	0.028	0.359
paraxanthine	Xenobiotics	0.61	0.037	0.359
gamma-glutamylglutamate	Peptide	0.60	0.030	0.359
glutamate	Amino Acid	0.58	0.008	0.359
caffeine	Xenobiotics	0.42	0.033	0.359

The differentially expressed metabolites ($p < 0.05$) were analyzed using Welch's t-test.

Supplementary Table S6. Differentially expressed lipids between the slow and fast progression groups.

Lipid	Lipid class	Fold change	p-value	q-value
SM(18:0)	SM	1.23	0.006	0.894
PE(P-16:0/18:1)	PE	1.19	0.031	0.894
PC(16:0/16:1)	PC	1.19	0.038	0.894
PC(16:0/16:0)	PC	1.18	0.033	0.894
SM(18:1)	SM	1.17	0.044	0.894
DCER(22:0)	DCER	0.90	0.024	0.894
MAG(18:1)	MAG	0.70	0.048	0.894

The differentially expressed lipids ($p < 0.05$) were analyzed using Welch's t-test.

Supplementary Table S7. Significantly changed proteins between the slow and fast progression groups.

Protein	Fold change	p-value	q-value
DDR2	9.60	0.045	0.095
ACHE	4.95	0.025	0.069
FUCA1	4.78	0.040	0.090
GPC1	3.43	0.001	0.007
CLIC1	2.74	0.024	0.069
MGAT2	2.21	0.035	0.082
CD177	2.08	0.023	0.065
CHIT1	1.84	0.003	0.016
GFAP	1.75	0.001	0.006
APOC2	1.71	0.002	0.011
FUCA2	1.70	0.001	0.005
POSTN	1.69	0.032	0.079
GAPDH	1.64	0.029	0.076
MCFD2	1.63	0.000	0.000
APOC1	1.63	0.021	0.061
GPNMB	1.62	0.001	0.009
C16orf89	1.59	0.004	0.019
CHI3L2	1.57	0.000	0.000
CHST8	1.55	0.032	0.079
HBB	1.53	0.003	0.014

SORCS3	1.52	0.002	0.011
LDHB	1.52	0.000	0.000
GDA	1.49	0.000	0.002
GALNT10	1.48	0.024	0.068
LGALS1	1.47	0.009	0.034
MSLN	1.40	0.000	0.000
HBA1	1.40	0.000	0.001
TMEM132B	1.38	0.028	0.073
PRELP	1.37	0.005	0.023
APOE	1.36	0.000	0.000
TMED4	1.36	0.001	0.009
B4GALT1	1.36	0.007	0.028
HPRT1	1.36	0.004	0.019
UCHL1	1.34	0.000	0.000
GAA	1.33	0.041	0.090
PFN1	1.32	0.016	0.052
GOT2	1.32	0.001	0.007
LYZ	1.32	0.000	0.000
MDH1	1.30	0.000	0.000
ATP6AP2	1.29	0.000	0.000
DNAH5	1.28	0.002	0.011
TXNDC17	1.28	0.001	0.008

XYLT1	1.28	0.026	0.070
C1QTNF3	1.26	0.001	0.005
CRP	1.26	0.038	0.086
GSTP1	1.25	0.039	0.089
METRN	1.25	0.000	0.002
GALNT18	1.25	0.002	0.011
POMGNT1	1.24	0.000	0.001
CRABP1	1.23	0.000	0.004
FAM198B	1.23	0.001	0.009
MDH2	1.23	0.025	0.070
GPR56	1.22	0.018	0.054
SLC12A2	1.22	0.014	0.046
GSS	1.21	0.018	0.055
PRDX3	1.21	0.019	0.057
APOB	1.21	0.035	0.082
BST1	1.21	0.002	0.011
LRRN1	1.20	0.005	0.022
CHST15	1.20	0.001	0.008
GALNT13	1.20	0.028	0.073
C12orf49	1.20	0.000	0.004
HAVCR2	1.19	0.001	0.008

NUTF2	1.19	0.002	0.009
B3GAT3	1.19	0.015	0.049
DMKN	1.19	0.004	0.021
CAPG	1.18	0.006	0.026
PLTP	1.18	0.009	0.033
PPIA	1.18	0.000	0.004
GPI	1.17	0.027	0.071
MAN1A2	1.17	0.022	0.063
HTRA1	1.17	0.000	0.000
SMPDL3B	1.17	0.008	0.032
LRP8	1.17	0.041	0.090
COL6A2	1.16	0.009	0.034
OMD	1.16	0.000	0.001
CLSTN3	1.16	0.002	0.013
NRXN2	1.16	0.003	0.014
SERPINA3	1.15	0.000	0.000
YWHAG	1.15	0.001	0.008
CXCL16	1.14	0.018	0.054
GOT1	1.14	0.000	0.000
ST6GAL2	1.14	0.025	0.070
CECR1	1.14	0.040	0.089
ATP1A2	1.14	0.036	0.083

CLU	1.14	0.000	0.000
RELN	1.13	0.001	0.005
MAN2B1	1.13	0.041	0.090
TGFBR3	1.13	0.000	0.000
FBLN2	1.13	0.035	0.082
RNASET2	1.13	0.000	0.001
FAM20B	1.13	0.001	0.006
GDI1	1.12	0.006	0.026
SLC3A2	1.12	0.001	0.009
PITHD1	1.12	0.009	0.034
GNPTG	1.12	0.000	0.000
ISLR	1.12	0.007	0.027
MXRA8	1.12	0.000	0.002
CTSF	1.12	0.033	0.079
LMAN1	1.11	0.002	0.012
PGAM1	1.11	0.002	0.011
PTPRZ1	1.11	0.033	0.079
OGN	1.11	0.001	0.009
CD44	1.11	0.001	0.006
C2	1.11	0.000	0.004
PLXNB2	1.10	0.000	0.000

EFCAB14	1.10	0.043	0.093
HABP2	1.10	0.033	0.080
SNCG	1.10	0.017	0.052
B3GNT2	1.10	0.009	0.034
CPE	1.10	0.000	0.000
GDF11	1.10	0.044	0.093
SLC38A10	1.09	0.009	0.034
FAT2	1.09	0.010	0.036
MAN2A2	1.09	0.009	0.033
SCG5	1.09	0.000	0.001
ENPP2	1.09	0.048	0.099
TPI1	1.09	0.000	0.001
VASN	1.09	0.014	0.048
GM2A	1.09	0.007	0.028
ENO1	1.09	0.001	0.005
MAN1C1	1.09	0.034	0.081
CNTN2	1.08	0.002	0.011
CD14	1.08	0.009	0.034
DCN	1.08	0.028	0.073
GPX3	1.07	0.046	0.096
ALDOA	1.07	0.017	0.053
LRG1	1.07	0.026	0.071

CNTFR	1.07	0.031	0.077
NEO1	1.06	0.000	0.000
AXL	1.06	0.010	0.036
MCAM	1.06	0.001	0.007
CNDP1	1.06	0.018	0.054
CTSS	1.05	0.032	0.079
TBCA	1.05	0.029	0.074
ALDOC	1.04	0.032	0.079
SDF4	1.04	0.048	0.099
CADM4	0.97	0.024	0.068
SOD1	0.96	0.020	0.058
CANT1	0.96	0.004	0.020
COLEC12	0.96	0.026	0.071
CDH6	0.96	0.034	0.081
FAM3C	0.95	0.021	0.061
TGOLN2	0.95	0.000	0.004
SELENBP1	0.95	0.019	0.058
LPHN3	0.95	0.017	0.054
NCAM2	0.95	0.029	0.074
CFI	0.95	0.048	0.099
NUCB1	0.95	0.012	0.042

CFB	0.94	0.013	0.044
SCG3	0.94	0.009	0.034
MMP2	0.94	0.030	0.076
TYRO3	0.94	0.015	0.050
LSAMP	0.94	0.000	0.000
SCG2	0.94	0.031	0.077
KLK6	0.94	0.013	0.045
BSG	0.94	0.034	0.081
PPIB	0.94	0.006	0.025
PLG	0.93	0.044	0.093
DSG2	0.93	0.037	0.084
PCOLCE	0.93	0.000	0.002
CHL1	0.93	0.000	0.001
CLSTN1	0.93	0.001	0.009
PVRL1	0.93	0.044	0.093
LUM	0.93	0.010	0.037
CD99L2	0.93	0.014	0.047
C6	0.93	0.003	0.017
ITIH2	0.93	0.020	0.058
SERPINF1	0.93	0.000	0.001
NEGR1	0.92	0.031	0.078
RTN4RL2	0.92	0.006	0.026

SERPINI1	0.92	0.000	0.004
ATRN	0.92	0.003	0.014
LSR	0.92	0.003	0.014
CRTAC1	0.92	0.007	0.027
VGF	0.92	0.000	0.001
THY1	0.92	0.001	0.005
EFEMP2	0.92	0.040	0.090
NELL2	0.92	0.013	0.044
CADM1	0.92	0.015	0.049
PTGDS	0.91	0.041	0.090
LRP1	0.91	0.015	0.049
HPX	0.91	0.004	0.019
GPR37	0.91	0.034	0.082
IGFBP6	0.91	0.005	0.023
CFH	0.91	0.007	0.028
LAMB2	0.91	0.006	0.026
DSC2	0.91	0.026	0.071
C9	0.90	0.007	0.027
TNFRSF21	0.90	0.043	0.093
SCN3B	0.90	0.031	0.077
PTPRN	0.90	0.005	0.023

CDH13	0.90	0.000	0.000
GFRA2	0.90	0.005	0.022
VSIG4	0.89	0.004	0.019
AGA	0.89	0.000	0.003
FAM174A	0.89	0.023	0.065
EPHA10	0.89	0.008	0.031
ALCAM	0.89	0.001	0.008
C7	0.88	0.000	0.000
LAYN	0.88	0.047	0.098
PCDH7	0.88	0.003	0.016
APCS	0.87	0.046	0.096
C3	0.87	0.000	0.000
F2	0.87	0.000	0.003
CTBS	0.87	0.000	0.000
SOD3	0.87	0.006	0.025
SERPINC1	0.86	0.000	0.001
CHGA	0.86	0.000	0.000
DKK3	0.86	0.014	0.046
CPN1	0.86	0.043	0.093
CTSL	0.86	0.000	0.000
LGALS3BP	0.85	0.000	0.000
CTSH	0.85	0.002	0.011

GPLD1	0.85	0.011	0.039
TTR	0.85	0.000	0.002
AZGP1	0.85	0.001	0.005
GC	0.85	0.001	0.008
PTPRF	0.84	0.031	0.077
VSTM2A	0.83	0.026	0.070
CCK	0.83	0.016	0.052
PTK7	0.83	0.017	0.054
FGA	0.83	0.001	0.005
SIRPA	0.83	0.014	0.046
FCGBP	0.82	0.039	0.088
SEZ6L	0.82	0.002	0.009
PTHLH	0.82	0.044	0.093
PRNP	0.82	0.000	0.003
HS6ST3	0.81	0.000	0.001
COL5A2	0.81	0.044	0.093
FGB	0.80	0.001	0.009
MIS12	0.79	0.036	0.083
P4HB	0.79	0.000	0.004
COMP	0.78	0.008	0.032
DSC1	0.78	0.000	0.001

PON1	0.78	0.000	0.000
LIPA	0.77	0.035	0.082
IGFALS	0.77	0.000	0.000
NPTN	0.76	0.027	0.073
FGG	0.75	0.000	0.001
SHBG	0.75	0.001	0.007
RBP4	0.75	0.000	0.000
KRT2	0.73	0.021	0.061
CDH12	0.72	0.017	0.052
AMBP	0.71	0.002	0.013
UNC5D	0.68	0.003	0.017
MFAP4	0.65	0.008	0.031
CNTN5	0.64	0.040	0.090
PI16	0.63	0.000	0.000
CTSO	0.62	0.008	0.033
GALNT6	0.62	0.029	0.074
HRG	0.59	0.000	0.000
FCN3	0.58	0.037	0.085
NPDC1	0.45	0.010	0.036
KRT16	0.39	0.021	0.061
CPSF1	0.30	0.035	0.082
EGFR	0.26	0.010	0.036

The significantly changed proteins ($p < 0.05$, $q < 0.1$) were analyzed using multivariate linear mixed model analysis considering age and sex.

Supplementary Table S8. Multivariate linear mixed model parameters for predicting the disease progression rate.

Factor	Model parameter	β	95% CI	p-value
Single protein and age	(Intercept)	1.59	-3.3 to 6.47	0.519
	Age	0.03	-0.01 to 0.07	0.175
	ALSFRS.R	-0.05	-0.14 to 0.03	0.213
	GPNMB	0.58	0.17 to 0.99	0.007
	(Intercept)	3.16	-1.95 to 8.28	0.221
	Age	0.03	-0.02 to 0.07	0.211
	ALSFRS.R	-0.09	-0.17 to -0.01	0.035
	GFAP	0.37	-0.04 to 0.78	0.078
	(Intercept)	2.44	-2.5 to 7.38	0.328
	Age	0.03	-0.01 to 0.07	0.158
	ALSFRS.R	-0.08	-0.16 to 0.01	0.069
	GPC1	0.47	0.07 to 0.87	0.021
	(Intercept)	1.41	-3.79 to 6.61	0.590
	Age	0.04	0 to 0.08	0.069
	ALSFRS.R	-0.07	-0.15 to 0.02	0.142
	MGAT2	0.33	-0.09 to 0.74	0.123
	(Intercept)	1.56	-3.26 to 6.39	0.519
	Age	0.05	0.01 to 0.09	0.022
	ALSFRS.R	-0.08	-0.16 to 0	0.041
	CH3L2	0.59	0.21 to 0.97	0.003

Multiple factors	(Intercept)	1.42	-3.26 to 6.09	0.546
	Age	0.03	-0.01 to 0.07	0.110
	ALSFRS.R	-0.06	-0.14 to 0.02	0.166
	CH3L2	0.41	0.01 to 0.8	0.043*
	GPC1	0.32	-0.06 to 0.71	0.100
	GPNMB	0.35	-0.08 to 0.77	0.107

Linear mixed model predictions were calculated by considering the z-score of each protein level and age. CI, confidence interval.

Supplementary Fig. S1. Comparison between the amyotrophic lateral sclerosis progression groups and control.

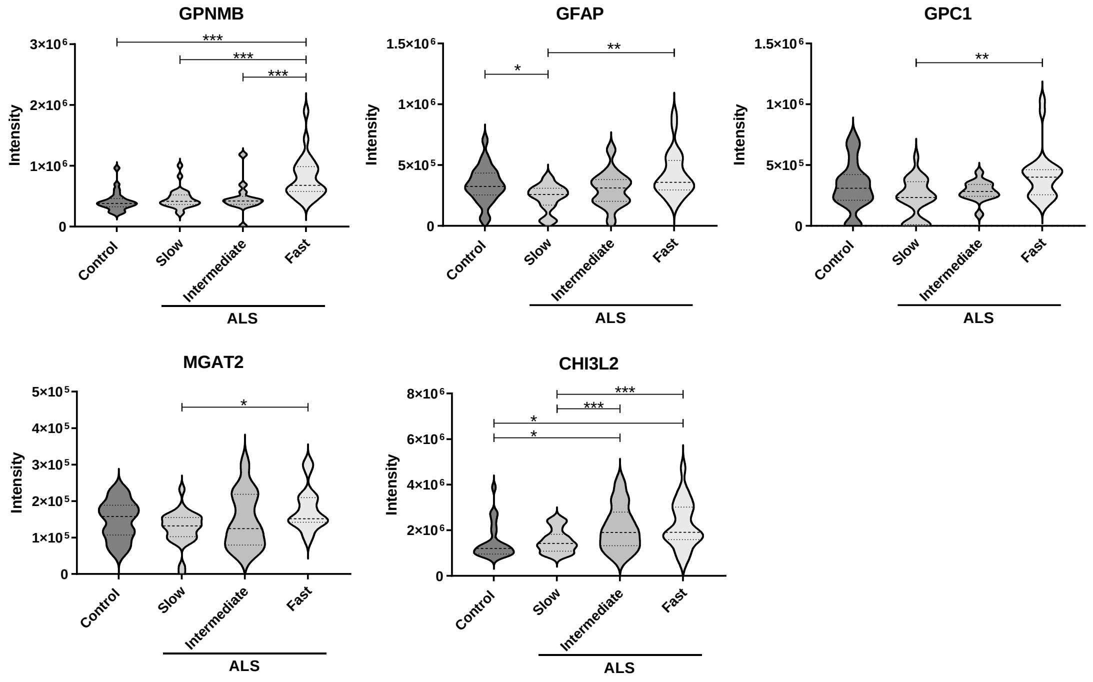

The violin plot represents the weight distributions of variables in each group. The dashed line in each plot represents the median, and the dotted line represents the 25th and 75th percentiles. *, $p < 0.05$; **, $p < 0.01$; ***, $p < 0.0001$, using multivariate regression analyses considering age.
